# Supplementary material for: Genetic influence on within-person longitudinal change in anthropometric traits in the UK Biobank
Source: Nat Commun. 2024 May 6;15:3776. doi: 10.1038/s41467-024-47802-7 (PMC11074304; doi:10.1038/s41467-024-47802-7)
Supplement: Supplementary file 1 — Supplementary Information [file 41467_2024_47802_MOESM1_ESM.pdf]

Supplementary material for

Genetic influence on within-person longitudinal change in anthropometric traits in the UK Biobank

Correspondence to: k.kemper@uq.edu.au or peter.visscher@uq.edu.au

This file contains:

Supplementary Figures S1-S5 2
Supplementary Tables S1-S7 7
Supplementary Note 1-6 14

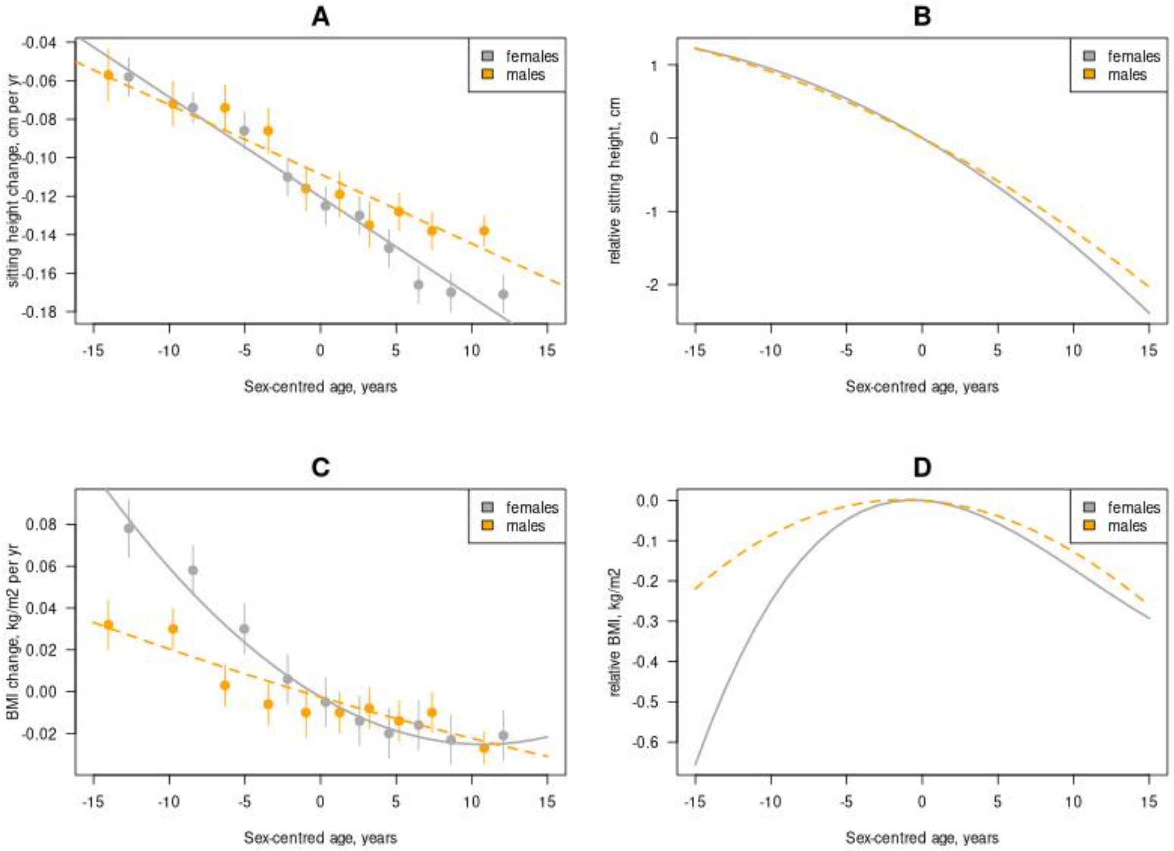

Supplementary Figure S1. Within-person trait change is dependent on age and sex for sitting height and body mass index.
Panels show sitting height (A) and body mass index (BMI, C) rate of change, where lines show the age- and sex-dependent polynomial fitted to the data. Points and vertical bars (95% CI) indicate the mean value of the rate of change for trait the in 10 (approximately) equal groups, grouped on average age of measurement for females (N = 25,759) and males (N = 24,313). Shown are the curves for cumulative sitting height (B, cm) and BMI (D, kg) obtained by integrating, with respect to age, the sex-specific regressions shown in A and C. The average age of measurement is approximately 59.0 and 60.3 years for females and males, respectively.

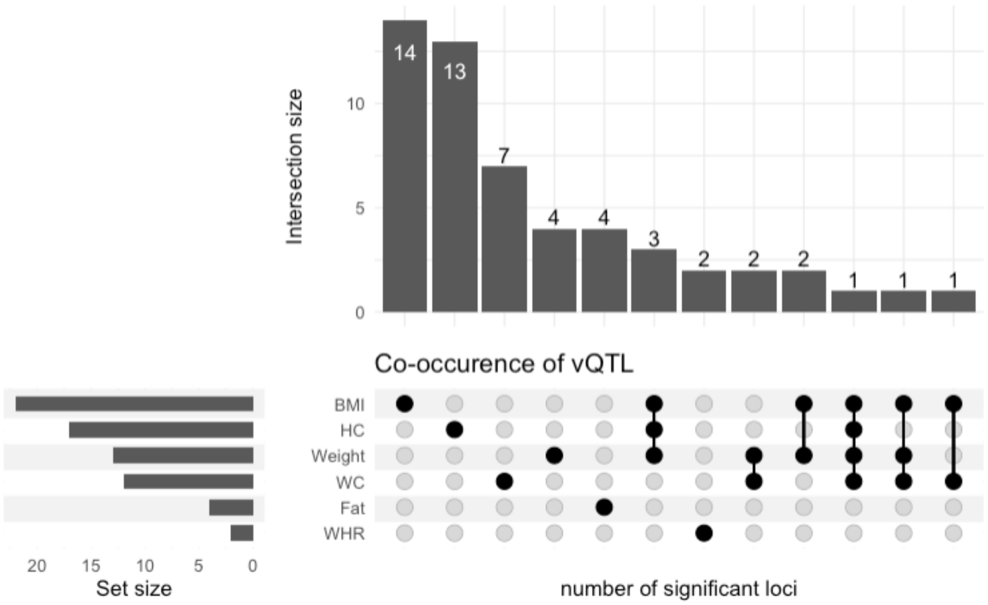

Supplementary Figure S2. The co-occurrence of genome-wide significant (P < $1x10^{-8}$) population-level variance controlling quantitative trait loci (vQTL) for 7 anthropometric traits.

Traits are body mass index (BMI), hip and waist circumference (HC, WC), weight, body fat percentage (Fat) and waist-to-hip ratio (WHR). There were no genome-wide significant loci for height and sitting height.

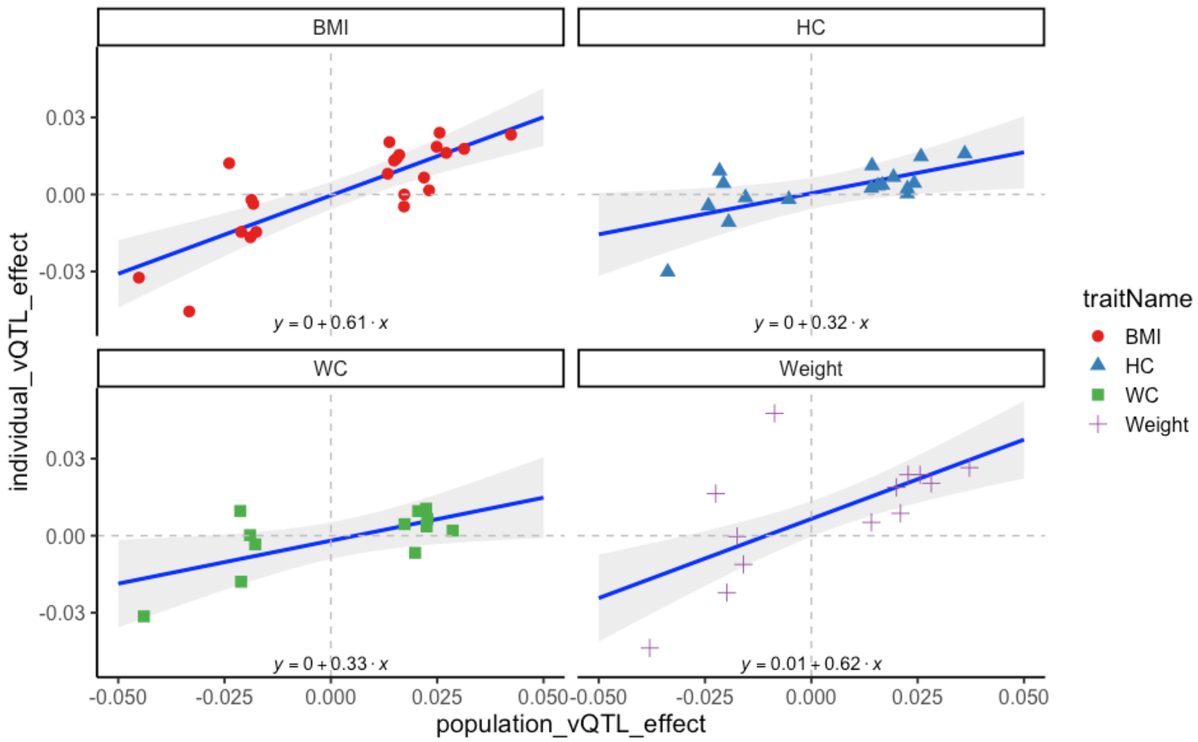

Supplementary Figure S3. Replication of population-level variance quantitative trait loci (vQTL) effects using variability within individuals.

Trait-independent regressions of vQTL effects for body mass index (BMI), hip and waist circumference (HC, WC) and weight. Regression coefficients for BMI (0.610, s.e. 0.098, P = 4.6×10^{-6}), HC (0.320, s.e. 0.092, P = 0.003), WC (0.335, s.e. 0.118, P = 0.017) and weight (0.617, s.e. 0.228, P = 0.020) were all significantly different from zero.

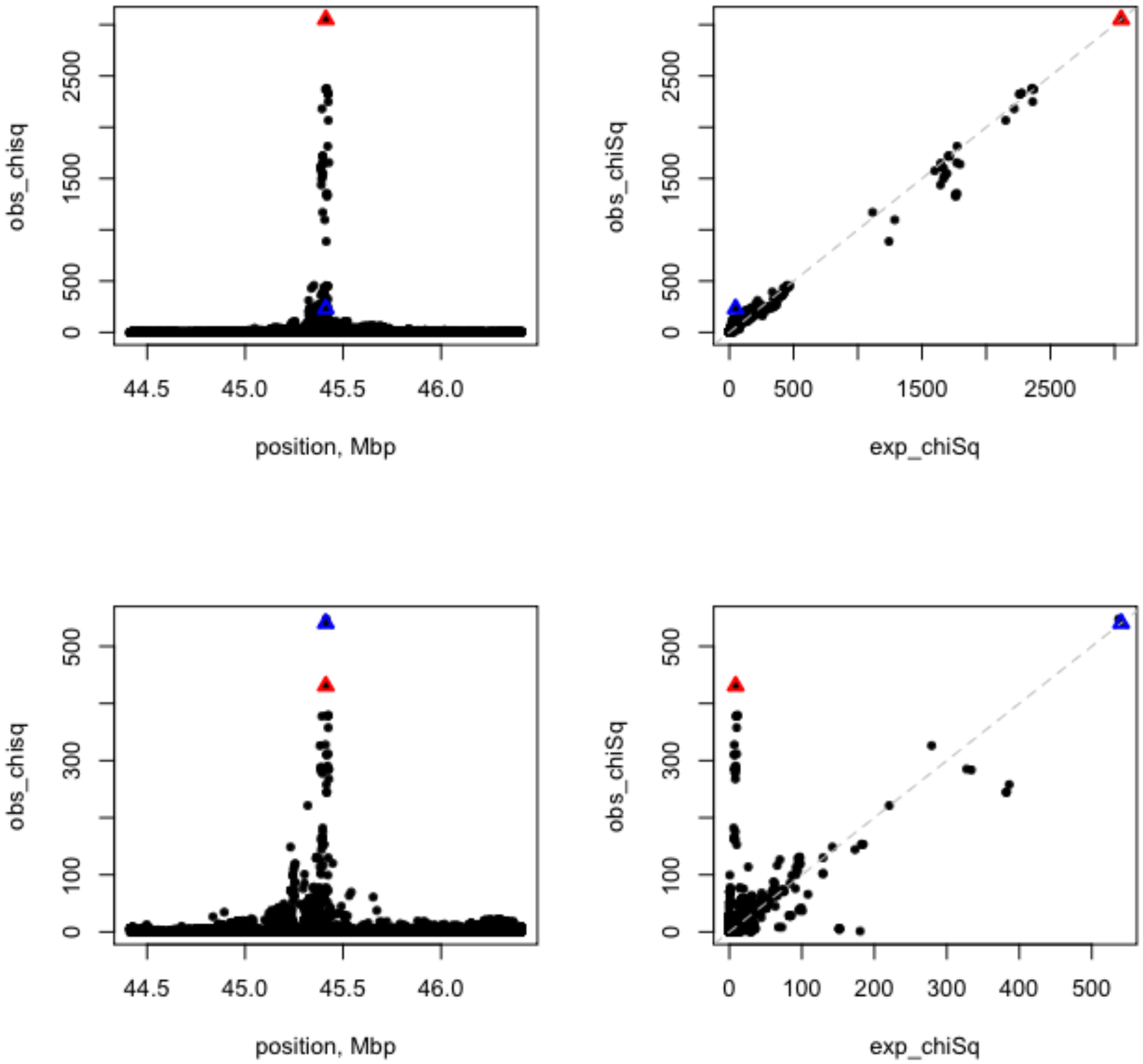

Supplementary Figure S4. Observed and expected chi-square statistics for two traits in the UK Biobank near the APOE locus, using results from Jiang et al. [1]**.**

The two traits were reporting an AD diagnosis in the UK Biobank participants mother (top, phenotype code 20110_10) and taking cholesterol lowering medication (bottom, phenotype code 6153_1). The left plots are typical association plots using the χ^2_1 test statistic on y-axis and position on chromosome 19. On the right is observed χ^2_1 test statistic (y-axis) and the expected χ^2_1 value (x-axis) given the focal variant of either rs427358 (top) or rs7412 (bottom). Expected χ^2_1 values were calculated as the observed χ^2_1 for focal variant multiplied by the linkage disequilibrium (LD, r^2) between the variants, where LD was calculated in the unrelated sample of UK Biobank participants (N = 284,165). In each plot rs427358 is highlighted in red, and rs7412 is highlighted in blue. Alignment of the observed and expected χ^2_1 values for mother's AD diagnosis supports a single causal variant (i.e. all points lie approximately on the dashed y = x line), while there are potentially multiple causal variants evident for cholesterol medication use.

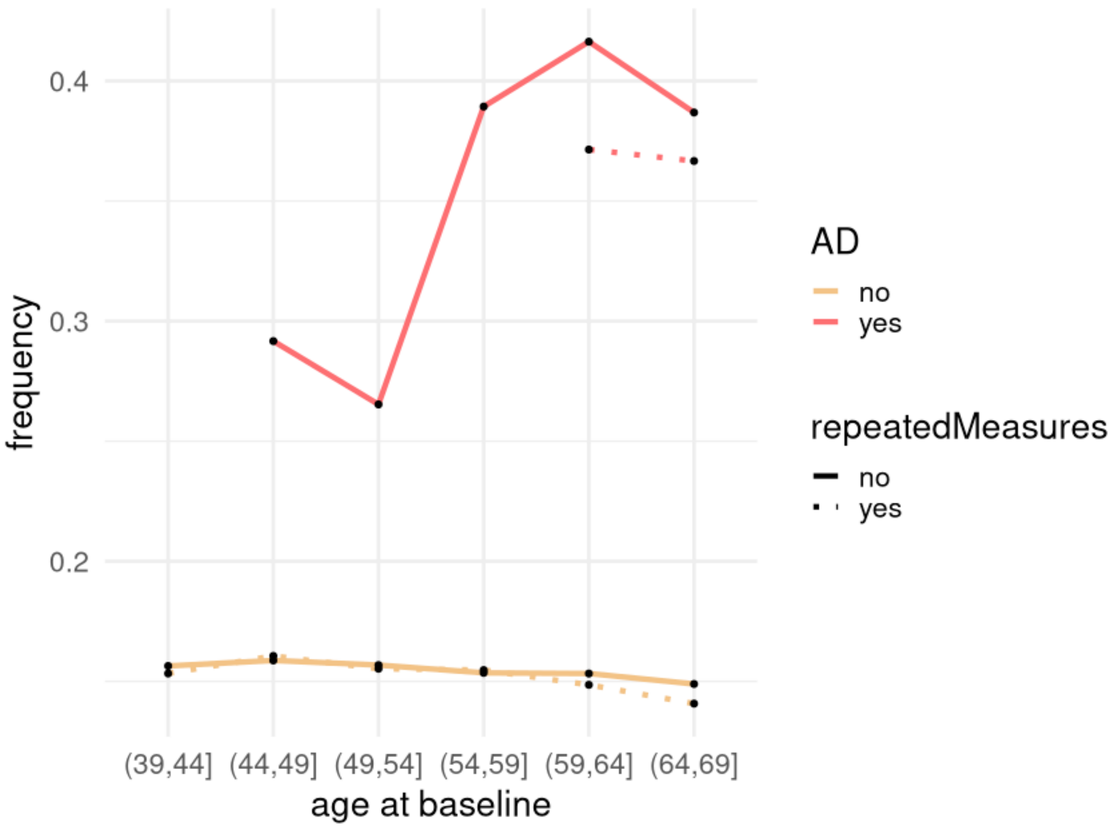

Supplementary Figure S5. Potential recall bias for individuals with repeated assessments in the UK Biobank.

Shown is the frequency of the rs429358-C allele in UK Biobank individuals with only a baseline (single) assessment visit or repeat assessments, and in individuals with and without a diagnosis of Alzheimer's disease (AD, yes = Alzheimer's diagnosis). Only age classes with > 10 individuals are shown. It is evident in older individuals at baseline were more likely to return for repeat assessments if they were not carriers of the rs429358-C allele (i.e. the allele tracking the APOE-$\mathcal{E}4$ AD risk locus). There was a minimum of 4,743 and 27,245 individuals in each age group for the repeat assessment and baseline only age groups, respectively, for those without an AD-diagnosis. Exact numbers per age for all groups can be found in the 'APOE.html' file contained in the Source data file accompanying this manuscript.

Supplementary Table S1. Summary of the phenotypic data from the UK Biobank. Shown above are the fields extracted for the analysis, and below the number of records (after quality control) for each trait. Also shown is the raw correlation (r) between the two repeated measures.

trait	Unit	UK Biobank identifier
height	cm	f.50.0.0, f.50.1.0, f.50.2.0, f.50.3.0
body mass index	kg/cm^2	-
weight	kg	f.21002.0.0, f.21002.1.0, f.21002.2.0, f.21002.3.0
body fat percentage	%	f.23099.0.0, f.23099.1.0, f.23099.2.0, f.23099.3.0
waist circumference	cm	f.48.0.0, f.48.1.0, f.48.2.0, f.48.3.0
hip circumference	cm	f.49.0.0, f.49.1.0, f.49.2.0, f.49.3.0
waist:hip ratio	-	-
sitting height	cm	f.20015.0.0, f.20015.1.0, f.20015.2.0, f.20015.3.0
sex	-	f.31.0.0
age	years	f.21003.0.0, f.21003.1.0, f.21003.2.0, f.21003.3.0
year of birth	-	f.34.0.0
centre	-	f.54.0.0, f.54.1.0, f.54.2.0, f.54.3.0
date	-	f.53.0.0, f.53.1.0, f.53.2.0, f.53.3.0

trait	Baseline (2006-2010)	1st repeat (2012-2013)	1st imaging (2014+)	2nd imagining (2019+)	r
height	50,072	16,731	30,765	2,576	0.993
body mass index	49,968	16,718	30,678	2,572	0.943
weight	49,999	16,726	30,701	2,572	0.799
body fat percentage	48,514	16,290	29,750	2,474	0.846
waist circumference	50,106	16,743	30,787	2,576	0.915
hip circumference	50,107	16,741	30,790	2,576	0.940
waist:hip ratio	50,101	16,738	30,787	2,576	0.917
sitting height	49,649	16,382	30,700	2,567	0.940

Supplementary Table S2. Linear model coefficients for the rate of trait-change with age for height, sitting height, BMI and weight.

	Intercept		Linear		Quadratic	
	Females	Males	Females	Males	Females	Males
Trait mean						
Height	163.1 (0.04)	176.2 (0.04)	-0.166 (0.005)	-0.151 (0.005)		
Sitting height	86.8 (0.02)	92.7 (0.03)	-0.127 (0.003)	-0.115 (0.003)		-0.002 (0.0002)
BMI	26.3 (0.03)	27.3 (0.03)	0.011 (0.004)	-0.017 (0.004)		-0.002 (0.0003)
Weight	70.0 (0.09)	84.8 (0.11)	-0.114 (0.011)	-0.199 (0.011)		-0.007 (0.0010)
Trait change						
Height	-0.096 (0.001)	-0.086 (0.001)	-0.005 (0.0001)	-0.004 (0.0001)		
Sitting height	-0.120 (0.002)	-0.109 (0.002)	-0.005 (0.0002)	-0.004 (0.0002)		
BMI	-0.002 (0.002)	-0.002 (0.002)	-0.004 (0.0002)	-0.002 (0.0002)	0.0002 (0.00003)	0.00002 (0.00003)
Weight	-0.093 (0.005)	-0.093 (0.005)	-0.016 (0.0007)	-0.010 (0.0007)	0.0006 (0.00008)	0.00007 (0.00007)

Supplementary Table S3. SNP-based heritability (h^2) and genetic correlation (r_g) estimates between different age subsets of the UK Biobank, 40-49 years (subset 1, N = 79,680), 50-54 years (subset 2, N = 52,744), 55-59 years (subset 3, N = 63,785), 60-64 years (subset 4, N = 89,539) and 65-69 years (subset 5, N = 71,438) where age is the age of measurement. Shown are estimates from a series of bivariate Haseman-Elston regressions where unadjusted P values from chi-squared tests (with 1 df) indicate a significant difference of r_g from 1.

		Height			Sitting height			BMI			Weight		
	set	est.	s.e.	P	est.	s.e.	P	est	s.e.	P	est	s.e.	P
h^2	1	0.613	0.013		0.448	0.011		0.273	0.010		0.288	0.010	
	2	0.612	0.016		0.425	0.014		0.257	0.012		0.277	0.012	
	3	0.607	0.014		0.419	0.012		0.273	0.011		0.294	0.011	
	4	0.606	0.012		0.399	0.009		0.241	0.007		0.263	0.007	
	5	0.588	0.013		0.393	0.011		0.237	0.009		0.252	0.009	
r_g	1,2	1.003	0.011	0.799	0.997	0.015	0.831	1.011	0.024	0.650	1.016	0.022	0.483
	1,3	1.001	0.010	0.954	0.988	0.013	0.355	0.956	0.021	0.038	0.960	0.020	0.048
	1,4	0.998	0.008	0.790	0.982	0.012	0.144	0.958	0.021	0.052	0.962	0.020	0.062
	1,5	0.998	0.010	0.807	0.971	0.013	0.031	0.926	0.023	0.001	0.919	0.023	3.4×10^{-4}
	2,3	1.009	0.012	0.425	0.999	0.017	0.958	1.021	0.027	0.432	1.011	0.025	0.661
	2,4	1.000	0.010	0.981	0.993	0.015	0.657	0.978	0.024	0.355	0.971	0.022	0.189
	2,5	0.987	0.011	0.244	0.965	0.016	0.030	0.974	0.027	0.338	0.962	0.025	0.127
	3,4	1.009	0.009	0.324	1.004	0.013	0.773	0.951	0.022	0.028	0.962	0.020	0.056
	3,5	1.013	0.010	0.204	0.995	0.015	0.725	0.965	0.025	0.166	0.958	0.024	0.076
	4,5	0.998	0.009	0.858	0.993	0.013	0.620	0.997	0.022	0.887	1.007	0.020	0.742

Supplementary Table S4. Logistic regression coefficients for the phenotypic association between age-corrected trait change in height, sitting height, weight and body mass index (BMI) and all-cause mortality. Unadjusted P values are from chi-squared tests (with 1 df) and test for a significant difference from zero.

Trait	linear	s.e.	P	quadratic	s.e.	P
height	-0.217	0.031	1.9×10^{-14}	0.037	0.007	3.7×10^{-7}
sittingH	-0.212	0.030	2.3×10^{-12}	0.018	0.007	0.010
weight	-0.002	0.025	0.933	0.044	0.006	2.8×10^{-13}
BMI	0.034	0.024	0.153	0.042	0.006	8.2×10^{-13}

Supplementary Table S5. Observed and expected frequency of the APOE alleles based on rs429358 and rs7412. The common APOE alleles are $\mathcal{E}2$, $\mathcal{E}3$ and $\mathcal{E}4$, and there are few observations of the rare $\mathcal{E}3$r allele. Frequencies were calculated in the UK Biobank unrelated sample (N = 284,165), and the definition of the APOE alleles follow Seripa et al.[2].

**Haplotype**	**Allele**	**Isoform**	**Observed frequency**	**Expected frequency***
CT	$\mathcal{E}3r$	Arg^{112}-Cys^{158}	0.0001	0.0125
TT	$\mathcal{E}2$	Cys^{112}-Cys^{158}	0.0804	0.0680
CC	$\mathcal{E}4$	Arg^{112}-Arg^{158}	0.1554	0.1430
TC	$\mathcal{E}3$	Cys^{112}-Arg^{158}	0.7641	0.7765

*frequency rs429358 C allele = 0.1547 and frequency rs7412 C allele = 0.9195; expected haplotype frequency assumes independence between the SNP.

Supplementary Table S6. Frequency of disease diagnoses in individuals with 1 (baseline only) observation (N = 284,165), or repeated observations (N = 50,117) for osteopathic fracture, lumbar spine fracture, coronary artery disease or Alzheimer's disease. Also shown is the frequency of all-cause mortality.

	1 observation	2+ observations
major osteopathic fracture	8,858 (3.12%)	848 (1.69%)
lumbar spine fracture	469 (0.16%)	64 (0.13%)
coronary artery disease	11,452 (4.03%)	1,440 (2.88%)
Alzheimer's disease	1,789 (0.63%)	92 (0.18%)
all-cause mortality	23,211 (8.17%)	1,198 (2.39%)

Supplementary Table S7. Logistic regression coefficients for outcomes of all-cause mortality, major osteopathic fracture (MOF), lumbar spine fracture (LSF), coronary artery disease (CAD) or Alzheimer's disease (AD) in the independent subset of the UK Biobank (N = 284,165) using polygenic scores (PGS) for rate of height, sitting height (sittingH), body mass index (BMI) and weight change. Unadjusted P values are from chi-squared tests (with 1 df) and test for a significant difference from zero.

Outcome	Trait	PGS	s.e.	P
all-cause mortality	height	-0.005	0.007	0.484
	sittingH	-0.006	0.007	0.398
	weight	0.001	0.007	0.859
	BMI	0.003	0.007	0.682
MOF	height	-0.026	0.011	0.015
	sittingH	-0.007	0.011	0.495
	weight	-0.011	0.011	0.292
	BMI	-0.006	0.011	0.571
LSF	height	-0.072	0.046	0.118
	sittingH	-0.071	0.046	0.123
	weight	-0.005	0.046	0.912
	BMI	0.009	0.046	0.841
CAD	height	0.005	0.010	0.586
	sittingH	0.006	0.010	0.485
	weight	0.012	0.010	0.194
	BMI	0.015	0.010	0.127
AD	height	-0.034	0.024	0.150
	sittingH	-0.026	0.024	0.268
	weight	-0.024	0.024	0.318
	BMI	-0.016	0.024	0.505

SUPPLEMENTARY NOTE 1

Reduction in phenotypic variance due to the analysis of the mean from *n* repeated measurements

The variance of the mean of *n* measurements of the same trait (i.e. when the genetic correlation between measurements is 1) is:

$$\left[\frac{1 + (n - 1)r}{n}\right]\sigma_P^2$$

where n is the number of measurements, r is the intra-class correlation or repeatability of the trait, and σ_P^2 is the phenotypic variance (calculated using a single measurement)[3]. Since $n = 2$ and assuming $r = 0.9$; then the observed variance of the mean is $0.95\sigma_P^2$. The heritability estimated from the mean of 2 measurements is thus increased by a factor of $1/0.95 = 1.05$ compared to an estimate from a single measurement.

SUPPLEMENTARY NOTE 2

Estimating cumulative trait change via integration

Integration of equations relating rate of trait change with age (coefficients in Supplementary Table S2) to estimate the cumulative trait change with age.

Trait	Sex	Equations
Height	Females	$= \int -0.096 - 0.005(age - 59.0)$. dAge<br>$= -0.096\ age - 0.0025\ age^2$
	Males	$= \int -0.086 - 0.004(age - 60.3)$. dAge<br>$= -0.086\ age - 0.002\ age^2$
Sitting height	Females	$= \int -0.120 - 0.005(age - 59.0)$. dAge<br>$= -0.120\ age - 0.0025\ age^2$
	Males	$= \int -0.109 - 0.004(age - 60.3)$. dAge<br>$= -0.109\ age - 0.002\ age^2$
BMI	Females	$= \int -0.002 - 0.004(age - 59.0) - 0.0001(age - 59.0)^2$. dAge<br>$= -0.002\ age - 0.002\ age^2 - 0.00007 age^3$
	Males	$= \int -0.002 - 0.004(age - 60.3) - 0.0001(age - 60.3)^2$. dAge<br>$= -0.002\ age - 0.001\ age^2 - 0.000005\ age^3$
Weight	Females	$= \int -0.093 - 0.016(age - 59.0) - 0.0006(age - 59.0)^2$. dAge<br>$=-0.093\ age - 0.008\ age^2 - 0.0002\ age^3$
	Males	$= \int -0.093 - 0.010(age - 60.3) - 0.00007(age - 60.3)^2$. dAge<br>$=-0.093\ age - 0.005\ age^2 - 0.00003\ age^3$

SUPPLEMENTARY NOTE 3

A 2-stage random regression analysis

3.1 Theory:

| calculating variance components & genetic parameters (with s.e.) on the original scale from variance components estimated using a bivariate GREML analysis of the intercept and slope

To calculate the variance components (e.g. genetic or phenotypic variance) and genetic parameters (e.g. heritability) we need to calculate the genetic and residual variance at each age, and use their associated sampling variances to calculate the standard errors. These can be calculated from the estimated genetic and residual variance-covariance matrix for the mean & slope, plus the sampling variance-covariance matrix between all the terms.

The genetic or residual variance can be calculated as a function of the age (x) as:

$$f(x) = a + 2bx + cx^2$$

where the $f(x)$ is the genetic or residual variance at age x, a is the genetic or residual variance estimate for the mean, c is the genetic or residual variance estimate for the slope and b is the genetic or residual variance estimate of covariance between the mean and the slope.

The sampling variance of $f(x)$ can be computed using the variance properties of linear functions and assuming that, at each age, x is a constant. For example,

$$\begin{aligned} var[f(x)] &= var(a) + var(2bx) + var(cx^2) + 2cov(a, 2bx) + 2cov(a, cx^2) + 2cov(b, cx^2) \\ &= var(a) + 4x^2. var(b) + x^4. var(c) + 4x. cov(a, b) + 2x^2 cov(a, c) + 2x^2. cov(b, c) \end{aligned}$$

In a similar manner, the sampling covariance between the genetic and residual variance can be calculated using the covariance properties of linear functions. Thus,

$$\begin{aligned} cov[f_g(x), f_e(x)] &= cov[a_g + 2b_g x + c_g x^2, a_e + 2b_e x + c_e x^2] \\ &= cov(a_g, a_e) + cov(a_g, 2b_e x) + cov(a_g, c_e x^2) + \ldots \\ &= cov(a_g, a_e) + 2x. cov(a_g, b_e) + x^2. cov(a_g, c_e) + \ldots \end{aligned}$$

The sampling variance of the heritability estimate (i.e. a ratio) was approximated using the delta method, following Lynch and Walsh[4] and Gilmore et al. [5]. That is,

$$var\left(\frac{\sigma_n^2}{\sigma_d^2}\right) = \left(\frac{\sigma_n^2}{\sigma_d^2}\right)^2 \left[\frac{var(\sigma_n^2)}{[\sigma_n^2]^2} + \frac{var(\sigma_d^2)}{[\sigma_d^2]^2} - \frac{2. cov(\sigma_n^2, \sigma_d^2)}{\sigma_n^2 \sigma_d^2}\right]$$

where n and d are the numerator and denominator respectively. Thus for the calculation of heritability, the numerator is the estimate of the genetic variance ($\hat{\sigma}_a^2$) and the denominator

the estimate of the phenotypic variance ($\hat{\sigma}_P^2$). Terms were obtained by writing out the variances and covariance in terms of the linear function, noting that $cov(\sigma_a^2, \sigma_P^2) = cov(\hat{\sigma}_a^2, \hat{\sigma}_a^2 + \hat{\sigma}_e^2) = var(\hat{\sigma}_a^2) + cov(\hat{\sigma}_a^2, \hat{\sigma}_e^2)$.

A function in R was written as follows:

```
estimateh2 <- function(x,vcov1,vcov2,samp) {
    estG <- vcov1[1,1] + 2*vcov1[2,1]*x + vcov1[2,2]*x^2
    estE <- vcov2[1,1] + 2*vcov2[2,1]*x + vcov2[2,2]*x^2
    varG <- samp[1,1] + 4*x^2*samp[2,2] + x^4*samp[3,3] +
                4*x*samp[2,1] + 2*x^2*samp[3,1] + 4*x^3*samp[3,2]
    varE <- samp[4,4] + 4*x^2*samp[5,5] + x^4*samp[6,6] +
                 4*x*samp[5,4] + 2*x^2*samp[6,4] + 4*x^3*samp[6,5]
    covGE<- samp[4,1] + 2*x*samp[5,1] + x^2*samp[6,1] +
      2*x*samp[4,2] + 4*x^2*samp[5,2] + 2*x^3*samp[6,2] +
      x^2*samp[4,3] + 2*x^3*samp[5,3] + x^4*samp[6,3]
    estP <- estG + estE
    varP <- varG + varE + 2*covGE
    h2 <- estG / estP
    varh2<- h2^2 * (varG/estG^2 + varP/estP^2 - 2*(varG+covGE)/(estG*estP))
    return(cbind(c(estG,estE,estP,h2),sqrt(c(varG,varE,varP,varh2))))
}
```

Calculation of the genetic correlation between two ages (x_1 and x_2) followed a similar approach to the calculation of the genetic variance and heritability estimates above. The estimate of the genetic covariance between x_1 and x_2 is given by:

$$f(x_1, x_2) = a + b(x_1 + x_2) + x_1 x_2 c$$

where a, b and c are as before (the genetic variance of the mean, slope and covariance between them).

The genetic correlation (r_g) at between age x_1 and x_2 is given by:

$$r_g = \frac{\sigma_{x_1 x_2}}{\sigma_{x_1}\sigma_{x_2}}$$

and its sampling variance by:

$$var(r_g) = r_g^2 \left[\frac{var(\sigma_{x_1}^2)}{4[\sigma_{x_1}^2]^2} + \frac{var(\sigma_{x_2}^2)}{4[\sigma_{x_2}^2]^2} + \frac{var(\sigma_{x_1 x_2})}{[\sigma_{x_1 x_2}]^2} + \frac{2.\,cov(\sigma_{x_1}^2, \sigma_{x_2}^2)}{4\sigma_{x_1}^2 \sigma_{x_2}^2} - \frac{2.\,cov(\sigma_{x_1}^2, \sigma_{x_1 x_2})}{2\sigma_{x_1}^2 \sigma_{x_1 x_2}} - \frac{2.\,cov(\sigma_{x_2}^2, \sigma_{x_1 x_2})}{2\sigma_{x_2}^2 \sigma_{x_1 x_2}} \right]$$

The function is written in R as:

```
rg <- function(x1,x2,vcov,samp) {
    est1 <- vcov[1,1] + 2*vcov[2,1]*x1 + vcov[2,2]*x1^2
    est2 <- vcov[1,1] + 2*vcov[2,1]*x2 + vcov[2,2]*x2^2
```

```
    est12 <- vcov[1,1] + vcov[2,1]*(x1+x2) + vcov[2,2]*x1*x2
    rg = est12 / sqrt(est1*est2)
    var1 <- samp[1,1] + 4*x1^2*samp[2,2] + x1^4*samp[3,3] +
        4*x1*samp[2,1] + 2*x1^2*samp[3,1] + 4*x1^3*samp[3,2]
    var2 <- samp[1,1] + 4*x2^2*samp[2,2] + x2^4*samp[3,3] +
        4*x2*samp[2,1] + 2*x2^2*samp[3,1] + 4*x2^3*samp[3,2]
    var12<- samp[1,1] + (x1+x2)^2*samp[2,2] + x1^2*x2^2*samp[3,3] +
        2*(x1+x2)*samp[2,1] + 2*x1*x2*samp[3,1] + 2*x1*x2*(x1+x2)*samp[3,2]
    cov1_2 <- samp[1,1] + 4*x1*x2*samp[2,2] + x1^2*x2^2*samp[3,3] +
        2*(x1+x2)*samp[2,1] + (x1^2+x2^2)*samp[3,1] + 2*x1*x2*(x1+x2)*samp[3,2
]
    cov1_12 <- samp[1,1] + 2*x1*(x1+x2)*samp[2,2] + x1^3*x2*samp[3,3] +
        (3*x1+x2)*samp[2,1] + x1*(x1+x2)*samp[3,1] + x1^2*(x1+3*x2)*samp[3,2]
    cov2_12 <- samp[1,1] + 2*x2*(x1+x2)*samp[2,2] + x2^3*x1*samp[3,3] +
        (3*x2+x1)*samp[2,1] + x2*(x1+x2)*samp[3,1] + x2^2*(x2+3*x1)*samp[3,2]
    var_rg <- rg^2 * (var1/(4*est1^2) + var2/(4*est2^2) + var12/est12^2 +
        2*cov1_2/(4*est1*est2) - 2*cov1_12/(2*est1*est12) - 2*cov2_12/(2*est12
*est2))
    return(cbind(rg,sqrt(var_rg)))
}
```

3.2 Application:

| *using estimates from a bivariate GREML analysis as a 2-stage RR*

We need to read in the data from the bivariate GREML analysis in GCTA. Note here that the order of the terms is changed from the GCTA output for ease of handling.

```
t=0 ; variances=matrix(NA,nrow=6, ncol=4) ; sampling=matrix(NA,nrow=6*4,ncol=6
)
traits = c("height","weight","BMI","sit")
for (trait in traits) {
  t=t+1
  skip=60 ; if(trait=="sit") skip=61
  tmp = read.table(paste0(dir,trait,"_ageCorrected_bivar.log"), skip=skip, fil
l=T, nrow=6)[,2]
  variances[,t] = tmp[c(1,3,2,4,6,5)]

  skip=73 ; if(trait=="sit") skip=74
  tmp = as.matrix(read.table(paste0(dir,trait,"_ageCorrected_bivar.log"), fill
=T,skip=skip,nrow=6))
  tmp = as.vector(tmp)
  a = c(1,3,2,4,6,5) ; a=rep(a,6)
  b=NULL ; for(i in 1:6) b=c(b,rep(a[i],6))
  for (i in 1:length(tmp)) sampling[((t-1)*6)+a[i],b[i]]=tmp[i] #reorder
}
colnames(variances) = traits
rownames(variances) = c("G_mean","G_cov","G_slope","E_mean","E_cov","E_slope")
round(variances,3)

##          height  weight    BMI    sit
## G_mean   19.718  46.075  4.494  4.661
## G_cov    -0.012   0.086  0.018 -0.006
## G_slope   0.000   0.020  0.003  0.001
## E_mean   17.877 116.186 13.204  5.643
```

```
## E_cov    0.011   0.467  0.081  0.001
## E_slope  0.027   0.611  0.076  0.067
```

The second step is to use the functions written above to calculate the variance components.

```
relAge = -15:15   # relative ages, relative to a mean of approx. 60 years
table=NULL
for(i in 1:4) {
  K = matrix(variances[c(1,2,2,3),i],nrow=2)
  E = matrix(variances[c(4,5,5,6),i],nrow=2)
  t = (i-1)*6
  sampling1 = sampling[(t+1):(t+6),1:6]
  for (j in relAge) table=rbind(table,estimateh2(j,K,E,sampling1))
}
table = data.frame(trait=rep(traits,each=length(relAge)*4),
           relAge = rep(rep(relAge,each=4),4) ,
           type=rep(c("G","E","P","h2"),length(relAge)*4),
           table)
names(table)[4:5] = c("estimate","se")
table$upperCI = table$estimate + 1.96*table$se
table$lowerCI = table$estimate - 1.96*table$se
table$approxAge = table$relAge + 60
```

3.3 Application:

3.3.1 Variance components across age

Note: with 95% confidence intervals.

```
  table1 = table[table$type!="h2",]
  ggplot(table1,aes(x=approxAge,y=estimate,col=type)) +
     geom_point(pch=4) +
     scale_color_manual(values=c("#999999", "#E69F00", "#56B4E9")) +
     geom_errorbar(aes(ymin=lowerCI, ymax=upperCI,col=type), width=.2) +
     facet_wrap(trait~., scales="free_y") +
     ylab("trait variance") + xlab("age") +
     theme_grey(base_size = 20)
```

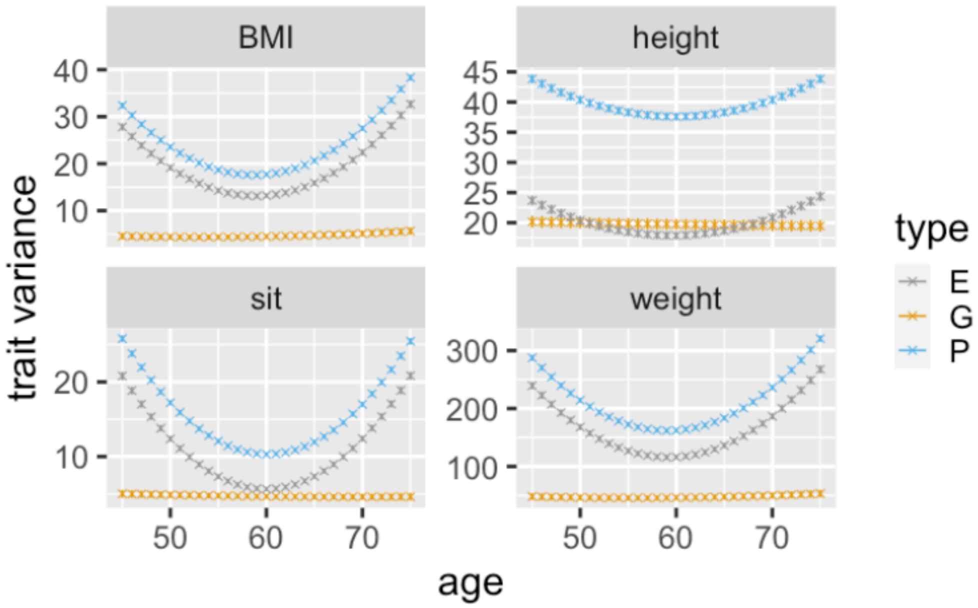

3.3.2 Heritability as a function of age

```
table1 = table[table$type=="h2",]
ggplot(table1,aes(x=approxAge,y=estimate)) +
   geom_point() + ylim(c(0,0.6)) +
   geom_errorbar(aes(ymin=lowerCI, ymax=upperCI), width=.2) +
   facet_wrap(.~trait, scales="free") +
   theme_grey(base_size = 20)
```

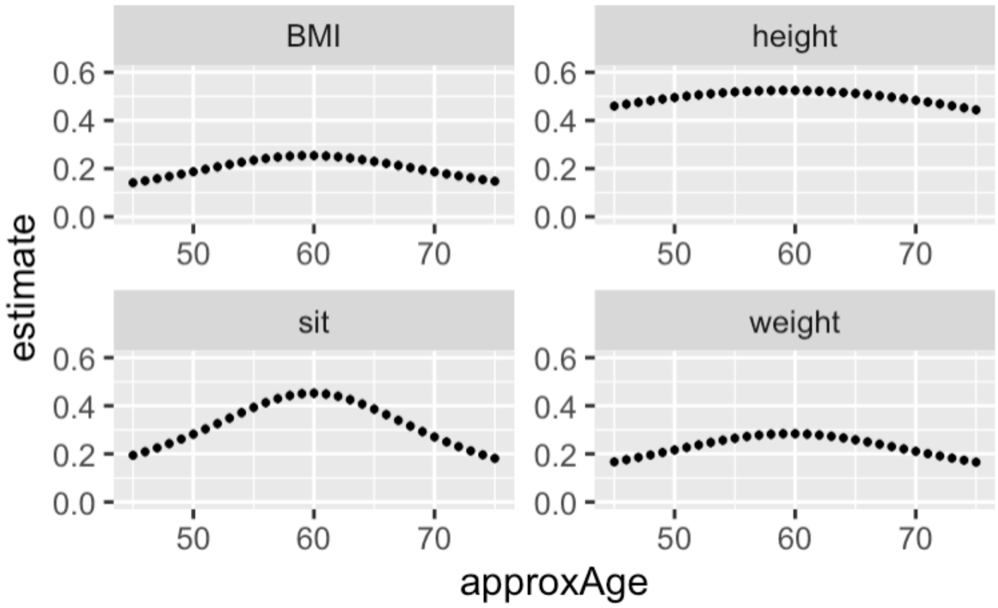

3.3.3 Genetic correlations as a function of age

```
table=NULL ; tmp=NULL
for (i in 1:4) {
 t = (i-1)*6
 K = matrix(variances[c(1,2,2,3),i],nrow=2)
 genetic = sampling[(t+1):(t+3),1:3]
```

```
    tmp=rbind(tmp,cbind(relAge,-10,rg(relAge,-10,K,genetic)))
    tmp=rbind(tmp,cbind(relAge,0,rg(relAge,0,K,genetic)))
    tmp=rbind(tmp,cbind(relAge,10,rg(relAge,10,K,genetic)))
  }
  table = data.frame(trait=rep(traits,each=length(relAge)*3),tmp)
  names(table)=c("trait","x1","x2","rg","se")
  table$upperCI = table$rg + 1.96*table$se
  table$lowerCI = table$rg - 1.96*table$se
  table$x1 = table$x1 + 60
  table$x2 = table$x2 + 60

  ggplot(table,aes(x=x1,y=rg)) +
    geom_point() +
    ylim(c(min(table$lowerCI),max(table$upperCI))) +
    geom_errorbar(aes(ymin=lowerCI, ymax=upperCI), width=.2) +
    facet_grid(trait~x2) +
    theme_grey() +
    xlab(expression(age~(x[2]))) + ylab(expression(genetic~correlation~(r[g])
))
```

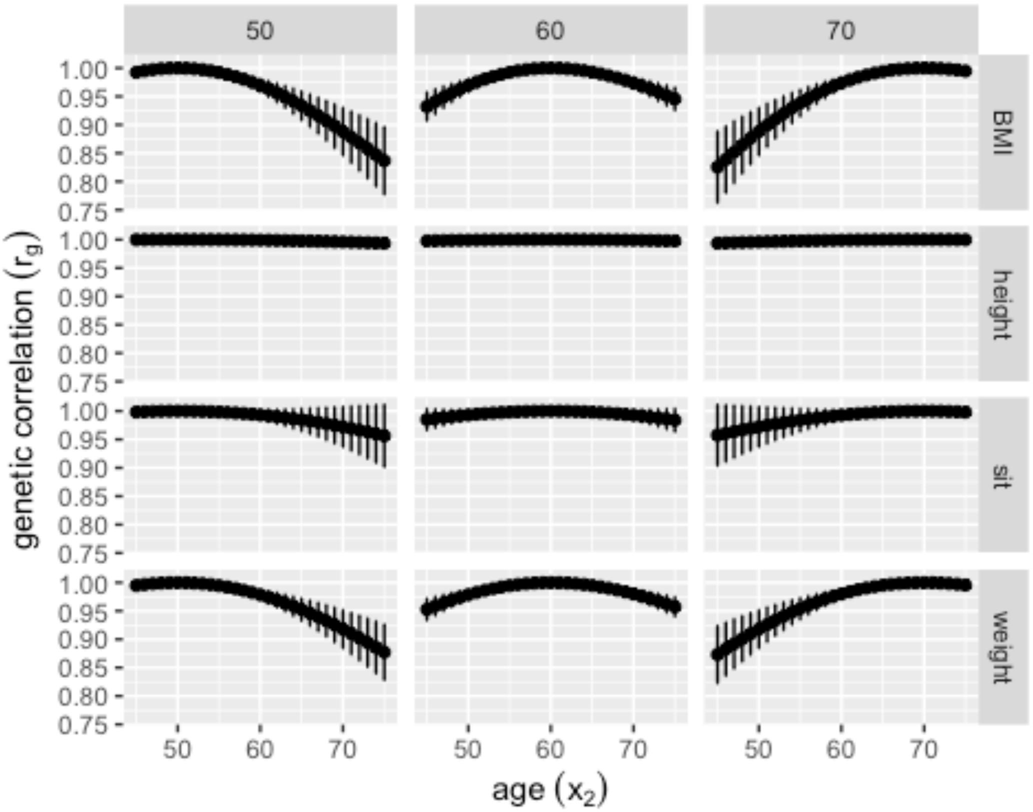

3.3.4 Test of rg < 1 between young and old age for weight

```
   i = 2 ; traits[i]

## [1] "weight"

   t = (i-1)*6
   K = matrix(variances[c(1,2,2,3),i],nrow=2) ; K

##            [,1]     [,2]
## [1,] 46.075183 0.085515
## [2,]  0.085515 0.019839
```

```
    genetic = sampling[(t+1):(t+3),1:3]
    genetic

##              [,1]         [,2]         [,3]
## [1,] 2.112016e+00 6.186288e-03 1.825517e-05
## [2,] 6.186288e-03 3.179971e-03 1.852516e-05
## [3,] 1.825517e-05 1.852516e-05 1.811350e-05

    Rage1 = 50 - 60 # i.e. evaluate 50 minus avg age
    Rage2 = 70 - 60 # i.e. evaluate 70 minus avg age
    est1 = rg(Rage1,Rage2,K,genetic)
    est1

##             rg
## [1,] 0.9180206 0.0171385

    chisq1 = ((1 - est1[1])/est1[2])^2
    pchisq(chisq1, 1, lower.tail = FALSE)

## [1] 1.724014e-06
```

SUPPLEMENTARY NOTE 4

Expected power of rate-change PRS to predict disease

If *x* and *y* are quantitative traits, i.e. where *x* is the rate-change trait and *y* is liability to disease on the underlying scale, and they are assumed to be distributed N(0,1); then the correlation ($\rho_{y,\hat{x}}$) between *y* and the polygenic prediction of *x* is given by:

$$\rho_{y,\hat{x}} = r_g h_y R_x / h_x$$

where r_g is the genetic correlation between traits, h_y and h_x are the square-root of the heritability of traits *y* and *x*, and R_x is the square-root of the phenotypic variance in *x* explained by the PGS. Using this relationship we can approximate the magnitude of $\rho_{y,\hat{x}}$ by assuming the genetic correlation between traits is 0.5, the heritability of the rate-change trait is 0.02, the heritability of the disease trait is 0.5, and the phenotypic variance explained by the PGS is 0.0005, then

$$\rho_{y,\hat{x}} = 0.5[(0.5x0.0005)/0.02]^{1/2} \approx 0.05$$

So we expect the correlation between disease liability and the rate-change PGS to be about 0.05. The next question is do we have enough power to detect a correlation of this magnitude with a sample size of approximately 280,000 individuals?

We use simulations in R to determine the power of the available sample size (N = 280,000). Briefly, we simulated a PGS ($\hat{x}$) with a standard normal distribution for 280K individuals. Then we used a logistic regression model for a given PGS effect (β_1) and prevalence to determine the liability to disease, i.e.

$$\Pr\{y = 1|\hat{x}\} = \frac{1}{1 + \exp(-\hat{y})}$$

where $\hat{y} = \beta_0 + \beta_1 \hat{x}$. A binomial distribution was used to sample phenotypes from the liability and the glm() function in R was used for the logistic regression using a binomial link function. To determine power we calculated the proportion of replicates with a significant regression co-efficient (β_1, P < 0.05) from 100 replicates.

The power to detect a correlation of a given magnitude is independent of population prevalence of the disease, and we have sufficient power (>80%) to detect absolute correlations greater than ~0.005 (Supplementary Figure S6). However, using logistic regression we also demonstrate that the correlation captures both the disease incidence and PGS effect size. Thus to achieve a correlation of the expected magnitude (0.005), diseases with low prevalence require the PGS to have a very large effect (β_1) size. For diseases with a prevalence > 3% we have > 80% power to detect a PGS with an absolute effect of about 0.03 or an odds-ratio of 1.03.

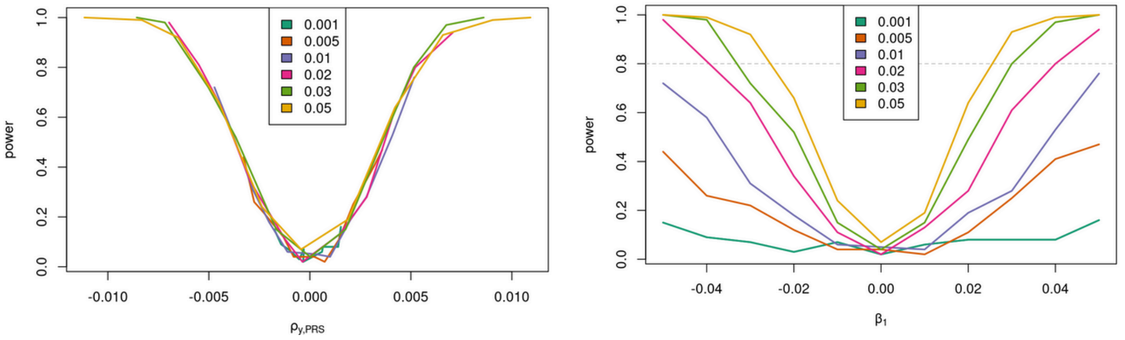

Supplementary Figure S6. Power to detect a given correlation between a disease trait *y* and a PGS of a second trait *x* (left), and a given effect size of the PGS (right).
Coloured lines show the incidence of disease, from 0.1% to 5%.

SUPPLEMENTARY NOTE 5

Case-control GWAS for repeated measures

We tested 8,544,904 imputed sequence variants (MAF > 0.01, missingness < 0.05) from the UK Biobank for an association with having only baseline measurements or repeated measurements using a case-control design (i.e. where cases were those individuals with repeated measures). Associations used the --fastGWA-lr option in GCTA (v1.93.2 beta) and we fitted 25 principal components[6] as covariates. Even with 8.5 million (independent) tests we do not expect any variants to reach genome wide significance (8,544,904 x 5e-8 = 0.4). However, we identified 11 genome-wide significant variants ($P < 5x10^{-8}$, Supplementary Table S9) in 5 genomic regions (defined by variants within 150 kb).

Supplementary Table S9. Variants associated with having only a single baseline measurement or repeated measurements. Shown are the chromosome (CHR) and base pair position (POS), SNP allele name (SNP), the effect allele (A1), the alternate allele (A2), the sample size (N), allele frequency of allele A1 (AF1), the linear regression effect (BETA) with standard error (SE) and chi-squared P value (P, 1 df).

CHR	SNP	POS	A1	A2	N	AF1	BETA	SE	P
6	rs9372625	98344031	G	A	330311	0.617	-0.005	0.001	2.92E-08
7	rs782552781	72857857	TTTC	T	334016	0.525	-0.005	0.001	3.47E-09
7	rs2237279	72861849	T	C	333342	0.525	-0.005	0.001	4.49E-09
7	rs2074754	72891754	C	T	334282	0.525	-0.005	0.001	2.54E-09
7	rs66579735	72944370	C	T	327885	0.534	-0.005	0.001	5.60E-09
7	rs3763432	72974869	C	T	334282	0.597	-0.005	0.001	4.48E-08
7	rs12531884	72981883	A	C	332437	0.597	-0.005	0.001	3.51E-08
8	rs2410678	21049455	C	A	330969	0.817	0.006	0.001	3.58E-08
14	rs138715058	31094436	T	TAGTA	332094	0.720	-0.005	0.001	4.58E-08
18	rs784257	53397199	T	C	330016	0.185	0.006	0.001	1.87E-08
18	rs784256	53398626	G	A	330185	0.186	0.006	0.001	1.08E-08

We browsed the variants in the 'PheWAS' section of the GWAS Atlas (23rd November 2023) to identify other traits associated with the top variant in each region, and report the most significant association from the GWAS Atlas in Supplementary Table S10. Significant variants in our case-control GWAS for repeated measures were associated with traits such as educational attainment, intelligence and BMI (rs9372625), triglyceride cholesterol and height (rs2074754), sleep duration (rs2410678), and walking pace, impedance, mood swings, loneliness and educational attainment (rs784256). No other traits were associated with rs138715058 (P > 5e-8).

Supplementary Table S10. Most significant association for variants in the GWAS Atlas. Shown are the SNP name (SNP) and chromosome (CHR), the trait associated with the SNP (Trait), reported p value (P), the effect allele (EA) increasing the trait, the other allele (non-effect allele (NEA) and the pubmed publication ID (PMID).

CHR	SNP	Trait	P	EA	NEA	PMID
6	rs9372625	Educational attainment	6.7e-42	A	G	30038396
7	rs2074754	Triglycerides cholesterol	1.2e-17	T	C	24097068
8	rs2410678	Sleep duration	7.5e-6	C	A	30804565
18	rs784256	Usual walking pace	9.9e-15	G	A	31427789

We also queried the online fastGWA portal (23rd November 2023)[1,7] and downloaded summary statistics for associations within (only) the UK Biobank. Many of the associations identified in Supplementary Table S10 were repeated or similar, that is associations were for traits such as educational attainment, income, BMI, height and impedance. However, we identified two associations with traits not in the GWAS Atlas. First, rs2410678 was associated with current smoking status, where the C allele was associated with current smoking (beta = -0.0036 s.e. 0.0008 P = 2.5e-6) [1]. Second, the G allele of rs784256 was associated with corneal dystrophy (beta = 1.725 s.e. 0.193 P = 4.2e-19)[7]. There were only 125 cases for this disease in the UK Biobank but the locus has been identified in larger studies targeting Fuchs endothelial corneal dystrophy[8]. Corneal dystrophy is a late onset disease, becoming symptomatic more frequently in females and typically occurs after about 50 years of age.

SUPPLEMENTARY NOTE 6

Influence of scale

A key criticism of studies investigating trait variance is that the effects are scale-dependent, and that there is implicitly a scale on which the variances can be homogenised[9]. However, this argument could be applied to any studies of interactions (e.g., genotype-by-environment interactions) and therefore changes to variance on the observed or measured scale may be important if this is the biologically or medically relevant scale. For example, clinicians are likely to be more interested in fluctuations in a person's actual weight rather than, say, its logarithm. Nevertheless, several of our traits show skewed distributions and so we chose to investigate the impact of skewness on our results using a transformation approximating a Box-Cox transformation.

The Box-Cox transformation is:

$$y(\lambda) = \begin{cases} \dfrac{y^{\lambda} - 1}{\lambda}, \lambda \neq 0 \\ \log(y), \lambda = 0 \end{cases}$$

where the function examines a range or family of transformations, depending on the lambda value chosen (e.g., approximating an inverse, log, square root, etc.). We used the boxcox() function from the MASS library in R[10] to determine the lambda with the maximum log-likelihood. The most appropriate transformation for approximately normally distributed residuals (for each trait) was determined using the single measurement subset with the following model fitted to the untransformed data:

y = mu + sex + yob + batch + age + centre + e

where y was the measured phenotype, mu is the overall mean, sex is the individual's genetic sex (2 levels), yob is year-of-birth as a factor (34 levels), batch is the individuals genotyping chip (106 levels), age is the age at measurement in years (30 levels), centre is the assessment centre where the measurement was taken (22 levels) and e is the residual. For simplicity, we approximated the lambda value to an equivalent simple transformation and used this transformation for the specified trait throughout. For example, the lambda value for BMI was -0.9, and this was approximated to an inverse transformation (BMI^{-1}). We obtained a lambda of 1 for several traits, implying an approximately normal distribution for the residuals. The final transformations were BMI^{-1}, log(weight), log(waist) and hip^{-1}, with the no transformations applied to height, fat percentage and WHR.

Estimates of the SNP-based heritability for the trait-mean were similar following transformation but the estimates of SNP-based heritability for within-person variability were about half of those obtained on untransformed traits (Supplementary Table S11). Estimates of the SNP-based heritability of variability for weight (0.033, s.e. 0.007, $P = 1.5 \times 10^{-6}$) and BMI (0.020, s.e. 0.007, P = 0.002) remained significantly greater than zero. Transformation had a dramatic effect on the estimated genetic correlation (r_g) between the trait-mean and variability, reducing r_g from 0.80 (s.e. 0.05) to 0.49 (s.e. 0.07) for weight, and from 0.87 (s.e. 0.05) to close to zero (-0.20 s.e. 0.09) for BMI.

Supplementary Table S11. SNP-based heritability (h^2_{SNP}) and genetic correlation (r_g) between the mean and absolute deviation of 2 repeated measures for traits following transformation. Genetic correlations are reported only when h^2_{SNP} of the mean and absolute deviation are significantly greater than zero (P < 0.05). Estimates are shown with standard errors (s.e.), and chi-squared tests (with 1 df) were used to calculated unadjusted P values.

Trait	h^2_{SNP}					r_g	s.e.	P
	mean	s.e.	deviation	s.e.	P			
BMI^{-1}	0.261	0.008	0.020	0.007	0.002	-0.202	0.085	0.017
log(weight)	0.292	0.008	0.033	0.007	$1.5x10^{-6}$	0.491	0.069	$1.4x10^{-12}$
log(wc)	0.228	0.008	0.004	0.006	0.568	-	-	-
hc^{-1}	0.252	0.008	0.007	0.006	0.239	-	-	-

The replication of loci discovered using population vQTL effects using within-person variability was weaker but robust when transformed data was used to estimate the effect of loci (Supplementary Figure S7). Analysis of the traits independently showed that the regression slope was (nominally) greater than zero for BMI (0.462 s.e. 0.217, P = 0.046) and weight (0.541 s.e. 0.192; P = 0.017), but not significantly different from zero for waist (0.058, s.e. 0.120, P = 0.641) and hip circumference (0.044, s.e. 0.107, P = 0.689).

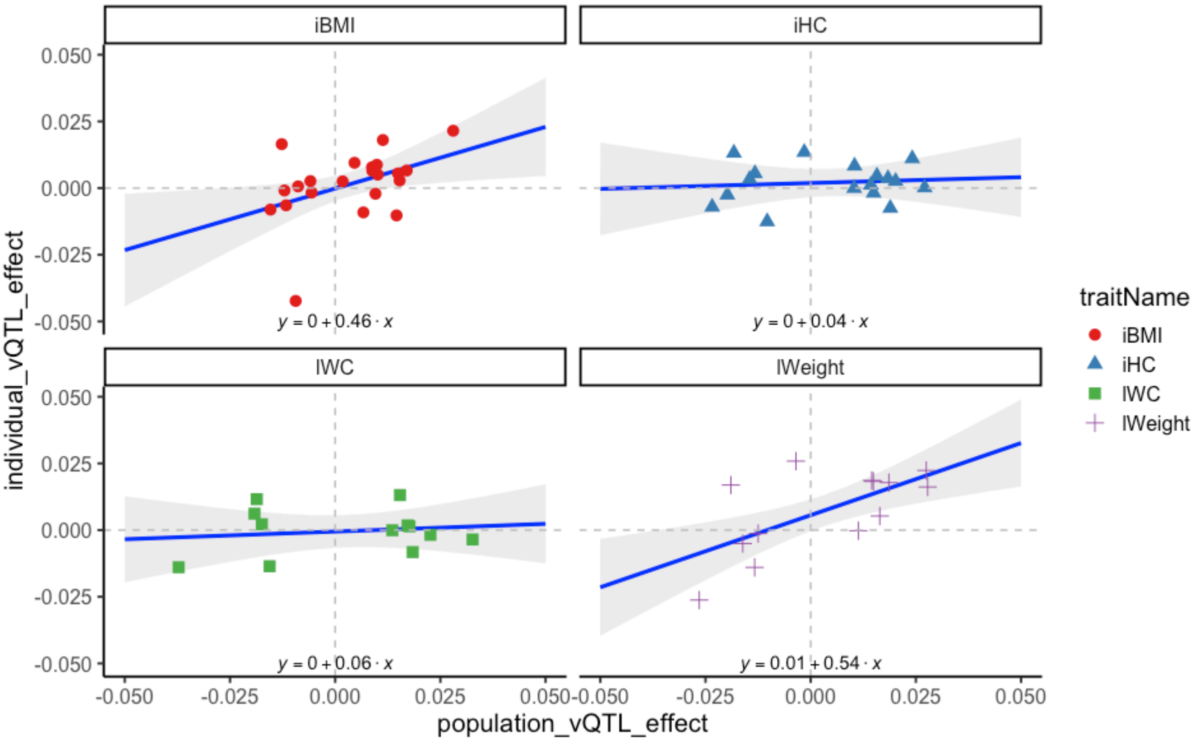

Supplementary Figure S7. Validation of genome-wide significant ($P < 1x10^{-8}$) vQTL loci using transformed traits and within individual variability.

Transformed traits were BMI (BMI^{-1}, iBMI), weight [log(weight), lWeight], waist [log(wc), lWC] and hip (hc^{-1}, iHC) circumference. Note that the loci were discovered using a non-parametric test for heterogeneous error variance and their effects re-estimated using transformed data. The effect is on the variance at the population (x-axis) or individual (y-axis) level.

REFERENCES

1. Jiang, L. *et al.* A resource-efficient tool for mixed model association analysis of large-scale data. *Nature Genetics* **51**, 1749-1755 (2019).
2. Seripa, D. *et al.* The Missing ApoE Allele. *Annals of Human Genetics* **71**, 496-500 (2007).
3. Falconer, D.S. & Mackay, T.F.C. *Introduction to Quantitative Genetics*, (Pearson Education Limited, Edinburgh, UK, 1996).
4. Lynch, M. & Walsh, B. *Genetics and Analysis of Quantitative Traits*, (Sinauer Associates Inc., Sunderland, USA, 1998).
5. Gilmour, A.R., Gogel, B.J., Cullis, B.R. & Thompson, R. *ASReml User Guide Release 2.0*, (VSN International Ltd, Hemel Hempstead, 2006).
6. Kemper, K.E. *et al.* Phenotypic covariance across the entire spectrum of relatedness for 86 billion pairs of individuals. *Nature Communications* (2021).
7. Jiang, L., Zheng, Z., Fang, H. & Yang, J. A generalized linear mixed model association tool for biobank-scale data. *Nat Genet* **53**, 1616-1621 (2021).
8. Afshari, N.A. *et al.* Genome-wide association study identifies three novel loci in Fuchs endothelial corneal dystrophy. *Nature Communications* **8**, 14898 (2017).
9. Sun, X., Elston, R.C., Morris, N. & Zhu, X. What is the significance of difference in phenotypic variability across SNP genotypes? *American Journal of Human Genetics* **93**, 390-397 (2013).
10. Venables, W.N. & Ripley, B.D. *Modern applied statistics with S-Plus*, (Springer-Verlag, New York, 1994).
