# Supplementary material for: Genetic influence on within-person longitudinal change in anthropometric traits in the UK Biobank
Source: Nat Commun. 2024 May 6;15:3776. doi: 10.1038/s41467-024-47802-7 (PMC11074304; doi:10.1038/s41467-024-47802-7)
Supplement: Supplementary file 3 — Description of Additional Supplementary Files [file 41467_2024_47802_MOESM3_ESM.pdf]

Description of Additional Supplementary Files

File Name: Supplementary Data 1
Description: This file contains the 70 independent population-level vQTL reaching genome-wide significance (P < 1x10-8) across 8 traits; namely BMI, weight, fat percentage, hip circumference, waist circumference, and waist:hip ratio.
