# Supplementary material for: Genetic influence on within-person longitudinal change in anthropometric traits in the UK Biobank
Source: Nat Commun. 2024 May 6;15:3776. doi: 10.1038/s41467-024-47802-7 (PMC11074304; doi:10.1038/s41467-024-47802-7)
Supplement: Supplementary file 5 — Reporting Summary [file 41467_2024_47802_MOESM5_ESM.pdf]

nature portfolio

Corresponding author(s): Kathryn Kemper

Last updated by author(s): Mar 13, 2024

Reporting Summary

Nature Portfolio wishes to improve the reproducibility of the work that we publish. This form provides structure for consistency and transparency in reporting. For further information on Nature Portfolio policies, see our Editorial Policies and the Editorial Policy Checklist.

Statistics

For all statistical analyses, confirm that the following items are present in the figure legend, table legend, main text, or Methods section.

n/a	Confirmed	
☐	☒	The exact sample size (*n*) for each experimental group/condition, given as a discrete number and unit of measurement
☐	☒	A statement on whether measurements were taken from distinct samples or whether the same sample was measured repeatedly
☐	☒	The statistical test(s) used AND whether they are one- or two-sided<br>*Only common tests should be described solely by name; describe more complex techniques in the Methods section.*
☐	☒	A description of all covariates tested
☐	☒	A description of any assumptions or corrections, such as tests of normality and adjustment for multiple comparisons
☐	☒	A full description of the statistical parameters including central tendency (e.g. means) or other basic estimates (e.g. regression coefficient) AND variation (e.g. standard deviation) or associated estimates of uncertainty (e.g. confidence intervals)
☐	☒	For null hypothesis testing, the test statistic (e.g. F, t, r) with confidence intervals, effect sizes, degrees of freedom and P value noted<br>*Give P values as exact values whenever suitable.*
☒	☐	For Bayesian analysis, information on the choice of priors and Markov chain Monte Carlo settings
☒	☐	For hierarchical and complex designs, identification of the appropriate level for tests and full reporting of outcomes
☒	☐	Estimates of effect sizes (e.g. Cohen's *d*, Pearson's *r*), indicating how they were calculated

Our web collection on statistics for biologists contains articles on many of the points above.

Software and code

Policy information about availability of computer code

Data collection

Authors involved in this study were not involved in the data collection process.

Data analysis

We primarily used publicly available software for data analysis. Figures were created using R and Rstudio.

Software used in this study are available at the following URLs:
PLINK v1.9: https://www.cog-genomics.org/plink/1.9/
PLINK v2.0: https://www.cog-genomics.org/plink/2.0/
R v4.2.1: https://www.r-project.org/
R Studio 2023.06.2 build 561: https://posit.co/products/open-source/rstudio/
GCTA v1.93.2 beta: https://yanglab.westlake.edu.cn/software/gcta/#Overview
OSCA v0.46 https://yanglab.westlake.edu.cn/software/osca/#Overview

For manuscripts utilizing custom algorithms or software that are central to the research but not yet described in published literature, software must be made available to editors and reviewers. We strongly encourage code deposition in a community repository (e.g. GitHub). See the Nature Portfolio guidelines for submitting code & software for further information.

Data

Policy information about availability of data

All manuscripts must include a data availability statement. This statement should provide the following information, where applicable:

- Accession codes, unique identifiers, or web links for publicly available datasets
- A description of any restrictions on data availability
- For clinical datasets or third party data, please ensure that the statement adheres to our policy

Data from this study is available from the UK Biobank. Data access policies (http://www.ukbiobank.ac.uk/register-apply/) and a description of the genetic data (http://www.ukbiobank.ac.uk/scientists-3/genetic-data/) are available from the UK Biobank website.
We downloaded the genome-wide summary statics for Alzheimer's disease (excluding UK Biobank and 23andMe samples) reported in Wightman et al. from the Complex Traits Genetics lab website (https://ctg.cncr.nl/documents/p1651/PGCALZ2ExcludingUKBand23andME_METALInverseVariance_MetaAnalysis.txt.gz), the male and female pooled summary statistics reported in Estrada et al. for femoral neck and lumbar spine bone mineral density from the genetic factors for osteoporosis consortium website (http://www.gefos.org/sites/default/files/GEFOS2_FNBMD_POOLED_GC.txt.gz and http://www.gefos.org/sites/default/files/GEFOS2_LSBMD_POOLED_GC.txt.gz), and the CADIoGRAM meta-analysis statics reported in Schunkert et al. from the CARDIoGRAMEplusC4D consortium website (http://www.cardiogramplusc4d.org/media/cardiogramplusc4d-consortium/data-downloads/cardiogram_gwas_results.zip).
Data and scripts to reproduce figures and tables are provided in the Source data provided with this paper. Summary Statistics for BMI, weight, height and sitting height rate-change; plus the case-control analysis of single and repeat measures participants in the UK Biobank are available for download on the XXXX website.

Research involving human participants, their data, or biological material

Policy information about studies with human participants or human data. See also policy information about sex, gender (identity/presentation), and sexual orientation and race, ethnicity and racism.

Reporting on sex and gender	We only use the term 'sex' throughout the manuscript as we were interested in biological sex. Sex was used as a covariate in all analyses to remove the mean differences between males and females.
Reporting on race, ethnicity, or other socially relevant groupings	Our analysis was restricted to individuals with inferred Western European and British ancestry as this was the largest ancestry cohort available in the UK Biobank. Ancestry definitions were from a previous paper (Kemper et al. Nat Comms 2021) where, briefly, UK Biobank individuals were projected onto the first two principal components from the 1000 Genomes Project reference panel using GCTA, and assigned to the GBR (British in England and Scotland) and CEU (Northern and Western European ancestry) cluster with >0.9 probability.
Population characteristics	Participants were between 40 and 69 years when they enrolled in the study between 2006 and 2010. Individuals were recruited from England, Wales and Scotland. The population consists of approximately 54% female participants.
Recruitment	Invitations to participate in the UK Biobank were sent to 9.2M individuals registered with the UK's National Health Service who were 40-69 years in 2006-2010 and lived within 40km of one of 22 assessment centers located in England, Wales and Scotland.
Ethics oversight	The UK Biobank study was proved by the North West Centre for Research Ethics Committee (11/NW/0382). Participants volunteered for the study and provided signed electronic consent on recruitment. Further details on the ethics and governance frame work of the UK Biobank is available on the UK Biobank website (https://www.ukbiobank.ac.uk/media/0xsbmfmw/egf.pdf). The research in this manuscript is approved under the University of Queensland human ethics committee (approval number 201100173).

Note that full information on the approval of the study protocol must also be provided in the manuscript.

Field-specific reporting

Please select the one below that is the best fit for your research. If you are not sure, read the appropriate sections before making your selection.

☒ Life sciences ☐ Behavioural & social sciences ☐ Ecological, evolutionary & environmental sciences

For a reference copy of the document with all sections, see nature.com/documents/nr-reporting-summary-flat.pdf

Life sciences study design

All studies must disclose on these points even when the disclosure is negative.

Sample size	Data was obtained from the UK Biobank study, authors were not involved in the data collection and had no role in determining sample size. The UK Biobank is a very large cohort with repeat assessments available. We used the largest sample size available for individuals with repeated measures, after the exclusions noted below.
Data exclusions	The UK Biobank consists of a sample of ~500K individuals. We excluded samples with (1) non-European ancestry (based on Principal Component analysis and by comparison to the 1000 genomes European sample), (2) inconsistent self-reported and genetic sex, (3) born prior to 1937 and of after 1970, (4) younger than 40 or older than 70 at the time of baseline assessment, (5) failed genotype quality control (as outlined by Bycroft et al. 2018) and (6) had withdrawn consent. These exclusion criteria are based on data availability and best-practice criteria for genomic analysis. From this set of individuals, we identified those with repeat assessments but excluded one member of a relative

pair (if relatives were detected, pi > 0.05; see methods). From the set of individual with only baseline measurements, we further excluded any individuals who were related to individuals with repeat assessments (pi > 0.05), and then also removed one member of any pair within the set identified as relatives (pi > 0.05). The data used for analysis consisted of a set of unrelated individuals with repeat assessments (N = 50,117) and a set of unrelated individuals with baseline (only) measurements (N = 284,165), all with inferred British and Western European ancestry.

Replication: We have not attempted to replicate our results as a similar sized dataset for replication is not available.

Randomization: Not applicable as our study was not experimental and there are no treatment groups.

Blinding: Not applicable as our study was not experimental and there are no treatment groups.

Reporting for specific materials, systems and methods

We require information from authors about some types of materials, experimental systems and methods used in many studies. Here, indicate whether each material, system or method listed is relevant to your study. If you are not sure if a list item applies to your research, read the appropriate section before selecting a response.

Materials & experimental systems

n/a	Involved in the study
☒	☐ Antibodies
☒	☐ Eukaryotic cell lines
☒	☐ Palaeontology and archaeology
☒	☐ Animals and other organisms
☒	☐ Clinical data
☒	☐ Dual use research of concern
☒	☐ Plants

Methods

n/a	Involved in the study
☒	☐ ChIP-seq
☒	☐ Flow cytometry
☒	☐ MRI-based neuroimaging

Plants

Seed stocks: *Report on the source of all seed stocks or other plant material used. If applicable, state the seed stock centre and catalogue number. If plant specimens were collected from the field, describe the collection location, date and sampling procedures.*

Novel plant genotypes: *Describe the methods by which all novel plant genotypes were produced. This includes those generated by transgenic approaches, gene editing, chemical/radiation-based mutagenesis and hybridization. For transgenic lines, describe the transformation method, the number of independent lines analyzed and the generation upon which experiments were performed. For gene-edited lines, describe the editor used, the endogenous sequence targeted for editing, the targeting guide RNA sequence (if applicable) and how the editor was applied.*

Authentication: *Describe any authentication procedures for each seed stock used or novel genotype generated. Describe any experiments used to assess the effect of a mutation and, where applicable, how potential secondary effects (e.g. second site T-DNA insertions, mosiacism, off-target gene editing) were examined.*
