# Supplementary material for: Genetic influence on within-person longitudinal change in anthropometric traits in the UK Biobank
Source: Nat Commun. 2024 May 6;15:3776. doi: 10.1038/s41467-024-47802-7 (PMC11074304; doi:10.1038/s41467-024-47802-7)
Supplement: Supplementary file 6 — Source Data [file 41467_2024_47802_MOESM6_ESM.zip › data/3_rateGWAS/rateGwas.html]

GWAS ageCorrected trait change - repeated measures UKBiobank


# GWAS ageCorrected trait change - repeated measures UKBiobank

#### by *Kathryn Kemper* - 12 March 2024

```
manhatten.plot<-function(chr,pVal) {
   num = 1:length(chr)
   log10 = -log10(pVal)
   cols = rep("grey",length(num))
   cols[chr%%2==0]="dark blue"
   midPoints = aggregate(num~as.factor(chr),FUN=mean)
   { plot(log10~num, col=cols, pch=20, las=1, xaxt="n",xlab="Chromosome"
      ,ylab=expression(-log[10](P)))
     axis(1,at=midPoints[,2], labels=midPoints[,1], cex.lab=0.8)
   }
}
read.files<-function(trait,num) {
   data=NULL
   for (i in 1:22) {
       tmp=read.table(paste0(trait,"_ageCorrected",num,"_chr",i,".fastGWA"),header=T)
       tmp=tmp[tmp$P<0.2,]
       data=rbind(data,tmp)
   }
   return(data)
}
```

```
   height=read.files("height",1) #mean
   manhatten.plot(height$CHR,height$P)
```

```
   height=read.files("height",2) #rate
   manhatten.plot(height$CHR,height$P)
```

```
   height[height$P<5*10^(-8),]
```

```
##  [1] CHR  SNP  POS  A1   A2   N    AF1  BETA SE   P   
## <0 rows> (or 0-length row.names)
```

```
   rm(height)

   sit=read.files("sit",1) #mean
   manhatten.plot(sit$CHR,sit$P)
```

```
   sit=read.files("sit",2) #rate
   manhatten.plot(sit$CHR,sit$P)
```

```
   sit[sit$P<5*10^(-8),]
```

```
##         CHR       SNP     POS A1 A2     N      AF1       BETA        SE
## 2580310   8 rs2552207 6046104  G  C 49502 0.332673 0.00960048 0.0017563
## 258047    8 rs2570647 6046150  C  A 49502 0.332673 0.00960048 0.0017563
##                   P
## 2580310 4.59529e-08
## 258047  4.59529e-08
```

```
   rm(sit)

   weight=read.files("weight",1) #mean
   manhatten.plot(weight$CHR,weight$P)
```

```
   weight=read.files("weight",2) #rate
   manhatten.plot(weight$CHR,weight$P)
```

```
   weight[weight$P<5*10^(-8),]
```

```
##         CHR        SNP      POS A1 A2     N      AF1      BETA         SE
## 1027634  19   rs769449 45410002  G  A 49999 0.875027 0.0463249 0.00757728
## 1027659  19   rs429358 45411941  T  C 49999 0.847387 0.0467456 0.00698212
## 1027744  19 rs10414043 45415713  G  A 49722 0.874734 0.0436987 0.00758914
## 1027753  19  rs7256200 45415935  G  T 49710 0.874573 0.0438298 0.00758644
##                   P
## 1027634 9.73673e-10
## 1027659 2.15612e-11
## 1027744 8.50864e-09
## 1027753 7.58712e-09
```

```
   rm(weight)

   bmi=read.files("bmi",1) #mean
   manhatten.plot(bmi$CHR,bmi$P)
```

```
   bmi=read.files("bmi",2) #rate
   manhatten.plot(bmi$CHR,bmi$P)
```

```
   bmi[bmi$P<5*10^(-8),]
```

```
##         CHR        SNP      POS A1 A2     N      AF1      BETA         SE
## 1027674  19   rs769449 45410002  G  A 49968 0.875060 0.0161786 0.00267354
## 1027695  19   rs429358 45411941  T  C 49968 0.847412 0.0160279 0.00246344
## 1027783  19 rs10414043 45415713  G  A 49691 0.874766 0.0152309 0.00267773
## 1027792  19  rs7256200 45415935  G  T 49679 0.874605 0.0152992 0.00267678
##                   P
## 1027674 1.43620e-09
## 1027695 7.70167e-11
## 1027783 1.28531e-08
## 1027792 1.09358e-08
```

```
   rm(bmi)
```
