# Supplementary material for: Genetic influence on within-person longitudinal change in anthropometric traits in the UK Biobank
Source: Nat Commun. 2024 May 6;15:3776. doi: 10.1038/s41467-024-47802-7 (PMC11074304; doi:10.1038/s41467-024-47802-7)
Supplement: Supplementary file 6 — Source Data [file 41467_2024_47802_MOESM6_ESM.zip › data/3_rateGWAS/APOE.html]

investigation into APOE allele frequency & effects


# investigation into APOE allele frequency & effects

#### by *Kathryn Kemper* - 22 December 2023

```
## ── Attaching packages ─────────────────────────────────────── tidyverse 1.3.1 ──
```

```
## ✔ tibble  3.1.7     ✔ dplyr   1.0.9
## ✔ tidyr   1.2.0     ✔ stringr 1.4.0
## ✔ readr   2.1.2     ✔ forcats 0.5.1
## ✔ purrr   0.3.4
```

```
## ── Conflicts ────────────────────────────────────────── tidyverse_conflicts() ──
## ✖ dplyr::filter() masks stats::filter()
## ✖ dplyr::lag()    masks stats::lag()
```

# Check the GWAS results

```
   lm0 = lm(weight[,4]~weight[,8]+weight[,9]+weight[,10]+weight[,11]+weight[,12]+
      weight[,13]+weight[,14]+weight[,15]+weight[,16]+weight[,17]+
      weight[,18]+weight[,19]+weight[,20]+weight[,21]+weight[,22]+
      weight[,23]+weight[,24]+weight[,25]+weight[,26]+weight[,27]+
      weight[,28]+weight[,29]+weight[,30]+weight[,31]+weight[,32]+
      weight$APOE)
   summary(lm0) #PC1-25 are weight cols 8-32
```

```
## 
## Call:
## lm(formula = weight[, 4] ~ weight[, 8] + weight[, 9] + weight[, 
##     10] + weight[, 11] + weight[, 12] + weight[, 13] + weight[, 
##     14] + weight[, 15] + weight[, 16] + weight[, 17] + weight[, 
##     18] + weight[, 19] + weight[, 20] + weight[, 21] + weight[, 
##     22] + weight[, 23] + weight[, 24] + weight[, 25] + weight[, 
##     26] + weight[, 27] + weight[, 28] + weight[, 29] + weight[, 
##     30] + weight[, 31] + weight[, 32] + weight$APOE)
## 
## Residuals:
##     Min      1Q  Median      3Q     Max 
## -7.1914 -0.3619  0.0159  0.3966  7.1018 
## 
## Coefficients:
##               Estimate Std. Error t value Pr(>|t|)    
## (Intercept)   0.007973   0.004328   1.842 0.065466 .  
## weight[, 8]   0.941019   0.318346   2.956 0.003118 ** 
## weight[, 9]  -0.966365   0.462535  -2.089 0.036688 *  
## weight[, 10] -0.925592   0.446402  -2.073 0.038136 *  
## weight[, 11] -0.430382   0.511033  -0.842 0.399691    
## weight[, 12] -1.720530   0.464904  -3.701 0.000215 ***
## weight[, 13]  0.462626   0.770169   0.601 0.548055    
## weight[, 14] -0.888806   0.618915  -1.436 0.150989    
## weight[, 15] -0.056567   0.617449  -0.092 0.927006    
## weight[, 16]  0.341780   0.638916   0.535 0.592696    
## weight[, 17] -0.226386   0.660768  -0.343 0.731893    
## weight[, 18] -0.677555   0.662452  -1.023 0.306409    
## weight[, 19] -0.805737   0.673828  -1.196 0.231796    
## weight[, 20]  0.530707   0.689759   0.769 0.441654    
## weight[, 21]  0.259432   0.681393   0.381 0.703400    
## weight[, 22] -0.360891   0.670967  -0.538 0.590671    
## weight[, 23] -0.062231   0.673112  -0.092 0.926339    
## weight[, 24]  0.046274   0.671792   0.069 0.945084    
## weight[, 25] -0.626645   0.674263  -0.929 0.352698    
## weight[, 26] -0.151052   0.675223  -0.224 0.822986    
## weight[, 27]  0.958047   0.674502   1.420 0.155505    
## weight[, 28] -0.187124   0.679265  -0.275 0.782948    
## weight[, 29]  0.529231   0.675058   0.784 0.433056    
## weight[, 30] -0.003013   0.675583  -0.004 0.996441    
## weight[, 31] -0.154117   0.677672  -0.227 0.820097    
## weight[, 32]  0.695729   0.676341   1.029 0.303642    
## weight$APOE  -0.046795   0.006988  -6.697 2.15e-11 ***
## ---
## Signif. codes:  0 '***' 0.001 '**' 0.01 '*' 0.05 '.' 0.1 ' ' 1
## 
## Residual standard error: 0.7937 on 49972 degrees of freedom
## Multiple R-squared:  0.001733,   Adjusted R-squared:  0.001214 
## F-statistic: 3.337 on 26 and 49972 DF,  p-value: 1.874e-08
```

```
   # variance explained
   alpha = -0.046795 
   p = mean(weight$APOE)/2
   p # rs429358 frequency
```

```
## [1] 0.1526131
```

```
   2*p*(1-p)*alpha^2
```

```
## [1] 0.0005663727
```

```
   var(weight[,4]) #phenotypic variance
```

```
## [1] 0.6306829
```

```
   (2*p*(1-p)*alpha^2)/var(weight[,4]) #proportion variance explained
```

```
## [1] 0.0008980309
```

```
   # power to detect
   N = 50000              # sample size
   alpha = 5*10^(-8)     # significance threshold
   h2 = 0.0009             # variance explained by QTL
   threshold = qchisq(alpha, df = 1, lower.tail = FALSE)
   power = pchisq(threshold, df = 1, lower.tail = FALSE, ncp = N * h2)
   power
```

```
## [1] 0.8956039
```

```
   # effect on the mean weight
   lmM = lm(weight[,3]~weight[,8]+weight[,9]+weight[,10]+weight[,11]+weight[,12]+
      weight[,13]+weight[,14]+weight[,15]+weight[,16]+weight[,17]+
      weight[,18]+weight[,19]+weight[,20]+weight[,21]+weight[,22]+
      weight[,23]+weight[,24]+weight[,25]+weight[,26]+weight[,27]+
      weight[,28]+weight[,29]+weight[,30]+weight[,31]+weight[,32]+
      weight$APOE)
   summary(lmM)
```

```
## 
## Call:
## lm(formula = weight[, 3] ~ weight[, 8] + weight[, 9] + weight[, 
##     10] + weight[, 11] + weight[, 12] + weight[, 13] + weight[, 
##     14] + weight[, 15] + weight[, 16] + weight[, 17] + weight[, 
##     18] + weight[, 19] + weight[, 20] + weight[, 21] + weight[, 
##     22] + weight[, 23] + weight[, 24] + weight[, 25] + weight[, 
##     26] + weight[, 27] + weight[, 28] + weight[, 29] + weight[, 
##     30] + weight[, 31] + weight[, 32] + weight$APOE)
## 
## Residuals:
##     Min      1Q  Median      3Q     Max 
## -37.690  -8.802  -1.726   6.829  99.293 
## 
## Coefficients:
##               Estimate Std. Error t value Pr(>|t|)    
## (Intercept)    0.03783    0.06945   0.545 0.585924    
## weight[, 8]    5.37094    5.10810   1.051 0.293054    
## weight[, 9]  -12.21630    7.42172  -1.646 0.099766 .  
## weight[, 10] -29.71650    7.16286  -4.149 3.35e-05 ***
## weight[, 11]   1.64561    8.19991   0.201 0.840945    
## weight[, 12] -12.51498    7.45973  -1.678 0.093418 .  
## weight[, 13] -31.50270   12.35794  -2.549 0.010800 *  
## weight[, 14]   1.54535    9.93096   0.156 0.876342    
## weight[, 15]  29.48706    9.90742   2.976 0.002919 ** 
## weight[, 16]  11.52824   10.25189   1.124 0.260807    
## weight[, 17] -28.78522   10.60252  -2.715 0.006631 ** 
## weight[, 18] -26.78555   10.62955  -2.520 0.011741 *  
## weight[, 19] -38.28659   10.81207  -3.541 0.000399 ***
## weight[, 20] -12.97143   11.06769  -1.172 0.241199    
## weight[, 21] -23.50651   10.93346  -2.150 0.031563 *  
## weight[, 22]  22.37479   10.76617   2.078 0.037691 *  
## weight[, 23]  12.82020   10.80059   1.187 0.235237    
## weight[, 24]   3.14791   10.77941   0.292 0.770265    
## weight[, 25] -28.68267   10.81905  -2.651 0.008025 ** 
## weight[, 26]   1.95982   10.83446   0.181 0.856457    
## weight[, 27]  19.28248   10.82290   1.782 0.074814 .  
## weight[, 28]  15.79473   10.89932   1.449 0.147303    
## weight[, 29]   3.08097   10.83181   0.284 0.776077    
## weight[, 30]  -0.57127   10.84023  -0.053 0.957972    
## weight[, 31]   7.86789   10.87375   0.724 0.469335    
## weight[, 32] -15.92585   10.85240  -1.467 0.142247    
## weight$APOE   -0.29895    0.11212  -2.666 0.007671 ** 
## ---
## Signif. codes:  0 '***' 0.001 '**' 0.01 '*' 0.05 '.' 0.1 ' ' 1
## 
## Residual standard error: 12.74 on 49972 degrees of freedom
## Multiple R-squared:  0.001973,   Adjusted R-squared:  0.001453 
## F-statistic: 3.799 on 26 and 49972 DF,  p-value: 2.116e-10
```

# Do individuals in repeated measures sample have AD diagnosis?

Is this diagnosis more frequent than that found in the single
measurement subset of the UKB?

Are individuals diagnosed before or after their final assessment?

```
aggregate(geno$alz~geno$set,FUN=sum)
```

```
##   geno$set geno$alz
## 1       RM       93
## 2   single     1789
```

```
aggregate(geno$alz~geno$set,FUN=mean)
```

```
##   geno$set    geno$alz
## 1       RM 0.001855658
## 2   single 0.006295638
```

```
# only 93 individuals in repeated measures set with AD diagnosis. 
# almost 0.2% of RM population, vs. 0.6% in single measurement set.

s = match(RM2[,1],alz)
RM2$alzDate = alzDate[s]
s = match(RM2[,1],dets[,1])
dets2=dets[s,]
RM2$baseDate=dets2[,4]
RM2$rmDate=dets2[,5]        #set all to 1st review then overwrite
RM2$rmDate[RM2$interval==2]=dets2[RM2$interval==2,6]
RM2$rmDate[RM2$interval==3]=dets2[RM2$interval==3,7]
RM2$baseDate=as.Date(RM2$baseDate)
RM2$rmDate=as.Date(RM2$rmDate)
RM2$alzDate=as.Date(RM2$alzDate)
table(RM2$alzDate>RM2$rmDate)
```

```
## 
## FALSE  TRUE 
##     6    69
```

```
sum(is.na(RM2[RM2$alz,"alzDate"]))
```

```
## [1] 50116
```

```
# there are 93 individuals with AD in repeated measures set
# most (n = 69, 74.2%) were not diagnosed with AD BEFORE their 2nd assessment
# some were (n = 6) and some did not have recorded date of diagnosis (n = 18)

# even if we exclude the individuals with a diagnosis (n = 93), the effect of allele is largely unchanged.

weight2 = weight[!weight$ID%in%alz,]
lm(weight2[,4]~weight2[,8]+weight2[,9]+weight2[,10]+weight2[,11]+weight2[,12]+
      weight2[,13]+weight2[,14]+weight2[,15]+weight2[,16]+weight2[,17]+
      weight2[,18]+weight2[,19]+weight2[,20]+weight2[,21]+weight2[,22]+
      weight2[,23]+weight2[,24]+weight2[,25]+weight2[,26]+weight2[,27]+
      weight2[,28]+weight2[,29]+weight2[,30]+weight2[,31]+weight2[,32]+
      weight2$APOE)
```

```
## 
## Call:
## lm(formula = weight2[, 4] ~ weight2[, 8] + weight2[, 9] + weight2[, 
##     10] + weight2[, 11] + weight2[, 12] + weight2[, 13] + weight2[, 
##     14] + weight2[, 15] + weight2[, 16] + weight2[, 17] + weight2[, 
##     18] + weight2[, 19] + weight2[, 20] + weight2[, 21] + weight2[, 
##     22] + weight2[, 23] + weight2[, 24] + weight2[, 25] + weight2[, 
##     26] + weight2[, 27] + weight2[, 28] + weight2[, 29] + weight2[, 
##     30] + weight2[, 31] + weight2[, 32] + weight2$APOE)
## 
## Coefficients:
##   (Intercept)   weight2[, 8]   weight2[, 9]  weight2[, 10]  weight2[, 11]  
##      0.007975       0.941012      -0.956494      -0.928968      -0.406038  
## weight2[, 12]  weight2[, 13]  weight2[, 14]  weight2[, 15]  weight2[, 16]  
##     -1.750180       0.441768      -0.850423      -0.055421       0.315292  
## weight2[, 17]  weight2[, 18]  weight2[, 19]  weight2[, 20]  weight2[, 21]  
##     -0.205222      -0.761809      -0.793957       0.564796       0.288873  
## weight2[, 22]  weight2[, 23]  weight2[, 24]  weight2[, 25]  weight2[, 26]  
##     -0.364051      -0.097929       0.047055      -0.651035      -0.155631  
## weight2[, 27]  weight2[, 28]  weight2[, 29]  weight2[, 30]  weight2[, 31]  
##      0.932085      -0.192647       0.547135       0.064829      -0.100967  
## weight2[, 32]   weight2$APOE  
##      0.731788      -0.046364
```

# Assessing degree of bias in frequency of AD APOE risk ‘C’ allele in repeated measures sample, compared to single measurement sample in UKB

## dependence on diagnosis of AD

```
   geno$grp=paste(geno$set,geno$alz,sep="_")
   dets$class=cut(dets$ageBase,breaks=seq(39,70,5))
   data=aggregate(geno$count~geno$grp+dets$class,FUN=mean)
   data=cbind(data,aggregate(geno$count~geno$grp+dets$class,FUN=length)[,3])
   data$frequency=data[,3]/2
   names(data)[1:4]=c("set","class","mean","count")
   data
```

```
##             set   class      mean count frequency
## 1      RM_FALSE (39,44] 0.3065570  4743 0.1532785
## 2  single_FALSE (39,44] 0.3128280 27245 0.1564140
## 3   single_TRUE (39,44] 0.6666667     6 0.3333333
## 4      RM_FALSE (44,49] 0.3211816  6940 0.1605908
## 5       RM_TRUE (44,49] 0.0000000     2 0.0000000
## 6  single_FALSE (44,49] 0.3173964 35996 0.1586982
## 7   single_TRUE (44,49] 0.5833333    12 0.2916667
## 8      RM_FALSE (49,54] 0.3105033  8702 0.1552517
## 9       RM_TRUE (49,54] 1.0000000     4 0.5000000
## 10 single_FALSE (49,54] 0.3135659 42629 0.1567829
## 11  single_TRUE (49,54] 0.5306122    49 0.2653061
## 12     RM_FALSE (54,59] 0.3093994 10905 0.1546997
## 13      RM_TRUE (54,59] 0.7142857     7 0.3571429
## 14 single_FALSE (54,59] 0.3070874 50992 0.1535437
## 15  single_TRUE (54,59] 0.7786260   131 0.3893130
## 16     RM_FALSE (59,64] 0.2971210 12261 0.1485605
## 17      RM_TRUE (59,64] 0.7428571    35 0.3714286
## 18 single_FALSE (59,64] 0.3065121 70914 0.1532561
## 19  single_TRUE (59,64] 0.8326996   526 0.4163498
## 20     RM_FALSE (64,69] 0.2814769  6473 0.1407385
## 21      RM_TRUE (64,69] 0.7333333    45 0.3666667
## 22 single_FALSE (64,69] 0.2978022 54600 0.1489011
## 23  single_TRUE (64,69] 0.7737089  1065 0.3868545
```

```
   data = data[data[,4]>9,] #remove groups with fewer than 10 counts for plot
   data$set[data$set=="RM_FALSE"] = "repeat_noAD"
   data$set[data$set=="RM_TRUE"] = "repeat_AD"
   data$set[data$set=="single_FALSE"] = "single_noAD"
   data$set[data$set=="single_TRUE"] = "single_AD"
   data$AD = "no" ; data$AD[data$set=="repeat_AD"]="yes" ; data$AD[data$set=="single_AD"]="yes"
   data$repeatedMeasures="no"; data$repeatedMeasures[data$set=="repeat_AD"]="yes" 
   data$repeatedMeasures[data$set=="repeat_noAD"]="yes"  
 
  ggplot(data,aes(x=class,y=frequency,group=set))+
    geom_line(aes(color=AD,linetype=repeatedMeasures),size=1.5)+geom_point()+
    xlab("age at baseline")+
    theme_minimal(base_size=20) + 
    scale_color_manual(values=wes_palette(n=3, name="GrandBudapest1")) +
    scale_linetype_manual(values=c(1,3))
```

```
   # frequency of allele is about 15-16% in individuals without diagnosis in both subsets until the age of 60. Thereafter frequency of risk allele starts dropping in repeated measures cohort - suggesting recall bias in older age groups.
   # frequency in diagnosed individuals 30-40%.

   aggregate(geno[,1]~geno$count+geno$set+geno$alz,FUN=length)
```

```
##    geno$count geno$set geno$alz geno[, 1]
## 1           0       RM    FALSE     35946
## 2           1       RM    FALSE     12932
## 3           2       RM    FALSE      1146
## 4           0   single    FALSE    201965
## 5           1   single    FALSE     73852
## 6           2   single    FALSE      6559
## 7           0       RM     TRUE        36
## 8           1       RM     TRUE        46
## 9           2       RM     TRUE        11
## 10          0   single     TRUE       664
## 11          1   single     TRUE       849
## 12          2   single     TRUE       276
```
