# Supplementary material for: Genetic influence on within-person longitudinal change in anthropometric traits in the UK Biobank
Source: Nat Commun. 2024 May 6;15:3776. doi: 10.1038/s41467-024-47802-7 (PMC11074304; doi:10.1038/s41467-024-47802-7)
Supplement: Supplementary file 6 — Source Data [file 41467_2024_47802_MOESM6_ESM.zip › data/5_disease+PGS/diseaseAssocV3.html]

Association of rate-of-trait-change with disease - UKBiobank


# Association of rate-of-trait-change with disease - UKBiobank

#### by *Kathryn Kemper* - 06 November 2023

# 1. Diagnoses defined

```
   # incidence in independent UKB data
   table(data$death) ; mean(data$death)
```

```
## 
##  FALSE   TRUE 
## 260954  23211
```

```
## [1] 0.08168142
```

```
   table(data$mof) ; mean(data$mof)
```

```
## 
##  FALSE   TRUE 
## 275307   8858
```

```
## [1] 0.03117203
```

```
   table(data$lsf) ; mean(data$lsf)
```

```
## 
##  FALSE   TRUE 
## 283696    469
```

```
## [1] 0.00165045
```

```
   table(data$cad) ; mean(data$cad)
```

```
## 
##  FALSE   TRUE 
## 272713  11452
```

```
## [1] 0.04030053
```

```
   table(data$alz) ; mean(data$alz)
```

```
## 
##  FALSE   TRUE 
## 282376   1789
```

```
## [1] 0.006295638
```

```
   # incidence in repeated measures data
   table(phen$death) ; mean(phen$death)
```

```
## 
## FALSE  TRUE 
## 48882  1198
```

```
## [1] 0.02392173
```

```
   table(phen$mof) ; mean(phen$mof)
```

```
## 
## FALSE  TRUE 
## 49232   848
```

```
## [1] 0.01693291
```

```
   table(phen$lsf) ; mean(phen$lsf)
```

```
## 
## FALSE  TRUE 
## 50016    64
```

```
## [1] 0.001277955
```

```
   table(phen$cad) ; mean(phen$cad)
```

```
## 
## FALSE  TRUE 
## 48640  1440
```

```
## [1] 0.02875399
```

```
   table(phen$alz) ; mean(phen$alz)
```

```
## 
## FALSE  TRUE 
## 49988    92
```

```
## [1] 0.001837061
```

# 2. Phenotypic association between death and trait-change

```
deathHeight=glm(death~height.std+height2.std,family=binomial(link='logit'),data=phen)
deathWeight=glm(death~weight.std+weight2.std,family=binomial(link='logit'),data=phen)
deathSit=glm(death~sit.std+sit2.std,family=binomial(link='logit'),data=phen)
deathBMI=glm(death~bmi.std+bmi2.std,family=binomial(link='logit'),data=phen)
summary(deathHeight)
```

```
## 
## Call:
## glm(formula = death ~ height.std + height2.std, family = binomial(link = "logit"), 
##     data = phen)
## 
## Deviance Residuals: 
##     Min       1Q   Median       3Q      Max  
## -1.3598  -0.2243  -0.2084  -0.1998   2.8704  
## 
## Coefficients:
##              Estimate Std. Error  z value Pr(>|z|)    
## (Intercept) -3.786408   0.031034 -122.006  < 2e-16 ***
## height.std  -0.216866   0.028313   -7.660 1.86e-14 ***
## height2.std  0.037122   0.007304    5.082 3.73e-07 ***
## ---
## Signif. codes:  0 '***' 0.001 '**' 0.01 '*' 0.05 '.' 0.1 ' ' 1
## 
## (Dispersion parameter for binomial family taken to be 1)
## 
##     Null deviance: 11296  on 50071  degrees of freedom
## Residual deviance: 11154  on 50069  degrees of freedom
##   (8 observations deleted due to missingness)
## AIC: 11160
## 
## Number of Fisher Scoring iterations: 6
```

```
summary(deathWeight)
```

```
## 
## Call:
## glm(formula = death ~ weight.std + weight2.std, family = binomial(link = "logit"), 
##     data = phen)
## 
## Deviance Residuals: 
##     Min       1Q   Median       3Q      Max  
## -1.1315  -0.2178  -0.2151  -0.2143   2.7521  
## 
## Coefficients:
##              Estimate Std. Error  z value Pr(>|z|)    
## (Intercept) -3.764007   0.030695 -122.626  < 2e-16 ***
## weight.std  -0.002088   0.024971   -0.084    0.933    
## weight2.std  0.044298   0.006065    7.304  2.8e-13 ***
## ---
## Signif. codes:  0 '***' 0.001 '**' 0.01 '*' 0.05 '.' 0.1 ' ' 1
## 
## (Dispersion parameter for binomial family taken to be 1)
## 
##     Null deviance: 11284  on 49972  degrees of freedom
## Residual deviance: 11241  on 49970  degrees of freedom
##   (107 observations deleted due to missingness)
## AIC: 11247
## 
## Number of Fisher Scoring iterations: 6
```

```
summary(deathSit)
```

```
## 
## Call:
## glm(formula = death ~ sit.std + sit2.std, family = binomial(link = "logit"), 
##     data = phen)
## 
## Deviance Residuals: 
##     Min       1Q   Median       3Q      Max  
## -1.5027  -0.2245  -0.2118  -0.2029   2.9649  
## 
## Coefficients:
##              Estimate Std. Error  z value Pr(>|z|)    
## (Intercept) -3.765020   0.030795 -122.259  < 2e-16 ***
## sit.std     -0.212330   0.030271   -7.014 2.31e-12 ***
## sit2.std     0.017973   0.006997    2.569   0.0102 *  
## ---
## Signif. codes:  0 '***' 0.001 '**' 0.01 '*' 0.05 '.' 0.1 ' ' 1
## 
## (Dispersion parameter for binomial family taken to be 1)
## 
##     Null deviance: 11135  on 49648  degrees of freedom
## Residual deviance: 11041  on 49646  degrees of freedom
##   (431 observations deleted due to missingness)
## AIC: 11047
## 
## Number of Fisher Scoring iterations: 6
```

```
summary(deathBMI)
```

```
## 
## Call:
## glm(formula = death ~ bmi.std + bmi2.std, family = binomial(link = "logit"), 
##     data = phen)
## 
## Deviance Residuals: 
##     Min       1Q   Median       3Q      Max  
## -1.1740  -0.2184  -0.2151  -0.2139   2.7539  
## 
## Coefficients:
##              Estimate Std. Error  z value Pr(>|z|)    
## (Intercept) -3.761822   0.030631 -122.812  < 2e-16 ***
## bmi.std      0.034798   0.024325    1.431    0.153    
## bmi2.std     0.041712   0.005827    7.158 8.18e-13 ***
## ---
## Signif. codes:  0 '***' 0.001 '**' 0.01 '*' 0.05 '.' 0.1 ' ' 1
## 
## (Dispersion parameter for binomial family taken to be 1)
## 
##     Null deviance: 11269  on 49967  degrees of freedom
## Residual deviance: 11232  on 49965  degrees of freedom
##   (112 observations deleted due to missingness)
## AIC: 11238
## 
## Number of Fisher Scoring iterations: 6
```

```
plotData <- data.frame (index = 1:8,
            beta = c(deathHeight$coefficients[2:3],deathSit$coefficients[2:3],
                               deathWeight$coefficients[2:3],deathBMI$coefficients[2:3]),
                        lci = c(summary(deathHeight)$coefficients[2:3,1]-1.96*summary(deathHeight)$coefficients[2:3,2],summary(deathSit)$coefficients[2:3,1]-1.96*summary(deathSit)$coefficients[2:3,2],summary(deathWeight)$coefficients[2:3,1]-1.96*summary(deathWeight)$coefficients[2:3,2],summary(deathBMI)$coefficients[2:3,1]-1.96*summary(deathBMI)$coefficients[2:3,2]),
                        uci = c(summary(deathHeight)$coefficients[2:3,1]+1.96*summary(deathHeight)$coefficients[2:3,2],summary(deathSit)$coefficients[2:3,1]+1.96*summary(deathSit)$coefficients[2:3,2],summary(deathWeight)$coefficients[2:3,1]+1.96*summary(deathWeight)$coefficients[2:3,2],summary(deathBMI)$coefficients[2:3,1]+1.96*summary(deathBMI)$coefficients[2:3,2])
                        )
label = c("height change - linear","height change - quadratic",
         "sitting height change - linear","sitting height change - quadratic","weight change - linear",
          "weight change - quadratic","BMI change - linear","BMI change - quadratic")
plotData$OR = exp(plotData$beta)
plotData$OR.l = exp(plotData$lci)
plotData$OR.u = exp(plotData$uci)

colours = rep(brewer.pal(n = 8, name = "Paired")[c(2,4,6,8)],each=2)
plot1 <- ggplot(plotData, aes(y = index, x = OR)) +
  geom_point(shape = 15, size = 3, color = colours) +
  geom_linerange(aes(xmin = OR.l, xmax = OR.u), color=colours) +
  geom_vline(xintercept = 1, color = "grey", linetype = "dashed", alpha = 0.5) +
  scale_y_continuous(name = "", breaks=1:8, labels = label, trans = "reverse") +
  xlab("Odds Ratio (95% CI)") +
  ylab(" ") +
  theme_bw() +
  theme(axis.text=element_text(size=15),axis.title=element_text(size=15))
plot1
```

# 3. Association between diagnosis & slope PRS using an independent sample

```
## 
## Call:
## glm(formula = death ~ height.std, family = binomial(link = "logit"), 
##     data = data)
## 
## Deviance Residuals: 
##     Min       1Q   Median       3Q      Max  
## -0.4170  -0.4134  -0.4127  -0.4120   2.2457  
## 
## Coefficients:
##              Estimate Std. Error  z value Pr(>|z|)    
## (Intercept) -2.419727   0.006850 -353.270   <2e-16 ***
## height.std  -0.004798   0.006849   -0.701    0.484    
## ---
## Signif. codes:  0 '***' 0.001 '**' 0.01 '*' 0.05 '.' 0.1 ' ' 1
## 
## (Dispersion parameter for binomial family taken to be 1)
## 
##     Null deviance: 160756  on 284164  degrees of freedom
## Residual deviance: 160756  on 284163  degrees of freedom
## AIC: 160760
## 
## Number of Fisher Scoring iterations: 5
```

```
## 
## Call:
## glm(formula = death ~ sit.std, family = binomial(link = "logit"), 
##     data = data)
## 
## Deviance Residuals: 
##     Min       1Q   Median       3Q      Max  
## -0.4183  -0.4135  -0.4127  -0.4118   2.2481  
## 
## Coefficients:
##              Estimate Std. Error  z value Pr(>|z|)    
## (Intercept) -2.419732   0.006850 -353.269   <2e-16 ***
## sit.std     -0.005792   0.006849   -0.846    0.398    
## ---
## Signif. codes:  0 '***' 0.001 '**' 0.01 '*' 0.05 '.' 0.1 ' ' 1
## 
## (Dispersion parameter for binomial family taken to be 1)
## 
##     Null deviance: 160756  on 284164  degrees of freedom
## Residual deviance: 160755  on 284163  degrees of freedom
## AIC: 160759
## 
## Number of Fisher Scoring iterations: 5
```

```
## 
## Call:
## glm(formula = death ~ weight.std, family = binomial(link = "logit"), 
##     data = data)
## 
## Deviance Residuals: 
##     Min       1Q   Median       3Q      Max  
## -0.4140  -0.4130  -0.4128  -0.4126   2.2404  
## 
## Coefficients:
##              Estimate Std. Error  z value Pr(>|z|)    
## (Intercept) -2.419718   0.006849 -353.271   <2e-16 ***
## weight.std   0.001213   0.006849    0.177    0.859    
## ---
## Signif. codes:  0 '***' 0.001 '**' 0.01 '*' 0.05 '.' 0.1 ' ' 1
## 
## (Dispersion parameter for binomial family taken to be 1)
## 
##     Null deviance: 160756  on 284164  degrees of freedom
## Residual deviance: 160756  on 284163  degrees of freedom
## AIC: 160760
## 
## Number of Fisher Scoring iterations: 5
```

```
## 
## Call:
## glm(formula = death ~ bmi.std, family = binomial(link = "logit"), 
##     data = data)
## 
## Deviance Residuals: 
##     Min       1Q   Median       3Q      Max  
## -0.4154  -0.4132  -0.4128  -0.4123   2.2429  
## 
## Coefficients:
##              Estimate Std. Error z value Pr(>|z|)    
## (Intercept) -2.419721   0.006849 -353.27   <2e-16 ***
## bmi.std      0.002808   0.006849    0.41    0.682    
## ---
## Signif. codes:  0 '***' 0.001 '**' 0.01 '*' 0.05 '.' 0.1 ' ' 1
## 
## (Dispersion parameter for binomial family taken to be 1)
## 
##     Null deviance: 160756  on 284164  degrees of freedom
## Residual deviance: 160756  on 284163  degrees of freedom
## AIC: 160760
## 
## Number of Fisher Scoring iterations: 5
```

```
## 
## Call:
## glm(formula = mof ~ height.std, family = binomial(link = "logit"), 
##     data = data)
## 
## Deviance Residuals: 
##     Min       1Q   Median       3Q      Max  
## -0.2661  -0.2537  -0.2515  -0.2492   2.6725  
## 
## Coefficients:
##             Estimate Std. Error  z value Pr(>|z|)    
## (Intercept) -3.43689    0.01080 -318.289   <2e-16 ***
## height.std  -0.02618    0.01079   -2.426   0.0153 *  
## ---
## Signif. codes:  0 '***' 0.001 '**' 0.01 '*' 0.05 '.' 0.1 ' ' 1
## 
## (Dispersion parameter for binomial family taken to be 1)
## 
##     Null deviance: 78880  on 284164  degrees of freedom
## Residual deviance: 78874  on 284163  degrees of freedom
## AIC: 78878
## 
## Number of Fisher Scoring iterations: 6
```

```
## 
## Call:
## glm(formula = mof ~ sit.std, family = binomial(link = "logit"), 
##     data = data)
## 
## Deviance Residuals: 
##     Min       1Q   Median       3Q      Max  
## -0.2560  -0.2522  -0.2516  -0.2510   2.6466  
## 
## Coefficients:
##              Estimate Std. Error  z value Pr(>|z|)    
## (Intercept) -3.436591   0.010795 -318.353   <2e-16 ***
## sit.std     -0.007361   0.010794   -0.682    0.495    
## ---
## Signif. codes:  0 '***' 0.001 '**' 0.01 '*' 0.05 '.' 0.1 ' ' 1
## 
## (Dispersion parameter for binomial family taken to be 1)
## 
##     Null deviance: 78880  on 284164  degrees of freedom
## Residual deviance: 78880  on 284163  degrees of freedom
## AIC: 78884
## 
## Number of Fisher Scoring iterations: 6
```

```
## 
## Call:
## glm(formula = mof ~ weight.std, family = binomial(link = "logit"), 
##     data = data)
## 
## Deviance Residuals: 
##     Min       1Q   Median       3Q      Max  
## -0.2583  -0.2526  -0.2516  -0.2506   2.6490  
## 
## Coefficients:
##             Estimate Std. Error  z value Pr(>|z|)    
## (Intercept) -3.43663    0.01080 -318.345   <2e-16 ***
## weight.std  -0.01138    0.01079   -1.054    0.292    
## ---
## Signif. codes:  0 '***' 0.001 '**' 0.01 '*' 0.05 '.' 0.1 ' ' 1
## 
## (Dispersion parameter for binomial family taken to be 1)
## 
##     Null deviance: 78880  on 284164  degrees of freedom
## Residual deviance: 78879  on 284163  degrees of freedom
## AIC: 78883
## 
## Number of Fisher Scoring iterations: 6
```

```
## 
## Call:
## glm(formula = mof ~ bmi.std, family = binomial(link = "logit"), 
##     data = data)
## 
## Deviance Residuals: 
##     Min       1Q   Median       3Q      Max  
## -0.2549  -0.2522  -0.2516  -0.2511   2.6417  
## 
## Coefficients:
##              Estimate Std. Error  z value Pr(>|z|)    
## (Intercept) -3.436583   0.010795 -318.355   <2e-16 ***
## bmi.std     -0.006111   0.010795   -0.566    0.571    
## ---
## Signif. codes:  0 '***' 0.001 '**' 0.01 '*' 0.05 '.' 0.1 ' ' 1
## 
## (Dispersion parameter for binomial family taken to be 1)
## 
##     Null deviance: 78880  on 284164  degrees of freedom
## Residual deviance: 78880  on 284163  degrees of freedom
## AIC: 78884
## 
## Number of Fisher Scoring iterations: 6
```

```
## 
## Call:
## glm(formula = lsf ~ height.std, family = binomial(link = "logit"), 
##     data = data)
## 
## Deviance Residuals: 
##     Min       1Q   Median       3Q      Max  
## -0.0672  -0.0588  -0.0574  -0.0560   3.6434  
## 
## Coefficients:
##             Estimate Std. Error  z value Pr(>|z|)    
## (Intercept) -6.40765    0.04633 -138.295   <2e-16 ***
## height.std  -0.07216    0.04621   -1.561    0.118    
## ---
## Signif. codes:  0 '***' 0.001 '**' 0.01 '*' 0.05 '.' 0.1 ' ' 1
## 
## (Dispersion parameter for binomial family taken to be 1)
## 
##     Null deviance: 6946.7  on 284164  degrees of freedom
## Residual deviance: 6944.3  on 284163  degrees of freedom
## AIC: 6948.3
## 
## Number of Fisher Scoring iterations: 9
```

```
## 
## Call:
## glm(formula = lsf ~ sit.std, family = binomial(link = "logit"), 
##     data = data)
## 
## Deviance Residuals: 
##     Min       1Q   Median       3Q      Max  
## -0.0680  -0.0588  -0.0574  -0.0560   3.6348  
## 
## Coefficients:
##             Estimate Std. Error  z value Pr(>|z|)    
## (Intercept) -6.40759    0.04633 -138.302   <2e-16 ***
## sit.std     -0.07124    0.04619   -1.542    0.123    
## ---
## Signif. codes:  0 '***' 0.001 '**' 0.01 '*' 0.05 '.' 0.1 ' ' 1
## 
## (Dispersion parameter for binomial family taken to be 1)
## 
##     Null deviance: 6946.7  on 284164  degrees of freedom
## Residual deviance: 6944.3  on 284163  degrees of freedom
## AIC: 6948.3
## 
## Number of Fisher Scoring iterations: 9
```

```
## 
## Call:
## glm(formula = lsf ~ weight.std, family = binomial(link = "logit"), 
##     data = data)
## 
## Deviance Residuals: 
##     Min       1Q   Median       3Q      Max  
## -0.0582  -0.0576  -0.0575  -0.0574   3.5832  
## 
## Coefficients:
##              Estimate Std. Error  z value Pr(>|z|)    
## (Intercept) -6.405069   0.046214 -138.595   <2e-16 ***
## weight.std  -0.005128   0.046213   -0.111    0.912    
## ---
## Signif. codes:  0 '***' 0.001 '**' 0.01 '*' 0.05 '.' 0.1 ' ' 1
## 
## (Dispersion parameter for binomial family taken to be 1)
## 
##     Null deviance: 6946.7  on 284164  degrees of freedom
## Residual deviance: 6946.7  on 284163  degrees of freedom
## AIC: 6950.7
## 
## Number of Fisher Scoring iterations: 9
```

```
## 
## Call:
## glm(formula = lsf ~ bmi.std, family = binomial(link = "logit"), 
##     data = data)
## 
## Deviance Residuals: 
##     Min       1Q   Median       3Q      Max  
## -0.0587  -0.0577  -0.0575  -0.0573   3.5868  
## 
## Coefficients:
##              Estimate Std. Error z value Pr(>|z|)    
## (Intercept) -6.405099   0.046216  -138.6   <2e-16 ***
## bmi.std      0.009265   0.046214     0.2    0.841    
## ---
## Signif. codes:  0 '***' 0.001 '**' 0.01 '*' 0.05 '.' 0.1 ' ' 1
## 
## (Dispersion parameter for binomial family taken to be 1)
## 
##     Null deviance: 6946.7  on 284164  degrees of freedom
## Residual deviance: 6946.7  on 284163  degrees of freedom
## AIC: 6950.7
## 
## Number of Fisher Scoring iterations: 9
```

```
## 
## Call:
## glm(formula = cad ~ height.std, family = binomial(link = "logit"), 
##     data = data)
## 
## Deviance Residuals: 
##     Min       1Q   Median       3Q      Max  
## -0.2900  -0.2873  -0.2868  -0.2863   2.5429  
## 
## Coefficients:
##              Estimate Std. Error  z value Pr(>|z|)    
## (Intercept) -3.170268   0.009539 -332.353   <2e-16 ***
## height.std   0.005197   0.009539    0.545    0.586    
## ---
## Signif. codes:  0 '***' 0.001 '**' 0.01 '*' 0.05 '.' 0.1 ' ' 1
## 
## (Dispersion parameter for binomial family taken to be 1)
## 
##     Null deviance: 95990  on 284164  degrees of freedom
## Residual deviance: 95990  on 284163  degrees of freedom
## AIC: 95994
## 
## Number of Fisher Scoring iterations: 6
```

```
## 
## Call:
## glm(formula = cad ~ sit.std, family = binomial(link = "logit"), 
##     data = data)
## 
## Deviance Residuals: 
##     Min       1Q   Median       3Q      Max  
## -0.2915  -0.2874  -0.2868  -0.2861   2.5434  
## 
## Coefficients:
##              Estimate Std. Error  z value Pr(>|z|)    
## (Intercept) -3.170276   0.009539 -332.351   <2e-16 ***
## sit.std      0.006668   0.009539    0.699    0.485    
## ---
## Signif. codes:  0 '***' 0.001 '**' 0.01 '*' 0.05 '.' 0.1 ' ' 1
## 
## (Dispersion parameter for binomial family taken to be 1)
## 
##     Null deviance: 95990  on 284164  degrees of freedom
## Residual deviance: 95989  on 284163  degrees of freedom
## AIC: 95993
## 
## Number of Fisher Scoring iterations: 6
```

```
## 
## Call:
## glm(formula = cad ~ weight.std, family = binomial(link = "logit"), 
##     data = data)
## 
## Deviance Residuals: 
##     Min       1Q   Median       3Q      Max  
## -0.2952  -0.2879  -0.2867  -0.2855   2.5527  
## 
## Coefficients:
##              Estimate Std. Error  z value Pr(>|z|)    
## (Intercept) -3.170326   0.009539 -332.340   <2e-16 ***
## weight.std   0.012384   0.009539    1.298    0.194    
## ---
## Signif. codes:  0 '***' 0.001 '**' 0.01 '*' 0.05 '.' 0.1 ' ' 1
## 
## (Dispersion parameter for binomial family taken to be 1)
## 
##     Null deviance: 95990  on 284164  degrees of freedom
## Residual deviance: 95988  on 284163  degrees of freedom
## AIC: 95992
## 
## Number of Fisher Scoring iterations: 6
```

```
## 
## Call:
## glm(formula = cad ~ bmi.std, family = binomial(link = "logit"), 
##     data = data)
## 
## Deviance Residuals: 
##     Min       1Q   Median       3Q      Max  
## -0.2965  -0.2881  -0.2867  -0.2852   2.5562  
## 
## Coefficients:
##              Estimate Std. Error  z value Pr(>|z|)    
## (Intercept) -3.170353   0.009540 -332.334   <2e-16 ***
## bmi.std      0.014571   0.009539    1.527    0.127    
## ---
## Signif. codes:  0 '***' 0.001 '**' 0.01 '*' 0.05 '.' 0.1 ' ' 1
## 
## (Dispersion parameter for binomial family taken to be 1)
## 
##     Null deviance: 95990  on 284164  degrees of freedom
## Residual deviance: 95988  on 284163  degrees of freedom
## AIC: 95992
## 
## Number of Fisher Scoring iterations: 6
```

```
## 
## Call:
## glm(formula = alz ~ height.std, family = binomial(link = "logit"), 
##     data = data)
## 
## Deviance Residuals: 
##     Min       1Q   Median       3Q      Max  
## -0.1210  -0.1136  -0.1123  -0.1110   3.2148  
## 
## Coefficients:
##             Estimate Std. Error z value Pr(>|z|)    
## (Intercept) -5.06216    0.02373 -213.31   <2e-16 ***
## height.std  -0.03416    0.02372   -1.44     0.15    
## ---
## Signif. codes:  0 '***' 0.001 '**' 0.01 '*' 0.05 '.' 0.1 ' ' 1
## 
## (Dispersion parameter for binomial family taken to be 1)
## 
##     Null deviance: 21700  on 284164  degrees of freedom
## Residual deviance: 21698  on 284163  degrees of freedom
## AIC: 21702
## 
## Number of Fisher Scoring iterations: 8
```

```
## 
## Call:
## glm(formula = alz ~ sit.std, family = binomial(link = "logit"), 
##     data = data)
## 
## Deviance Residuals: 
##     Min       1Q   Median       3Q      Max  
## -0.1196  -0.1134  -0.1124  -0.1114   3.2082  
## 
## Coefficients:
##             Estimate Std. Error  z value Pr(>|z|)    
## (Intercept) -5.06192    0.02373 -213.355   <2e-16 ***
## sit.std     -0.02628    0.02371   -1.108    0.268    
## ---
## Signif. codes:  0 '***' 0.001 '**' 0.01 '*' 0.05 '.' 0.1 ' ' 1
## 
## (Dispersion parameter for binomial family taken to be 1)
## 
##     Null deviance: 21700  on 284164  degrees of freedom
## Residual deviance: 21698  on 284163  degrees of freedom
## AIC: 21702
## 
## Number of Fisher Scoring iterations: 8
```

```
## 
## Call:
## glm(formula = alz ~ weight.std, family = binomial(link = "logit"), 
##     data = data)
## 
## Deviance Residuals: 
##     Min       1Q   Median       3Q      Max  
## -0.1188  -0.1133  -0.1124  -0.1115   3.2052  
## 
## Coefficients:
##             Estimate Std. Error  z value Pr(>|z|)    
## (Intercept) -5.06186    0.02372 -213.366   <2e-16 ***
## weight.std  -0.02370    0.02372   -0.999    0.318    
## ---
## Signif. codes:  0 '***' 0.001 '**' 0.01 '*' 0.05 '.' 0.1 ' ' 1
## 
## (Dispersion parameter for binomial family taken to be 1)
## 
##     Null deviance: 21700  on 284164  degrees of freedom
## Residual deviance: 21699  on 284163  degrees of freedom
## AIC: 21703
## 
## Number of Fisher Scoring iterations: 8
```

```
## 
## Call:
## glm(formula = alz ~ bmi.std, family = binomial(link = "logit"), 
##     data = data)
## 
## Deviance Residuals: 
##     Min       1Q   Median       3Q      Max  
## -0.1162  -0.1130  -0.1124  -0.1118   3.1973  
## 
## Coefficients:
##             Estimate Std. Error  z value Pr(>|z|)    
## (Intercept) -5.06171    0.02372 -213.392   <2e-16 ***
## bmi.std     -0.01580    0.02372   -0.666    0.505    
## ---
## Signif. codes:  0 '***' 0.001 '**' 0.01 '*' 0.05 '.' 0.1 ' ' 1
## 
## (Dispersion parameter for binomial family taken to be 1)
## 
##     Null deviance: 21700  on 284164  degrees of freedom
## Residual deviance: 21699  on 284163  degrees of freedom
## AIC: 21703
## 
## Number of Fisher Scoring iterations: 8
```
