# Supplementary material for: Genetic influence on within-person longitudinal change in anthropometric traits in the UK Biobank
Source: Nat Commun. 2024 May 6;15:3776. doi: 10.1038/s41467-024-47802-7 (PMC11074304; doi:10.1038/s41467-024-47802-7)
Supplement: Supplementary file 6 — Source Data [file 41467_2024_47802_MOESM6_ESM.zip › data/2_longitudinal/longitudinalWithinPerson.html]

Within Person longditudinal analysis - UKBiobank


# Within Person longditudinal analysis - UKBiobank

#### by *Kathryn Kemper* - 12 March 2024

# Introduction & approach

This analysis uses data from 50,117 individuals with exactly 2
records of height, weight, bmi and sitting height.

The analysis follows similar methodology to that presented in: J D
Sorkin, D C Muller, R Andres, “Longitudinal change in height of men and
women: implications for interpretation of the body mass index: the
Baltimore Longitudinal Study of Aging” Am J Epidemiol 1999 Nov
1;150(9):969-77.

# 1. Summary of the data by age at recruitment

```
   # trait rate-change previously calculated as (y2-y1)/(age2-age1)
   RM2$height.mean = (RM2$height.1 + RM2$height.2 )/2
   RM2$weight.mean = (RM2$weight.1 + RM2$weight.2 )/2
   RM2$bmi.mean = (RM2$bmi.1 + RM2$bmi.2 )/2
   RM2$sit.mean = (RM2$sit.1 + RM2$sit.2 )/2
   RM2$age.mean = RM2$ageBase + 0.5*RM2$dAge
   RM2$ageBracket = cut_number(RM2$age.mean,10)
   
   #summarise data
   table = RM2 %>%
      group_by(sex,bracket=as.factor(ageBracket)) %>%
      dplyr::summarize(
                          n=sum(!is.na(height.rate)),
              avgAge = round(mean(age.mean),1),
              avgFollowUp = round(mean(dAge),2),
              avgHeight = round(mean(height.mean,na.rm=T),1),
              seHeight = round(standard_error(height.mean),3),
              avgHeightRate = round(mean(height.rate,na.rm=T),3),
              seHeightRate = round(standard_error(height.rate),3),
              avgWeight = round(mean(weight.mean,na.rm=T),1),
              seWeight = round(standard_error(weight.mean),3),
              avgWeightRate = round(mean(weight.rate,na.rm=T),3),
              seWeightRate = round(standard_error(weight.rate),3),
              avgBmi = round(mean(bmi.mean,na.rm=T),1),
              seBmi = round(standard_error(bmi.mean),3),
              avgBmiRate = round(mean(bmi.rate,na.rm=T),3),
              seBmiRate = round(standard_error(bmi.rate),3),
              avgSit = round(mean(sit.mean,na.rm=T),1),
              seSit = round(standard_error(sit.mean),3),
              avgSitRate = round(mean(sit.rate,na.rm=T),3),
              seSitRate = round(standard_error(sit.rate),3),
         )
```

```
## `summarise()` has grouped output by 'sex'. You can override using the `.groups`
## argument.
```

```
kable(table,caption="Trait averages & within-person change in recrutment age brackets")
```

Trait averages & within-person change in recrutment age
brackets


| sex | bracket | n | avgAge | avgFollowUp | avgHeight | seHeight | avgHeightRate | seHeightRate | avgWeight | seWeight | avgWeightRate | seWeightRate | avgBmi | seBmi | avgBmiRate | seBmiRate | avgSit | seSit | avgSitRate | seSitRate |
| --- | --- | --- | --- | --- | --- | --- | --- | --- | --- | --- | --- | --- | --- | --- | --- | --- | --- | --- | --- | --- |
| F | [41.5,48.6] | 2746 | 46.4 | 7.21 | 165.2 | 0.119 | -0.035 | 0.003 | 70.5 | 0.258 | 0.182 | 0.018 | 25.8 | 0.091 | 0.078 | 0.007 | 88.0 | 0.061 | -0.058 | 0.005 |
| F | (48.6,52.4] | 2799 | 50.6 | 7.93 | 164.5 | 0.115 | -0.044 | 0.003 | 70.3 | 0.255 | 0.119 | 0.015 | 26.0 | 0.092 | 0.058 | 0.006 | 87.8 | 0.060 | -0.074 | 0.004 |
| F | (52.4,55.5] | 2858 | 54.0 | 7.78 | 163.9 | 0.113 | -0.068 | 0.003 | 70.4 | 0.243 | 0.019 | 0.016 | 26.2 | 0.089 | 0.030 | 0.006 | 87.4 | 0.057 | -0.086 | 0.005 |
| F | (55.5,58.2] | 2762 | 56.8 | 7.63 | 163.5 | 0.115 | -0.084 | 0.003 | 70.2 | 0.248 | -0.060 | 0.015 | 26.3 | 0.090 | 0.006 | 0.006 | 87.1 | 0.059 | -0.110 | 0.005 |
| F | (58.2,60.5] | 2755 | 59.4 | 7.44 | 163.0 | 0.115 | -0.099 | 0.003 | 69.6 | 0.236 | -0.098 | 0.016 | 26.2 | 0.086 | -0.005 | 0.006 | 86.7 | 0.060 | -0.125 | 0.005 |
| F | (60.5,62.6] | 2577 | 61.6 | 7.23 | 162.6 | 0.119 | -0.113 | 0.003 | 69.5 | 0.248 | -0.136 | 0.017 | 26.3 | 0.091 | -0.014 | 0.006 | 86.4 | 0.061 | -0.130 | 0.005 |
| F | (62.6,64.5] | 2545 | 63.5 | 7.18 | 162.3 | 0.117 | -0.126 | 0.004 | 69.1 | 0.242 | -0.163 | 0.016 | 26.2 | 0.090 | -0.020 | 0.006 | 86.2 | 0.061 | -0.147 | 0.005 |
| F | (64.5,66.5] | 2389 | 65.5 | 7.64 | 162.0 | 0.124 | -0.135 | 0.003 | 69.2 | 0.245 | -0.161 | 0.015 | 26.4 | 0.090 | -0.016 | 0.006 | 85.9 | 0.065 | -0.166 | 0.005 |
| F | (66.5,69.1] | 2310 | 67.6 | 7.76 | 161.6 | 0.120 | -0.139 | 0.003 | 68.7 | 0.248 | -0.181 | 0.015 | 26.3 | 0.092 | -0.023 | 0.006 | 85.5 | 0.063 | -0.170 | 0.005 |
| F | (69.1,75.4] | 2018 | 71.1 | 8.49 | 161.1 | 0.127 | -0.150 | 0.003 | 67.6 | 0.242 | -0.180 | 0.014 | 26.0 | 0.090 | -0.021 | 0.006 | 85.0 | 0.067 | -0.171 | 0.005 |
| M | [41.5,48.6] | 2263 | 46.3 | 7.14 | 178.1 | 0.137 | -0.035 | 0.004 | 86.2 | 0.297 | 0.067 | 0.019 | 27.1 | 0.084 | 0.032 | 0.006 | 93.8 | 0.074 | -0.057 | 0.007 |
| M | (48.6,52.4] | 2211 | 50.6 | 7.83 | 177.7 | 0.138 | -0.041 | 0.003 | 86.0 | 0.286 | 0.056 | 0.017 | 27.2 | 0.083 | 0.030 | 0.005 | 93.6 | 0.074 | -0.072 | 0.006 |
| M | (52.4,55.5] | 2148 | 54.0 | 7.85 | 177.1 | 0.142 | -0.056 | 0.004 | 85.6 | 0.292 | -0.044 | 0.017 | 27.3 | 0.086 | 0.003 | 0.005 | 93.3 | 0.074 | -0.074 | 0.006 |
| M | (55.5,58.2] | 2249 | 56.9 | 7.66 | 176.8 | 0.138 | -0.064 | 0.004 | 85.4 | 0.289 | -0.079 | 0.017 | 27.3 | 0.084 | -0.006 | 0.005 | 93.1 | 0.073 | -0.086 | 0.006 |
| M | (58.2,60.5] | 2251 | 59.4 | 7.33 | 176.4 | 0.133 | -0.089 | 0.004 | 84.9 | 0.273 | -0.118 | 0.017 | 27.3 | 0.082 | -0.010 | 0.006 | 92.8 | 0.070 | -0.116 | 0.006 |
| M | (60.5,62.6] | 2430 | 61.6 | 7.21 | 176.3 | 0.128 | -0.093 | 0.004 | 84.6 | 0.264 | -0.122 | 0.016 | 27.2 | 0.078 | -0.010 | 0.005 | 92.6 | 0.069 | -0.119 | 0.006 |
| M | (62.6,64.5] | 2464 | 63.5 | 7.08 | 175.6 | 0.128 | -0.103 | 0.004 | 84.4 | 0.258 | -0.127 | 0.016 | 27.4 | 0.075 | -0.008 | 0.005 | 92.2 | 0.067 | -0.135 | 0.006 |
| M | (64.5,66.5] | 2613 | 65.5 | 7.46 | 175.2 | 0.125 | -0.103 | 0.003 | 83.6 | 0.243 | -0.144 | 0.015 | 27.2 | 0.072 | -0.014 | 0.005 | 91.9 | 0.067 | -0.128 | 0.005 |
| M | (66.5,69.1] | 2696 | 67.7 | 7.58 | 175.2 | 0.119 | -0.120 | 0.003 | 83.3 | 0.233 | -0.146 | 0.014 | 27.1 | 0.071 | -0.010 | 0.005 | 91.7 | 0.062 | -0.138 | 0.005 |
| M | (69.1,75.4] | 2988 | 71.1 | 8.41 | 174.3 | 0.113 | -0.128 | 0.003 | 81.5 | 0.214 | -0.201 | 0.012 | 26.8 | 0.064 | -0.027 | 0.004 | 91.2 | 0.060 | -0.138 | 0.004 |

```
table$upperHeight=table$avgHeight+1.96*table$seHeight ; table$lowerHeight=table$avgHeight-1.96*table$seHeight
table$upperHeightRate=table$avgHeightRate+1.96*table$seHeightRate ; table$lowerHeightRate=table$avgHeightRate-1.96*table$seHeightRate
table$upperWeight=table$avgWeight+1.96*table$seWeight ; table$lowerWeight=table$avgWeight-1.96*table$seWeight
table$upperWeightRate=table$avgWeightRate+1.96*table$seWeightRate ; table$lowerWeightRate=table$avgWeightRate-1.96*table$seWeightRate
table$upperBmi=table$avgBmi+1.96*table$seBmi ; table$lowerBmi=table$avgBmi-1.96*table$seBmi
table$upperBmiRate=table$avgBmiRate+1.96*table$seBmiRate ; table$lowerBmiRate=table$avgBmiRate-1.96*table$seBmiRate
table$upperSit=table$avgSit+1.96*table$seSit ; table$lowerSit=table$avgSit-1.96*table$seSit
table$upperSitRate=table$avgSitRate+1.96*table$seSitRate ; table$lowerSitRate=table$avgSitRate-1.96*table$seSitRate
```

## 2. Cross-sectional height & rate of height change

```
   aggregate(RM2$age.mean~RM2$sex,FUN=mean)
```

```
##   RM2$sex RM2$age.mean
## 1       F     59.02172
## 2       M     60.32496
```

```
   RM2$age.adj=RM2$age.mean-59.02172                    #females
   RM2$age.adj[RM2$sex=="M"]=RM2$age.mean[RM2$sex=="M"]-60.32496    #males
   RM2$age.adj2=RM2$age.adj^2

   summary(lm(height.mean~age.adj*sex+age.adj2*sex,data=RM2)) #testing terms
```

```
## 
## Call:
## lm(formula = height.mean ~ age.adj * sex + age.adj2 * sex, data = RM2)
## 
## Residuals:
##      Min       1Q   Median       3Q      Max 
## -24.7179  -4.1809  -0.0538   4.1035  29.6040 
## 
## Coefficients:
##                 Estimate Std. Error  t value Pr(>|t|)    
## (Intercept)    1.631e+02  5.276e-02 3091.034   <2e-16 ***
## age.adj       -1.662e-01  5.331e-03  -31.165   <2e-16 ***
## sexM           1.317e+01  7.597e-02  173.409   <2e-16 ***
## age.adj2      -1.986e-05  6.698e-04   -0.030   0.9763    
## age.adj:sexM   1.105e-02  7.685e-03    1.438   0.1506    
## sexM:age.adj2 -1.608e-03  9.392e-04   -1.712   0.0868 .  
## ---
## Signif. codes:  0 '***' 0.001 '**' 0.01 '*' 0.05 '.' 0.1 ' ' 1
## 
## Residual standard error: 6.186 on 50066 degrees of freedom
##   (45 observations deleted due to missingness)
## Multiple R-squared:  0.5357, Adjusted R-squared:  0.5357 
## F-statistic: 1.155e+04 on 5 and 50066 DF,  p-value: < 2.2e-16
```

```
   summary(lm(height.rate~age.adj*sex+age.adj2*sex,data=RM2)) #testing terms
```

```
## 
## Call:
## lm(formula = height.rate ~ age.adj * sex + age.adj2 * sex, data = RM2)
## 
## Residuals:
##      Min       1Q   Median       3Q      Max 
## -1.35346 -0.08773  0.02034  0.09588  1.62734 
## 
## Coefficients:
##                 Estimate Std. Error t value Pr(>|t|)    
## (Intercept)   -9.689e-02  1.423e-03 -68.102  < 2e-16 ***
## age.adj       -5.126e-03  1.438e-04 -35.653  < 2e-16 ***
## sexM           1.159e-02  2.049e-03   5.659 1.53e-08 ***
## age.adj2       1.329e-05  1.806e-05   0.736    0.462    
## age.adj:sexM   1.093e-03  2.073e-04   5.272 1.35e-07 ***
## sexM:age.adj2 -2.639e-05  2.533e-05  -1.042    0.297    
## ---
## Signif. codes:  0 '***' 0.001 '**' 0.01 '*' 0.05 '.' 0.1 ' ' 1
## 
## Residual standard error: 0.1668 on 50066 degrees of freedom
##   (45 observations deleted due to missingness)
## Multiple R-squared:  0.0414, Adjusted R-squared:  0.0413 
## F-statistic: 432.4 on 5 and 50066 DF,  p-value: < 2.2e-16
```

```
   lmHeightA=lm(height.mean~-1+sex+age.adj:sex,data=RM2)
   lmHeightR=lm(height.rate~-1+sex+age.adj:sex,data=RM2)
   summary(lmHeightA) #final model
```

```
## 
## Call:
## lm(formula = height.mean ~ -1 + sex + age.adj:sex, data = RM2)
## 
## Residuals:
##      Min       1Q   Median       3Q      Max 
## -24.7652  -4.1807  -0.0553   4.1049  29.6048 
## 
## Coefficients:
##                Estimate Std. Error t value Pr(>|t|)    
## sexF         163.068735   0.038544 4230.71   <2e-16 ***
## sexM         176.150288   0.039674 4439.98   <2e-16 ***
## sexF:age.adj  -0.166128   0.005256  -31.61   <2e-16 ***
## sexM:age.adj  -0.150765   0.005250  -28.72   <2e-16 ***
## ---
## Signif. codes:  0 '***' 0.001 '**' 0.01 '*' 0.05 '.' 0.1 ' ' 1
## 
## Residual standard error: 6.186 on 50068 degrees of freedom
##   (45 observations deleted due to missingness)
## Multiple R-squared:  0.9987, Adjusted R-squared:  0.9987 
## F-statistic: 9.404e+06 on 4 and 50068 DF,  p-value: < 2.2e-16
```

```
   summary(lmHeightR) #final model
```

```
## 
## Call:
## lm(formula = height.rate ~ -1 + sex + age.adj:sex, data = RM2)
## 
## Residuals:
##      Min       1Q   Median       3Q      Max 
## -1.35397 -0.08777  0.02042  0.09600  1.62709 
## 
## Coefficients:
##                Estimate Std. Error t value Pr(>|t|)    
## sexF         -0.0961784  0.0010394  -92.53   <2e-16 ***
## sexM         -0.0860481  0.0010699  -80.43   <2e-16 ***
## sexF:age.adj -0.0051440  0.0001417  -36.29   <2e-16 ***
## sexM:age.adj -0.0039985  0.0001416  -28.24   <2e-16 ***
## ---
## Signif. codes:  0 '***' 0.001 '**' 0.01 '*' 0.05 '.' 0.1 ' ' 1
## 
## Residual standard error: 0.1668 on 50068 degrees of freedom
##   (45 observations deleted due to missingness)
## Multiple R-squared:  0.2551, Adjusted R-squared:  0.255 
## F-statistic:  4286 on 4 and 50068 DF,  p-value: < 2.2e-16
```

```
   avgAgeAdj=aggregate(RM2$age.adj~RM2$ageBracket+RM2$sex,FUN=mean)
   colFM=c("dark grey","orange")
   cols=c(rep(colFM[1],10),rep(colFM[2],10))
   par(mfrow=c(1,2))
    { plot(table$avgHeight~avgAgeAdj[,3],col=cols,cex=2,pch=20,
        xlim=c(-15,15),xlab="Sex-centred average age, years",
        ylab="average height, cm",las=1)
   segments(x0=avgAgeAdj[,3],y0=table$upperHeight,y1=table$lowerHeight,col=cols)
   abline(a=lmHeightA$coefficients[1],b=lmHeightA$coefficients[3],col=colFM[1],lwd=2)
   abline(a=lmHeightA$coefficients[2],b=lmHeightA$coefficients[4],col=colFM[2],lwd=2) 
   legend("right",legend=c("females","males"),fill=colFM) }
   { plot(table$avgHeightRate~avgAgeAdj[,3],col=cols,cex=2,pch=20,las=1, 
    xlim=c(-15,15),xlab="Sex-centred average age, years", 
    ylab="height change, cm/yr",las=1,
    ylim=range(c(table$upperHeightRate,table$lowerHeightRate)))
   abline(a=lmHeightR$coefficients[1],b=lmHeightR$coefficients[3],col=colFM[1],lwd=2)
   segments(x0=avgAgeAdj[,3],y0=table$upperHeightRate,y1=table$lowerHeightRate,col=cols)
   abline(a=lmHeightR$coefficients[2],b=lmHeightR$coefficients[4],col=colFM[2],lwd=2)
   legend("topright",legend=c("females","males"),fill=colFM) }
```

```
#   write.table(file="height_ageCorrected.dat",data.frame(ID=RM2$ID[!is.na(RM2$height.mean)],ID2=RM2$ID[!is.na(RM2$height.mean)],mean=residuals(lmHeightA),rate=residuals(lmHeightR)),row.names=FALSE,col.names=FALSE,quote=FALSE)
```

### intergrating within-person longitudinal change to estimate cumulative height change

```
   fHeightChange = function(age) { 
       lmHeightR$coefficients[1]*age + 0.5*lmHeightR$coefficients[3]*age^2 }
   mHeightChange = function(age) {
       lmHeightR$coefficients[2]*age + 0.5*lmHeightR$coefficients[4]*age^2 }
   # 
   relAge = seq(-15,15)
   { plot(fHeightChange(relAge)~relAge,col=colFM[1],lwd=2,type="l",
        xlab="Sex-centred average age, years (relative ~60 years)",
        ylab="height change, cm")
   points(mHeightChange(relAge)~relAge,col=colFM[2],lwd=2,type="l",lty=2)
   legend("topright",legend=c("females","males"),fill=colFM) }
```

## 3. Cross-sectional sitting height & rate of sitting height change

```
   summary(lm(sit.mean~age.adj*sex+age.adj2*sex,data=RM2)) #testing
```

```
## 
## Call:
## lm(formula = sit.mean ~ age.adj * sex + age.adj2 * sex, data = RM2)
## 
## Residuals:
##      Min       1Q   Median       3Q      Max 
## -17.4740  -2.1634  -0.0287   2.1298  16.2058 
## 
## Coefficients:
##                 Estimate Std. Error  t value Pr(>|t|)    
## (Intercept)   86.7887991  0.0276678 3136.820  < 2e-16 ***
## age.adj       -0.1266304  0.0027932  -45.335  < 2e-16 ***
## sexM           5.8922962  0.0398231  147.962  < 2e-16 ***
## age.adj2      -0.0020318  0.0003511   -5.787 7.22e-09 ***
## age.adj:sexM   0.0112610  0.0040259    2.797  0.00516 ** 
## sexM:age.adj2 -0.0003706  0.0004923   -0.753  0.45157    
## ---
## Signif. codes:  0 '***' 0.001 '**' 0.01 '*' 0.05 '.' 0.1 ' ' 1
## 
## Residual standard error: 3.229 on 49643 degrees of freedom
##   (468 observations deleted due to missingness)
## Multiple R-squared:  0.4734, Adjusted R-squared:  0.4733 
## F-statistic:  8925 on 5 and 49643 DF,  p-value: < 2.2e-16
```

```
   summary(lm(sit.rate~age.adj*sex+age.adj2*sex,data=RM2)) #testing
```

```
## 
## Call:
## lm(formula = sit.rate ~ age.adj * sex + age.adj2 * sex, data = RM2)
## 
## Residuals:
##      Min       1Q   Median       3Q      Max 
## -2.87138 -0.12124  0.01939  0.13153  2.61722 
## 
## Coefficients:
##                 Estimate Std. Error t value Pr(>|t|)    
## (Intercept)   -1.210e-01  2.243e-03 -53.953  < 2e-16 ***
## age.adj       -5.177e-03  2.264e-04 -22.865  < 2e-16 ***
## sexM           8.024e-03  3.228e-03   2.486   0.0129 *  
## age.adj2       1.047e-05  2.846e-05   0.368   0.7130    
## age.adj:sexM   1.771e-03  3.263e-04   5.427 5.75e-08 ***
## sexM:age.adj2  6.630e-05  3.990e-05   1.662   0.0966 .  
## ---
## Signif. codes:  0 '***' 0.001 '**' 0.01 '*' 0.05 '.' 0.1 ' ' 1
## 
## Residual standard error: 0.2617 on 49643 degrees of freedom
##   (468 observations deleted due to missingness)
## Multiple R-squared:  0.01656,    Adjusted R-squared:  0.01646 
## F-statistic: 167.2 on 5 and 49643 DF,  p-value: < 2.2e-16
```

```
   lmSitA=lm(sit.mean~-1+sex+age.adj:sex+age.adj2,data=RM2)
   lmSitR=lm(sit.rate~-1+sex+age.adj:sex,data=RM2)
   summary(lmSitA) #final
```

```
## 
## Call:
## lm(formula = sit.mean ~ -1 + sex + age.adj:sex + age.adj2, data = RM2)
## 
## Residuals:
##      Min       1Q   Median       3Q      Max 
## -17.4762  -2.1611  -0.0288   2.1271  16.2124 
## 
## Coefficients:
##                Estimate Std. Error  t value Pr(>|t|)    
## sexF         86.7989464  0.0241620 3592.367   <2e-16 ***
## sexM         92.6706985  0.0250923 3693.189   <2e-16 ***
## age.adj2     -0.0022203  0.0002461   -9.022   <2e-16 ***
## sexF:age.adj -0.1268779  0.0027738  -45.742   <2e-16 ***
## sexM:age.adj -0.1148858  0.0028272  -40.636   <2e-16 ***
## ---
## Signif. codes:  0 '***' 0.001 '**' 0.01 '*' 0.05 '.' 0.1 ' ' 1
## 
## Residual standard error: 3.229 on 49644 degrees of freedom
##   (468 observations deleted due to missingness)
## Multiple R-squared:  0.9987, Adjusted R-squared:  0.9987 
## F-statistic: 7.644e+06 on 5 and 49644 DF,  p-value: < 2.2e-16
```

```
   summary(lmSitR) #final
```

```
## 
## Call:
## lm(formula = sit.rate ~ -1 + sex + age.adj:sex, data = RM2)
## 
## Residuals:
##     Min      1Q  Median      3Q     Max 
## -2.8757 -0.1214  0.0186  0.1315  2.6292 
## 
## Coefficients:
##                Estimate Std. Error t value Pr(>|t|)    
## sexF         -0.1204289  0.0016380  -73.52   <2e-16 ***
## sexM         -0.1085845  0.0016850  -64.44   <2e-16 ***
## sexF:age.adj -0.0051904  0.0002233  -23.24   <2e-16 ***
## sexM:age.adj -0.0036096  0.0002230  -16.19   <2e-16 ***
## ---
## Signif. codes:  0 '***' 0.001 '**' 0.01 '*' 0.05 '.' 0.1 ' ' 1
## 
## Residual standard error: 0.2617 on 49645 degrees of freedom
##   (468 observations deleted due to missingness)
## Multiple R-squared:  0.1728, Adjusted R-squared:  0.1727 
## F-statistic:  2592 on 4 and 49645 DF,  p-value: < 2.2e-16
```

```
   par(mfrow=c(1,2))
    { plot(table$avgSit~avgAgeAdj[,3],col=cols,pch=20,cex=2,las=1,
        xlim=c(-15,15),xlab="Sex-centred average age, years (relative ~60 years)",
        ylab="average sitting height, cm")
   segments(x0=avgAgeAdj[,3],y0=table$upperSit,y1=table$lowerSit,col=cols)
   predictF=predict.lm(lmSitA,
           newdata=data.frame(age.adj=relAge,age.adj2=relAge^2,sex="F"))
   points(relAge,predictF,col=colFM[1],type="l",lty=2)
   predictM=predict.lm(lmSitA,
               newdata=data.frame(age.adj=relAge,age.adj2=relAge^2,sex="M"))
   points(relAge,predictM,col=colFM[2],type="l",lty=2)
   legend("right",legend=c("females","males"),fill=colFM) }
    { plot(table$avgSitRate~avgAgeAdj[,3],col=cols,pch=20,cex=2,las=1,
        xlim=c(-15,15),xlab="Sex-centred average age, years",
        ylab="sitting height change, cm/yr",
    ylim=range(c(table$upperSitRate,table$lowerSitRate)))
   segments(x0=avgAgeAdj[,3],y0=table$upperSitRate,y1=table$lowerSitRate,col=cols)
   abline(a=lmSitR$coefficients[1],b=lmSitR$coefficients[3],col=colFM[1],lty=2)
   abline(a=lmSitR$coefficients[2],b=lmSitR$coefficients[4],col=colFM[2],lty=2)
   legend("topright",legend=c("females","males"),fill=colFM) }
```

```
 #  write.table(file="sit_ageCorrected.dat",data.frame(ID=RM2$ID[!is.na(RM2$sit.mean)],ID2=RM2$ID[!is.na(RM2$sit.mean)],mean=residuals(lmSitA),rate=residuals(lmSitR)),row.names=FALSE,col.names=FALSE,quote=FALSE)
```

### intergrating within-person longitudinal change to estimate cumulative sitting height change

```
   fSitChange = function(age) {
           lmSitR$coefficients[1]*age + 0.5*lmSitR$coefficients[3]*age^2 }
   mSitChange = function(age) {
           lmSitR$coefficients[2]*age + 0.5*lmSitR$coefficients[4]*age^2 }
   # 
   plot(fSitChange(relAge)~relAge,col=colFM[1],lwd=2,type="l",
        xlab="relative age, years",
        ylab="relative sitting height, cm",las=1)
   points(mSitChange(relAge)~relAge,col=colFM[2],lwd=2,type="l",lty=2)
   legend("topright",legend=c("females","males"),fill=colFM)
```

## 5. Cross-sectional bmi change & rate of bmi change

```
   summary(lm(bmi.mean~age.adj*sex+age.adj2*sex,data=RM2))
```

```
## 
## Call:
## lm(formula = bmi.mean ~ age.adj * sex + age.adj2 * sex, data = RM2)
## 
## Residuals:
##     Min      1Q  Median      3Q     Max 
## -11.927  -2.902  -0.641   2.141  35.199 
## 
## Coefficients:
##                 Estimate Std. Error t value Pr(>|t|)    
## (Intercept)    2.629e+01  3.598e-02 730.641  < 2e-16 ***
## age.adj        1.100e-02  3.640e-03   3.022  0.00251 ** 
## sexM           1.031e+00  5.182e-02  19.890  < 2e-16 ***
## age.adj2      -2.215e-03  4.572e-04  -4.844 1.28e-06 ***
## age.adj:sexM  -2.811e-02  5.246e-03  -5.359 8.40e-08 ***
## sexM:age.adj2 -8.546e-05  6.410e-04  -0.133  0.89393    
## ---
## Signif. codes:  0 '***' 0.001 '**' 0.01 '*' 0.05 '.' 0.1 ' ' 1
## 
## Residual standard error: 4.216 on 49962 degrees of freedom
##   (149 observations deleted due to missingness)
## Multiple R-squared:  0.01581,    Adjusted R-squared:  0.01571 
## F-statistic: 160.5 on 5 and 49962 DF,  p-value: < 2.2e-16
```

```
   summary(lm(bmi.rate~age.adj*sex+age.adj2*sex,data=RM2))
```

```
## 
## Call:
## lm(formula = bmi.rate ~ age.adj * sex + age.adj2 * sex, data = RM2)
## 
## Residuals:
##      Min       1Q   Median       3Q      Max 
## -2.71736 -0.13122  0.00062  0.13754  2.54307 
## 
## Coefficients:
##                 Estimate Std. Error t value Pr(>|t|)    
## (Intercept)    1.492e-04  2.391e-03   0.062  0.95024    
## age.adj       -4.246e-03  2.419e-04 -17.555  < 2e-16 ***
## sexM          -5.724e-03  3.443e-03  -1.662  0.09646 .  
## age.adj2       1.722e-04  3.038e-05   5.669 1.45e-08 ***
## age.adj:sexM   2.176e-03  3.486e-04   6.243 4.32e-10 ***
## sexM:age.adj2 -1.315e-04  4.259e-05  -3.087  0.00202 ** 
## ---
## Signif. codes:  0 '***' 0.001 '**' 0.01 '*' 0.05 '.' 0.1 ' ' 1
## 
## Residual standard error: 0.2801 on 49962 degrees of freedom
##   (149 observations deleted due to missingness)
## Multiple R-squared:  0.009833,   Adjusted R-squared:  0.009734 
## F-statistic: 99.23 on 5 and 49962 DF,  p-value: < 2.2e-16
```

```
   lmBmiA=lm(bmi.mean~-1+sex+age.adj:sex+age.adj2,data=RM2)
   lmBmiR=lm(bmi.rate~age.adj:sex+age.adj2:sex,data=RM2) 
   summary(lmBmiA)
```

```
## 
## Call:
## lm(formula = bmi.mean ~ -1 + sex + age.adj:sex + age.adj2, data = RM2)
## 
## Residuals:
##     Min      1Q  Median      3Q     Max 
## -11.928  -2.903  -0.641   2.140  35.201 
## 
## Coefficients:
##                Estimate Std. Error t value Pr(>|t|)    
## sexF         26.2930348  0.0314288 836.590  < 2e-16 ***
## sexM         27.3188988  0.0326659 836.312  < 2e-16 ***
## age.adj2     -0.0022584  0.0003204  -7.048 1.84e-12 ***
## sexF:age.adj  0.0109409  0.0036132   3.028  0.00246 ** 
## sexM:age.adj -0.0170002  0.0036823  -4.617 3.91e-06 ***
## ---
## Signif. codes:  0 '***' 0.001 '**' 0.01 '*' 0.05 '.' 0.1 ' ' 1
## 
## Residual standard error: 4.216 on 49963 degrees of freedom
##   (149 observations deleted due to missingness)
## Multiple R-squared:  0.9756, Adjusted R-squared:  0.9756 
## F-statistic: 4e+05 on 5 and 49963 DF,  p-value: < 2.2e-16
```

```
   summary(lmBmiR)
```

```
## 
## Call:
## lm(formula = bmi.rate ~ age.adj:sex + age.adj2:sex, data = RM2)
## 
## Residuals:
##      Min       1Q   Median       3Q      Max 
## -2.71757 -0.13114  0.00049  0.13747  2.54564 
## 
## Coefficients:
##                 Estimate Std. Error t value Pr(>|t|)    
## (Intercept)   -2.611e-03  1.721e-03  -1.518    0.129    
## age.adj:sexF  -4.214e-03  2.411e-04 -17.478  < 2e-16 ***
## age.adj:sexM  -2.135e-03  2.479e-04  -8.613  < 2e-16 ***
## sexF:age.adj2  1.962e-04  2.675e-05   7.333 2.28e-13 ***
## sexM:age.adj2  1.619e-05  2.594e-05   0.624    0.533    
## ---
## Signif. codes:  0 '***' 0.001 '**' 0.01 '*' 0.05 '.' 0.1 ' ' 1
## 
## Residual standard error: 0.2801 on 49963 degrees of freedom
##   (149 observations deleted due to missingness)
## Multiple R-squared:  0.009779,   Adjusted R-squared:  0.009699 
## F-statistic: 123.3 on 4 and 49963 DF,  p-value: < 2.2e-16
```

```
   par(mfrow=c(1,2))
    { plot(table$avgBmi~avgAgeAdj[,3],col=cols,pch=20,cex=2,las=1,
        xlim=c(-15,15),xlab="Sex-centred average age, years",
        ylab="average bmi, kg/cm2",
    ylim=range(c(table$upperBmi,table$lowerBmi)))
   segments(x0=avgAgeAdj[,3],y0=table$upperBmi,y1=table$lowerBmi,col=cols)
   predictF=predict.lm(lmBmiA,
               newdata=data.frame(age.adj=relAge,age.adj2=relAge^2,sex="F"))
   points(relAge,predictF,col=colFM[1],type="l",lty=2)
   predictM=predict.lm(lmBmiA,
               newdata=data.frame(age.adj=relAge,age.adj2=relAge^2,sex="M"))
   points(relAge,predictM,col=colFM[2],type="l",lty=2)
   legend("left",legend=c("females","males"),fill=colFM) }
    { plot(table$avgBmiRate~avgAgeAdj[,3],col=cols,pch=20,cex=2,las=1,
        xlim=c(-15,15),xlab="Sex-centred average age, years",
        ylab="BMI change, per yr",
    ylim=range(c(table$upperBmiRate,table$lowerBmiRate)))
   segments(x0=avgAgeAdj[,3],y0=table$upperBmiRate,y1=table$lowerBmiRate,col=cols)
   predictF=predict.lm(lmBmiR,
               newdata=data.frame(age.adj=relAge,age.adj2=relAge^2,sex="F"))
   points(relAge,predictF,col=colFM[1],type="l",lty=2)
   predictM=predict.lm(lmBmiR,
               newdata=data.frame(age.adj=relAge,age.adj2=relAge^2,sex="M"))
   points(relAge,predictM,col=colFM[2],type="l",lty=2)
   legend("topright",legend=c("females","males"),fill=colFM) }
```

```
#   write.table(file="bmi_ageCorrected.dat",data.frame(ID=RM2$ID[!is.na(RM2$bmi.mean)],ID2=RM2$ID[!is.na(RM2$bmi.mean)],mean=residuals(lmBmiA),rate=residuals(lmBmiR)),row.names=FALSE,col.names=FALSE,quote=FALSE)
```

### intergrating within-person longitudinal change to estimate cumulative bmi change

```
   fBmiChange = function(age) {
       lmBmiR$coefficients[1]*age + 0.5*lmBmiR$coefficients[2]*age^2 + (1/3)*lmBmiR$coefficients[4]*age^3 }
   mBmiChange = function(age) {
     lmBmiR$coefficients[1]*age + 0.5*lmBmiR$coefficients[3]*age^2 + (1/3)*lmBmiR$coefficients[5]*age^3 }

   plot(fBmiChange(relAge)~relAge,col=colFM[1],lwd=2,type="l",las=1,
        xlab="relative age, years",
        ylab="relative bmi, kg/cm2")
   points(mBmiChange(relAge)~relAge,col=colFM[2],lwd=2,type="l",lty=2)
   legend("topright",legend=c("females","males"),fill=colFM)
```

## 6. Cross-sectional weight change & rate of weight change

```
   summary(lm(weight.mean~age.adj*sex+age.adj2*sex,data=RM2)) #testing
```

```
## 
## Call:
## lm(formula = weight.mean ~ age.adj * sex + age.adj2 * sex, data = RM2)
## 
## Residuals:
##     Min      1Q  Median      3Q     Max 
## -37.506  -8.807  -1.723   6.827  99.016 
## 
## Coefficients:
##                Estimate Std. Error t value Pr(>|t|)    
## (Intercept)   69.885793   0.108745 642.655  < 2e-16 ***
## age.adj       -0.112379   0.011000 -10.216  < 2e-16 ***
## sexM          15.025722   0.156599  95.950  < 2e-16 ***
## age.adj2      -0.005740   0.001382  -4.154 3.27e-05 ***
## age.adj:sexM  -0.089869   0.015854  -5.669 1.45e-08 ***
## sexM:age.adj2 -0.002673   0.001937  -1.380    0.168    
## ---
## Signif. codes:  0 '***' 0.001 '**' 0.01 '*' 0.05 '.' 0.1 ' ' 1
## 
## Residual standard error: 12.74 on 49993 degrees of freedom
##   (118 observations deleted due to missingness)
## Multiple R-squared:  0.2581, Adjusted R-squared:  0.258 
## F-statistic:  3478 on 5 and 49993 DF,  p-value: < 2.2e-16
```

```
   summary(lm(weight.rate~age.adj*sex+age.adj2*sex,data=RM2)) #testing
```

```
## 
## Call:
## lm(formula = weight.rate ~ age.adj * sex + age.adj2 * sex, data = RM2)
## 
## Residuals:
##     Min      1Q  Median      3Q     Max 
## -7.1941 -0.3629  0.0159  0.3962  7.0774 
## 
## Coefficients:
##                 Estimate Std. Error t value Pr(>|t|)    
## (Intercept)   -8.542e-02  6.776e-03 -12.605  < 2e-16 ***
## age.adj       -1.573e-02  6.855e-04 -22.946  < 2e-16 ***
## sexM          -1.474e-02  9.758e-03  -1.511  0.13090    
## age.adj2       5.168e-04  8.611e-05   6.001 1.97e-09 ***
## age.adj:sexM   5.520e-03  9.879e-04   5.587 2.32e-08 ***
## sexM:age.adj2 -3.785e-04  1.207e-04  -3.135  0.00172 ** 
## ---
## Signif. codes:  0 '***' 0.001 '**' 0.01 '*' 0.05 '.' 0.1 ' ' 1
## 
## Residual standard error: 0.7942 on 49993 degrees of freedom
##   (118 observations deleted due to missingness)
## Multiple R-squared:  0.01767,    Adjusted R-squared:  0.01758 
## F-statistic: 179.9 on 5 and 49993 DF,  p-value: < 2.2e-16
```

```
   lmWeightA=lm(weight.mean~-1+sex+age.adj:sex+age.adj2,data=RM2)
   lmWeightR=lm(weight.rate~age.adj:sex+sex:age.adj2,data=RM2)
   summary(lmWeightA) #final
```

```
## 
## Call:
## lm(formula = weight.mean ~ -1 + sex + age.adj:sex + age.adj2, 
##     data = RM2)
## 
## Residuals:
##     Min      1Q  Median      3Q     Max 
## -37.780  -8.801  -1.718   6.829  99.070 
## 
## Coefficients:
##                Estimate Std. Error t value Pr(>|t|)    
## sexF         69.9588407  0.0949855 736.521  < 2e-16 ***
## sexM         84.8365686  0.0987256 859.317  < 2e-16 ***
## age.adj2     -0.0071001  0.0009685  -7.331 2.32e-13 ***
## sexF:age.adj -0.1142101  0.0109201 -10.459  < 2e-16 ***
## sexM:age.adj -0.1987390  0.0111297 -17.857  < 2e-16 ***
## ---
## Signif. codes:  0 '***' 0.001 '**' 0.01 '*' 0.05 '.' 0.1 ' ' 1
## 
## Residual standard error: 12.74 on 49994 degrees of freedom
##   (118 observations deleted due to missingness)
## Multiple R-squared:  0.9734, Adjusted R-squared:  0.9734 
## F-statistic: 3.665e+05 on 5 and 49994 DF,  p-value: < 2.2e-16
```

```
   summary(lmWeightR) #final
```

```
## 
## Call:
## lm(formula = weight.rate ~ age.adj:sex + sex:age.adj2, data = RM2)
## 
## Residuals:
##     Min      1Q  Median      3Q     Max 
## -7.1947 -0.3633  0.0154  0.3968  7.0768 
## 
## Coefficients:
##                 Estimate Std. Error t value Pr(>|t|)    
## (Intercept)   -9.253e-02  4.876e-03 -18.975  < 2e-16 ***
## age.adj:sexF  -1.565e-02  6.833e-04 -22.899  < 2e-16 ***
## age.adj:sexM  -1.038e-02  7.026e-04 -14.771  < 2e-16 ***
## sexF:age.adj2  5.784e-04  7.582e-05   7.629 2.41e-14 ***
## sexM:age.adj2  7.505e-05  7.353e-05   1.021    0.307    
## ---
## Signif. codes:  0 '***' 0.001 '**' 0.01 '*' 0.05 '.' 0.1 ' ' 1
## 
## Residual standard error: 0.7942 on 49994 degrees of freedom
##   (118 observations deleted due to missingness)
## Multiple R-squared:  0.01763,    Adjusted R-squared:  0.01755 
## F-statistic: 224.3 on 4 and 49994 DF,  p-value: < 2.2e-16
```

```
   par(mfrow=c(1,2))
    { plot(table$avgWeight~avgAgeAdj[,3],col=cols,pch=20,cex=2,las=1,
        xlim=c(-15,15),xlab="Sex-centred average age, years",
        ylab="average weight, kg")
   segments(x0=avgAgeAdj[,3],y0=table$upperWeight,y1=table$lowerWeight,col=cols)
   predictF=predict.lm(lmWeightA,
               newdata=data.frame(age.adj=relAge,age.adj2=relAge^2,sex="F"))
   points(relAge,predictF,col=colFM[1],type="l",lty=2)
   predictM=predict.lm(lmWeightA,
               newdata=data.frame(age.adj=relAge,age.adj2=relAge^2,sex="M"))
   points(relAge,predictM,col=colFM[2],type="l",lty=2)
   legend("topright",legend=c("females","males"),fill=colFM) }
    { plot(table$avgWeightRate~avgAgeAdj[,3],col=cols,pch=20,cex=2,las=1,
        xlim=c(-15,15),xlab="Sex-centred average age, years",
        ylab="weight change, kg/yr",
    ylim=range(c(table$upperWeightRate,table$lowerWeightRate)))
   segments(x0=avgAgeAdj[,3],y0=table$upperWeightRate,y1=table$lowerWeightRate,col=cols)
   predictF=predict.lm(lmWeightR,
               newdata=data.frame(age.adj=relAge,age.adj2=relAge^2,sex="F"))
   points(relAge,predictF,col=colFM[1],type="l",lty=2)
   predictM=predict.lm(lmWeightR,
               newdata=data.frame(age.adj=relAge,age.adj2=relAge^2,sex="M"))
   points(relAge,predictM,col=colFM[2],type="l",lty=2)
   legend("topright",legend=c("females","males"),fill=colFM ) }
```

```
 #  write.table(file="weight_ageCorrected.dat",data.frame(ID=RM2$ID[!is.na(RM2$weight.mean)],ID2=RM2$ID[!is.na(RM2$weight.mean)],mean=residuals(lmWeightA),rate=residuals(lmWeightR)),row.names=FALSE,col.names=FALSE,quote=FALSE)
```

### intergrating within-person longitudinal change to estimate cumulative weight change

```
   fWeightChange = function(age) {
     lmWeightR$coefficients[1]*age + (1/2)*lmWeightR$coefficients[2]*age^2 + (1/3)*lmWeightR$coefficients[4]*age^3 }
   mWeightChange = function(age) {
     lmWeightR$coefficients[1]*age + (1/2)*lmWeightR$coefficients[3]*age^2 + (1/3)*lmWeightR$coefficients[5]*age^3 }

   plot(fWeightChange(relAge)~relAge,col=colFM[1],lwd=2,type="l",las=1,ylim=c(-2.5,0.5),
        xlab="relative age, years",
        ylab="relative weight, kg")
   points(mWeightChange(relAge)~relAge,col=colFM[2],lwd=2,type="l",lty=2)
   legend("topright",legend=c("females","males"),fill=colFM)
```

# plot for the paper

```
   par(mfrow=c(2,2))
{ plot(table$avgHeightRate~avgAgeAdj[,3],col=cols,cex=2,pch=20,las=1, 
    xlim=c(-15,15),xlab="Sex-centred age, years", 
    ylab="height change, cm/yr",las=1,
    ylim=range(c(table$upperHeightRate,table$lowerHeightRate)))
   abline(a=lmHeightR$coefficients[1],b=lmHeightR$coefficients[3],col=colFM[1],lwd=2)
   segments(x0=avgAgeAdj[,3],y0=table$upperHeightRate,y1=table$lowerHeightRate,col=cols)
   abline(a=lmHeightR$coefficients[2],b=lmHeightR$coefficients[4],col=colFM[2],lwd=2,lty=2)
   legend("topright",legend=c("females","males"),fill=colFM) 
   mtext("A", side = 3, cex = 1.5, font = 2, line=0.5)

   plot(fHeightChange(relAge)~relAge,col=colFM[1],lwd=2,type="l",las=1,
        xlab="Sex-centred age, years",
        ylab="relative height, cm")
   points(mHeightChange(relAge)~relAge,col=colFM[2],lwd=2,type="l",lty=2)
   legend("topright",legend=c("females","males"),fill=colFM) 
   mtext("B", side = 3, cex = 1.5, font = 2, line=0.5)

   plot(table$avgWeightRate~avgAgeAdj[,3],col=cols,pch=20,cex=2,las=1,
       xlim=c(-15,15),xlab="Sex-centred age, years",
       ylab="weight change, kg/yr",
       ylim=range(c(table$upperWeightRate,table$lowerWeightRate)))
   segments(x0=avgAgeAdj[,3],y0=table$upperWeightRate,y1=table$lowerWeightRate,col=cols) 
   predictF=predict.lm(lmWeightR,newdata=data.frame(age.adj=relAge,age.adj2=relAge^2,sex="F"))
   points(relAge,predictF,col=colFM[1],type="l",lwd=2)
   predictM=predict.lm(lmWeightR,newdata=data.frame(age.adj=relAge,age.adj2=relAge^2,sex="M"))
   points(relAge,predictM,col=colFM[2],type="l",lty=2,lwd=2)
   legend("topright",legend=c("females","males"),fill=colFM) 
   mtext("C", side = 3, cex = 1.5, font = 2, line=0.5)

   plot(fWeightChange(relAge)~relAge,col=colFM[1],lwd=2,type="l",las=1,
        xlab="Sex-centred age, years",ylab="relative weight, kg",
        ylim=range(c(mWeightChange(relAge),fWeightChange(relAge))))
   points(mWeightChange(relAge)~relAge,col=colFM[2],lwd=2,type="l", lty=2)
   legend("topright",legend=c("females","males"),fill=colFM) 
   mtext("D", side = 3, cex = 1.5, font = 2, line=0.5)
   }
```
