# Supplementary material for: Genetic influence on within-person longitudinal change in anthropometric traits in the UK Biobank
Source: Nat Commun. 2024 May 6;15:3776. doi: 10.1038/s41467-024-47802-7 (PMC11074304; doi:10.1038/s41467-024-47802-7)
Supplement: Supplementary file 6 — Source Data [file 41467_2024_47802_MOESM6_ESM.zip › data/6_replicatevQTL/validateLociEffectsV2.html]

validation of population-level vQTL discovered in UKBiobank using individual-level variance


# validation of population-level vQTL discovered in UKBiobank using individual-level variance

#### by *Kathryn Kemper* - 11 March 2024

> The aim of this analysis is to validate the vQTL discovered in the UK
> Biobank using individual-level variance, and assess the impact of the
> mean-variance relationship on the discovered loci.

vQTL were identified using the method of Wang et al. 2019 (Levines
test for heterogeneous variance with median) for 7 weight related
traits.

There were a total of 70 loci identified with \(P\ <\ 1x10^{-8}\) across all traits.
There are 54 independent genomic locations, with several loci affecting
multiple traits.

```
    sig2=NULL 
  for (i in c(2:7)) {
            sig2=cbind(sig2,data[data$traitName==names[i],"sig"])
  }
  colnames(sig2)=names[2:7] ; sig2=data.frame(sig2) 
    upset(data=sig2, intersect=names[2:7])+
    labs(title = "Co-occurence of vQTL")+
    xlab("number of significant loci")
```

### 1. Validation of population-level vQTL with individual variance effects

The relationship between vQTL discovered between individuals and
within individuals across multiple traits. WHR and fat were excluded as
they only had few significant SNPs.

We first tested if the intercept and slopes were significantly
different between the traits, they were not.

Then make Figure 5.

```
   set1=data[data$sig&data$traitName!="WHR"&data$traitName!="Fat",]
   set1$x=set1$vb_beta
   set1$y=set1$vw_beta
   anova(lm(y~traitName+x+x:traitName,data=set1)) #different intercepts ns, slope interaction with trait ns
```

```
## Analysis of Variance Table
## 
## Response: y
##             Df    Sum Sq   Mean Sq F value    Pr(>F)    
## traitName    3 0.0006582 0.0002194  1.4639    0.2342    
## x            1 0.0082645 0.0082645 55.1428 6.845e-10 ***
## traitName:x  3 0.0006899 0.0002300  1.5344    0.2156    
## Residuals   56 0.0083929 0.0001499                      
## ---
## Signif. codes:  0 '***' 0.001 '**' 0.01 '*' 0.05 '.' 0.1 ' ' 1
```

```
   m=lm(y~x,data=set1)
   summary(m)
```

```
## 
## Call:
## lm(formula = y ~ x, data = set1)
## 
## Residuals:
##       Min        1Q    Median        3Q       Max 
## -0.030196 -0.008367 -0.001455  0.006210  0.050901 
## 
## Coefficients:
##              Estimate Std. Error t value Pr(>|t|)    
## (Intercept) 0.0009806  0.0015885   0.617    0.539    
## x           0.4903453  0.0674912   7.265 7.46e-10 ***
## ---
## Signif. codes:  0 '***' 0.001 '**' 0.01 '*' 0.05 '.' 0.1 ' ' 1
## 
## Residual standard error: 0.01252 on 62 degrees of freedom
## Multiple R-squared:  0.4599, Adjusted R-squared:  0.4511 
## F-statistic: 52.78 on 1 and 62 DF,  p-value: 7.46e-10
```

```
   #make fig 5
   slope=data.frame(slope=coef(m)[2]) ; slope$traitName="all"
   eq=lm_eqn(m,0,1) ; slope$eq=eq

   fig5 = ggplot(set1,aes(x=x,y=y))+
     geom_point(aes(color=traitName,shape=traitName),size=3)+
     geom_smooth(method="lm",formula=y~x,color="orange",fill="orange")+    
     scale_color_manual(values=brewer.pal(n = 4, name = "Set1"))+
     xlab("population_vQTL_effect") + ylab("individual_vQTL_effect") +
     geom_hline(yintercept=0,linetype="dashed",color="grey")+
     geom_vline(xintercept=0,linetype="dashed",color="grey")+
     geom_text(data=slope,aes(x=0,y=-0.05,label=eq),parse=TRUE,inherit.aes=FALSE)+
     theme_classic(base_size=20)
   fig5
```

```
   ggsave("fig5.jpg", width=7, height=4)
```

Since the traits are correlated, and also the SNP are not
independent; we conducted a sensitivity analysis by performing a
regression in each trait independently. That is, it is likely because
each data point is not independent that the P-value for the regression
slope is biased. However, each independent regression coefficient were
also significantly different from zero (P < 0.05).

Taken together, the results imply replication of the population-level
vQTL using within-individual variability.

```
   summary(lm(y~x,data=subset(set1,traitName=="BMI")))
```

```
## 
## Call:
## lm(formula = y ~ x, data = subset(set1, traitName == "BMI"))
## 
## Residuals:
##        Min         1Q     Median         3Q        Max 
## -0.0248442 -0.0046664 -0.0004617  0.0057464  0.0271333 
## 
## Coefficients:
##               Estimate Std. Error t value Pr(>|t|)    
## (Intercept) -0.0003835  0.0023880  -0.161    0.874    
## x            0.6100661  0.0982829   6.207 4.61e-06 ***
## ---
## Signif. codes:  0 '***' 0.001 '**' 0.01 '*' 0.05 '.' 0.1 ' ' 1
## 
## Residual standard error: 0.01097 on 20 degrees of freedom
## Multiple R-squared:  0.6583, Adjusted R-squared:  0.6412 
## F-statistic: 38.53 on 1 and 20 DF,  p-value: 4.609e-06
```

```
   summary(lm(y~x,data=subset(set1,traitName=="HC")))
```

```
## 
## Call:
## lm(formula = y ~ x, data = subset(set1, traitName == "HC"))
## 
## Residuals:
##        Min         1Q     Median         3Q        Max 
## -0.0197987 -0.0037610 -0.0006275  0.0039140  0.0156336 
## 
## Coefficients:
##              Estimate Std. Error t value Pr(>|t|)   
## (Intercept) 0.0004422  0.0020118   0.220   0.8290   
## x           0.3195041  0.0919784   3.474   0.0034 **
## ---
## Signif. codes:  0 '***' 0.001 '**' 0.01 '*' 0.05 '.' 0.1 ' ' 1
## 
## Residual standard error: 0.008143 on 15 degrees of freedom
## Multiple R-squared:  0.4458, Adjusted R-squared:  0.4089 
## F-statistic: 12.07 on 1 and 15 DF,  p-value: 0.003402
```

```
   summary(lm(y~x,data=subset(set1,traitName=="WC")))
```

```
## 
## Call:
## lm(formula = y ~ x, data = subset(set1, traitName == "WC"))
## 
## Residuals:
##       Min        1Q    Median        3Q       Max 
## -0.014760 -0.006486  0.000712  0.004721  0.018683 
## 
## Coefficients:
##              Estimate Std. Error t value Pr(>|t|)  
## (Intercept) -0.001940   0.002834  -0.685   0.5090  
## x            0.334856   0.117581   2.848   0.0173 *
## ---
## Signif. codes:  0 '***' 0.001 '**' 0.01 '*' 0.05 '.' 0.1 ' ' 1
## 
## Residual standard error: 0.00976 on 10 degrees of freedom
## Multiple R-squared:  0.4478, Adjusted R-squared:  0.3926 
## F-statistic:  8.11 on 1 and 10 DF,  p-value: 0.01731
```

```
   summary(lm(y~x,data=subset(set1,traitName=="Weight")))
```

```
## 
## Call:
## lm(formula = y ~ x, data = subset(set1, traitName == "Weight"))
## 
## Residuals:
##       Min        1Q    Median        3Q       Max 
## -0.026845 -0.010079 -0.003000  0.003154  0.046434 
## 
## Coefficients:
##             Estimate Std. Error t value Pr(>|t|)  
## (Intercept) 0.006545   0.005375   1.218   0.2488  
## x           0.617225   0.225788   2.734   0.0195 *
## ---
## Signif. codes:  0 '***' 0.001 '**' 0.01 '*' 0.05 '.' 0.1 ' ' 1
## 
## Residual standard error: 0.01916 on 11 degrees of freedom
## Multiple R-squared:  0.4045, Adjusted R-squared:  0.3504 
## F-statistic: 7.473 on 1 and 11 DF,  p-value: 0.01945
```

```
   # plot supp figure
   m=lm(y~-1+traitName+x:traitName,data=set1) # fit a model to get the independent coefficients
   slope=data.frame(slope=coef(m)[5:8]) ; slope$traitName=sort(unique(set1$traitName))
   eq=NULL ; for(i in 0:3) eq=rbind(eq,lm_eqn(m,i,(i+4))) ; slope$eq=eq

   x.axis=seq(-0.05,0.05,0.001) ; pred=NULL
   for (i in 1:4) {
      pred = rbind(pred,predict(m,
        newdata=data.frame(x=x.axis,traitName=unique(set1$traitName)[i]),interval="confidence",level=0.95))
   }
   pred = data.frame(traitName=rep(unique(set1$traitName),rep(length(x.axis),4)),x=rep(x.axis,4),pred)
   ggplot(set1,aes(x=x,y=y))+
           geom_ribbon(data=pred,aes(ymin = lwr, ymax = upr, x=x),inherit.aes=FALSE, alpha = 0.1) +
           geom_line(data=pred,aes(y=fit,x=x),inherit.aes=FALSE,color="blue",size=1.1) +
           geom_point(aes(color=traitName,shape=traitName),size=3)+
           geom_hline(yintercept=0,linetype="dashed",color="grey")+
           geom_vline(xintercept=0,linetype="dashed",color="grey")+
           xlab("population_vQTL_effect") + ylab("individual_vQTL_effect") +
           geom_text(data=slope,aes(x=0,y=-0.05,label=eq),parse=TRUE,inherit.aes=FALSE)+
           scale_color_manual(values=brewer.pal(n = 4, name = "Set1"))+
           theme_classic(base_size=15)+
           facet_wrap(~traitName)
```

```
## Warning: Using `size` aesthetic for lines was deprecated in ggplot2 3.4.0.
## ℹ Please use `linewidth` instead.
## This warning is displayed once every 8 hours.
## Call `lifecycle::last_lifecycle_warnings()` to see where this warning was
## generated.
```

### 2. Influence of scaling effects on the replication of vQTL

The four traits (bmi, weight, hc wc) where population-level vQTL
replicated with individual-level vQTL have a skewed distribution. We
investigated the influence of scaling on the replication of vQTL results
by transforming the data using an approximate Box Cox
transformation.

Again, we tested if the intercepts and slopes were different between
the traits. This time they were, and so we present only trait-dependent
regressions.

```
   set1=data2[data2$sig,]
   set1$x=set1$vb_beta
   set1$y=set1$vw_beta
   anova(lm(y~traitName+x+x:traitName,data=set1)) #different intercepts ns
```

```
## Analysis of Variance Table
## 
## Response: y
##             Df    Sum Sq    Mean Sq F value   Pr(>F)   
## traitName    3 0.0004203 0.00014010  1.2677 0.294309   
## x            1 0.0010130 0.00101303  9.1664 0.003722 **
## traitName:x  3 0.0008995 0.00029982  2.7129 0.053451 . 
## Residuals   56 0.0061889 0.00011052                    
## ---
## Signif. codes:  0 '***' 0.001 '**' 0.01 '*' 0.05 '.' 0.1 ' ' 1
```

```
   anova(lm(y~x+x:traitName,data=set1))     #different slopes/trait nominally sig
```

```
## Analysis of Variance Table
## 
## Response: y
##             Df    Sum Sq    Mean Sq F value   Pr(>F)   
## x            1 0.0010122 0.00101219  9.1759 0.003635 **
## x:traitName  3 0.0010012 0.00033374  3.0254 0.036524 * 
## Residuals   59 0.0065083 0.00011031                    
## ---
## Signif. codes:  0 '***' 0.001 '**' 0.01 '*' 0.05 '.' 0.1 ' ' 1
```

```
   summary(lm(y~x,data=subset(set1,traitName=="iBMI")))
```

```
## 
## Call:
## lm(formula = y ~ x, data = subset(set1, traitName == "iBMI"))
## 
## Residuals:
##       Min        1Q    Median        3Q       Max 
## -0.037811 -0.001206  0.001466  0.004860  0.022505 
## 
## Coefficients:
##               Estimate Std. Error t value Pr(>|t|)  
## (Intercept) -0.0001662  0.0026648  -0.062   0.9509  
## x            0.4618803  0.2174997   2.124   0.0464 *
## ---
## Signif. codes:  0 '***' 0.001 '**' 0.01 '*' 0.05 '.' 0.1 ' ' 1
## 
## Residual standard error: 0.01193 on 20 degrees of freedom
## Multiple R-squared:  0.184,  Adjusted R-squared:  0.1432 
## F-statistic:  4.51 on 1 and 20 DF,  p-value: 0.04637
```

```
   summary(lm(y~x,data=subset(set1,traitName=="iHC")))
```

```
## 
## Call:
## lm(formula = y ~ x, data = subset(set1, traitName == "iHC"))
## 
## Residuals:
##        Min         1Q     Median         3Q        Max 
## -0.0140021 -0.0035701 -0.0001851  0.0041738  0.0120819 
## 
## Coefficients:
##             Estimate Std. Error t value Pr(>|t|)
## (Intercept) 0.001892   0.001853   1.021    0.324
## x           0.043729   0.107203   0.408    0.689
## 
## Residual standard error: 0.007399 on 15 degrees of freedom
## Multiple R-squared:  0.01097,    Adjusted R-squared:  -0.05496 
## F-statistic: 0.1664 on 1 and 15 DF,  p-value: 0.6891
```

```
   summary(lm(y~x,data=subset(set1,traitName=="lWC")))
```

```
## 
## Call:
## lm(formula = y ~ x, data = subset(set1, traitName == "lWC"))
## 
## Residuals:
##        Min         1Q     Median         3Q        Max 
## -0.0121006 -0.0058269  0.0003099  0.0047609  0.0132205 
## 
## Coefficients:
##               Estimate Std. Error t value Pr(>|t|)
## (Intercept) -0.0005426  0.0025920  -0.209    0.838
## x            0.0577981  0.1200554   0.481    0.641
## 
## Residual standard error: 0.00892 on 10 degrees of freedom
## Multiple R-squared:  0.02265,    Adjusted R-squared:  -0.07508 
## F-statistic: 0.2318 on 1 and 10 DF,  p-value: 0.6406
```

```
   summary(lm(y~x,data=subset(set1,traitName=="lWeight")))
```

```
## 
## Call:
## lm(formula = y ~ x, data = subset(set1, traitName == "lWeight"))
## 
## Residuals:
##        Min         1Q     Median         3Q        Max 
## -0.0175025 -0.0092114 -0.0001646  0.0046211  0.0221530 
## 
## Coefficients:
##             Estimate Std. Error t value Pr(>|t|)  
## (Intercept) 0.005595   0.003522   1.589   0.1404  
## x           0.540837   0.192034   2.816   0.0168 *
## ---
## Signif. codes:  0 '***' 0.001 '**' 0.01 '*' 0.05 '.' 0.1 ' ' 1
## 
## Residual standard error: 0.01252 on 11 degrees of freedom
## Multiple R-squared:  0.419,  Adjusted R-squared:  0.3661 
## F-statistic: 7.932 on 1 and 11 DF,  p-value: 0.01678
```

```
   #make the plot
  m=lm(y~-1+traitName+x:traitName,data=set1)
  slope=data.frame(slope=coef(m)[5:8]) ; slope$traitName=sort(unique(set1$traitName))
  eq=NULL ; for(i in 0:3) eq=rbind(eq,lm_eqn(m,i,(i+4))) ; slope$eq=eq


   x.axis=seq(-0.05,0.05,0.001) ; pred=NULL
   for (i in 1:4) {
      pred = rbind(pred,predict(m,
        newdata=data.frame(x=x.axis,traitName=unique(set1$traitName)[i]),interval="confidence",level=0.95))
   }
   pred = data.frame(traitName=rep(unique(set1$traitName),rep(length(x.axis),4)),x=rep(x.axis,4),pred)
   ggplot(set1,aes(x=x,y=y))+
           geom_ribbon(data=pred,aes(ymin = lwr, ymax = upr, x=x),inherit.aes=FALSE, alpha = 0.1) +
           geom_line(data=pred,aes(y=fit,x=x),inherit.aes=FALSE,color="blue",size=1.1) +
           geom_point(aes(color=traitName,shape=traitName),size=3)+
           geom_hline(yintercept=0,linetype="dashed",color="grey")+
           geom_vline(xintercept=0,linetype="dashed",color="grey")+
           xlab("population_vQTL_effect") + ylab("individual_vQTL_effect") +
           geom_text(data=slope,aes(x=0,y=-0.05,label=eq),parse=TRUE,inherit.aes=FALSE)+
           scale_color_manual(values=brewer.pal(n = 4, name = "Set1"))+
           theme_classic(base_size=15)+
           facet_wrap(~traitName)
```
