# Supplementary material for: Genetic influence on within-person longitudinal change in anthropometric traits in the UK Biobank
Source: Nat Commun. 2024 May 6;15:3776. doi: 10.1038/s41467-024-47802-7 (PMC11074304; doi:10.1038/s41467-024-47802-7)
Supplement: Supplementary file 6 — Source Data [file 41467_2024_47802_MOESM6_ESM.zip › data/7_supplementary/SuppNote4/logisticPower.html]

Power of logistic regression to detect PRS effect


# Power of logistic regression to detect PRS effect

#### by *Kathryn Kemper* - 14 December 2023

```
#
# simulate power of logistic regression
# following https://library.virginia.edu/data/articles/simulating-a-logistic-regression-model
#

library(RColorBrewer)

simFit <- function(int,beta,n) {
  PRS = rnorm(n)
  yhat = int + beta*PRS
  p = 1/(1 + exp(-yhat))
  y <- rbinom(n, 1, p)
  mod <- glm(y ~ PRS, family = "binomial")
  c(summary(mod)$coefficients[2,4] < 0.05, cor(y,PRS))
}

powerEst <- function(N, int,beta,n){
  r.out <- replicate(n = N, simFit(int,beta,n))
  rowMeans(r.out)
}

beta = seq(-0.05,0.05,0.01)
K = c(0.001,0.005,0.01,0.02,0.03,0.05) # prevalence
power=matrix(NA,nrow=length(beta),ncol=length(K)) 
correlation=matrix(NA,nrow=length(beta),ncol=length(K))
sampleSize = 280000 ; replicates = 100
for (i in 1:length(beta)) {
    for (j in 1:length(K)) {
        beta0 = log(K[j]/(1-K[j]))
        beta1 = beta[i]
        save = powerEst(replicates,beta0,beta1,sampleSize)
        power[i,j] = save[1]
        correlation[i,j] = save[2]
    } ; }
colnames(power) = K
rownames(power) = beta
colnames(correlation) = K
rownames(correlation) = beta

# plot 1
par(cfrow=c(1,2))
```

```
## Warning in par(cfrow = c(1, 2)): "cfrow" is not a graphical parameter
```

```
cols=brewer.pal(length(K),"Dark2")
plot(power[,1]~beta,ylim=range(power),type="l",lwd=2,
     ylab="power", xlab=expression(beta[1]), col=cols[1])
abline(h=0.8,lty=2,col="grey")
for(i in 1:length(K)) points(power[,i]~beta,type="l",lwd=2,col=cols[i])
legend("top",legend=K,fill=cols)
```

```
# plot 2
plot(power[,1]~correlation[,1],ylim=range(power),type="l",lwd=2,
     ylab="power", xlab=expression(rho["y,PRS"]), col=cols[1], xlim=range(correlation))
for(i in 1:length(K)) points(power[,i]~correlation[,i],type="l",lwd=2,col=cols[i])
legend("top",legend=K,fill=cols)
```
