# Supplementary material for: Genetic influence on within-person longitudinal change in anthropometric traits in the UK Biobank
Source: Nat Commun. 2024 May 6;15:3776. doi: 10.1038/s41467-024-47802-7 (PMC11074304; doi:10.1038/s41467-024-47802-7)
Supplement: Supplementary file 6 — Source Data [file 41467_2024_47802_MOESM6_ESM.zip › data/7_supplementary/SuppNote3/SupplementaryNote3.html]

SUPPLEMENTARY NOTE 3:


# SUPPLEMENTARY NOTE 3:

### A 2-stage random regression analysis

#### Kathryn Kemper

#### 06/11/2023

---

## 3.1 Theory:

| *calculating variance components & genetic parameters (with
s.e.) on the original scale from variance components estimated using a
bivariate GREML analysis of the intercept and slope*

To calculate the variance components (e.g. genetic or phenotypic
variance) and genetic parameters (e.g. heritability) we need to
calculate the genetic and residual variance at each age, and use their
associated sampling variances to calculate the standard errors. These
can be calculated from the estimated genetic and residual
variance-covariance matrix for the mean & slope, plus the sampling
variance-covariance matrix between all the terms.

The genetic or residual variance can be calculated as a function of
the age (\(x\)) as:  
\[ f(x) = a + 2bx + cx^2 \]  
where the \(f(x)\) is the genetic or
residual variance at age \(x\), \(a\) is the genetic or residual variance
estimate for the mean, \(c\) is the
genetic or residual variance estimate for the slope and \(b\) is the genetic or residual variance
estimate of covariance between the mean and the slope.

The sampling variance of \(f(x)\)
can be computed using the variance properties of linear functions and
assuming that, at each age, \(x\) is a
constant. For example,  
\[
\begin{aligned}
var[f(x)] &= var(a) + var(2bx) + var(cx^2) + 2cov(a,2bx) +
2cov(a,cx^2) + 2cov(b,cx^2) \\
&= var(a) + 4x^2.var(b) + x^4.var(c) + 4x.cov(a,b) + 2x^2cov(a,c) +
2x^2.cov(b,c)
\end{aligned}
\]

In a similar manner, the sampling covariance between the genetic and
residual variance can be calculated using the covariance properties of
linear functions. Thus,  
\[
\begin{aligned}
cov[f\_g(x),f\_e(x)] &= cov[a\_g + 2b\_gx + c\_gx^2, a\_e + 2b\_ex + c\_ex^2
] \\
&= cov(a\_g,a\_e) + cov(a\_g,2b\_ex) + cov(a\_g,c\_ex^2) + ... \\
&= cov(a\_g,a\_e) + 2x.cov(a\_g,b\_e) + x^2.cov(a\_g,c\_e) + ...
\end{aligned}
\]

The sampling variance of the heritability estimate (i.e. a ratio) was
approximated using the delta method, following Lynch and Walsh (1998)
and Gilmore et al. (2009). That is,

\[var(\frac{\sigma^2\_n}{\sigma^2\_d}) =
(\frac{\sigma^2\_n}{\sigma^2\_d})^2 \left[
\frac{var(\sigma^2\_n)}{[\sigma^2\_n]^2} +
\frac{var(\sigma^2\_d)}{[\sigma^2\_d]^2} -
\frac{2.cov(\sigma^2\_n,\sigma^2\_d)}{\sigma^2\_n\sigma^2\_d} \right]
\]

where \(n\) and \(d\) are the numerator and denominator
respectively. Thus for the calculation of heritability, the numerator is
the estimate of the genetic variance (\(\hat{\sigma}^2\_a\)) and the denominator the
estimate of the phenotypic variance (\(\hat{\sigma}^2\_P\)). Terms were obtained by
writing out the variances and covariance in terms of the linear
function, noting that \(cov(\sigma^2\_a,\sigma^2\_P) =
cov(\hat{\sigma}^2\_a,\hat{\sigma}^2\_a+\hat{\sigma}^2\_e) =
var(\hat{\sigma}^2\_a) +
cov(\hat{\sigma}^2\_a,\hat{\sigma}^2\_e)\).

A function in R was written as follows:

```
estimateh2 <- function(x,vcov1,vcov2,samp) {
    estG <- vcov1[1,1] + 2*vcov1[2,1]*x + vcov1[2,2]*x^2
    estE <- vcov2[1,1] + 2*vcov2[2,1]*x + vcov2[2,2]*x^2
    varG <- samp[1,1] + 4*x^2*samp[2,2] + x^4*samp[3,3] +
                4*x*samp[2,1] + 2*x^2*samp[3,1] + 4*x^3*samp[3,2]
    varE <- samp[4,4] + 4*x^2*samp[5,5] + x^4*samp[6,6] +
                 4*x*samp[5,4] + 2*x^2*samp[6,4] + 4*x^3*samp[6,5]
    covGE<- samp[4,1] + 2*x*samp[5,1] + x^2*samp[6,1] +
      2*x*samp[4,2] + 4*x^2*samp[5,2] + 2*x^3*samp[6,2] +
      x^2*samp[4,3] + 2*x^3*samp[5,3] + x^4*samp[6,3]
    estP <- estG + estE
    varP <- varG + varE + 2*covGE
    h2 <- estG / estP
    varh2<- h2^2 * (varG/estG^2 + varP/estP^2 - 2*(varG+covGE)/(estG*estP))
    return(cbind(c(estG,estE,estP,h2),sqrt(c(varG,varE,varP,varh2))))
}
```

Calculation of the genetic correlation between two ages (\(x\_1\) and \(x\_2\)) followed a similar approach to the
calculation of the genetic variance and heritability estimates above.
The estimate of the genetic covariance between \(x\_1\) and \(x\_2\) is given by:  
\[ f(x\_1,x\_2) = a + b(x\_1+x\_2) + x\_1x\_2c
\]  
where \(a\), \(b\) and \(c\) are as before (the genetic variance of
the mean, slope and covariance between them).

The genetic correlation (\(r\_g\)) at
between age \(x\_1\) and \(x\_2\) is given by:  
\[ r\_g = \frac{\sigma\_{x\_1x\_2}}{\sigma\_{x\_1}
\sigma\_{x\_2} } \]

and its sampling variance by:  
\[ var(r\_g) = r\_g^2 \left[
\frac{var(\sigma^2\_{x\_1})}{4[\sigma^2\_{x\_1}]^2} +
\frac{var(\sigma^2\_{x\_2})}{4[\sigma^2\_{x\_2}]^2} +
\frac{var(\sigma\_{x\_1x\_2})}{[\sigma\_{x\_1x\_2}]^2} +
\frac{2.cov(\sigma^2\_{x\_1},\sigma^2\_{x\_2})}{4\sigma^2\_{x\_1}\sigma^2\_{x\_2}}
- \frac{2.cov(\sigma^2\_{x\_1},\sigma\_{x\_1x\_2})}{2\sigma^2\_{x\_1}\sigma\_{x\_1x\_2}}
-
\frac{2.cov(\sigma^2\_{x\_2},\sigma\_{x\_1x\_2})}{2\sigma^2\_{x\_2}\sigma\_{x\_1x\_2}}
\right ] \]

The function is written in R as:

```
rg <- function(x1,x2,vcov,samp) {
    est1 <- vcov[1,1] + 2*vcov[2,1]*x1 + vcov[2,2]*x1^2
    est2 <- vcov[1,1] + 2*vcov[2,1]*x2 + vcov[2,2]*x2^2
    est12 <- vcov[1,1] + vcov[2,1]*(x1+x2) + vcov[2,2]*x1*x2
    rg = est12 / sqrt(est1*est2)
    var1 <- samp[1,1] + 4*x1^2*samp[2,2] + x1^4*samp[3,3] +
        4*x1*samp[2,1] + 2*x1^2*samp[3,1] + 4*x1^3*samp[3,2]
    var2 <- samp[1,1] + 4*x2^2*samp[2,2] + x2^4*samp[3,3] +
        4*x2*samp[2,1] + 2*x2^2*samp[3,1] + 4*x2^3*samp[3,2]
    var12<- samp[1,1] + (x1+x2)^2*samp[2,2] + x1^2*x2^2*samp[3,3] +
        2*(x1+x2)*samp[2,1] + 2*x1*x2*samp[3,1] + 2*x1*x2*(x1+x2)*samp[3,2]
    cov1_2 <- samp[1,1] + 4*x1*x2*samp[2,2] + x1^2*x2^2*samp[3,3] +
        2*(x1+x2)*samp[2,1] + (x1^2+x2^2)*samp[3,1] + 2*x1*x2*(x1+x2)*samp[3,2] 
    cov1_12 <- samp[1,1] + 2*x1*(x1+x2)*samp[2,2] + x1^3*x2*samp[3,3] +
        (3*x1+x2)*samp[2,1] + x1*(x1+x2)*samp[3,1] + x1^2*(x1+3*x2)*samp[3,2]
    cov2_12 <- samp[1,1] + 2*x2*(x1+x2)*samp[2,2] + x2^3*x1*samp[3,3] +
        (3*x2+x1)*samp[2,1] + x2*(x1+x2)*samp[3,1] + x2^2*(x2+3*x1)*samp[3,2]
    var_rg <- rg^2 * (var1/(4*est1^2) + var2/(4*est2^2) + var12/est12^2 +
        2*cov1_2/(4*est1*est2) - 2*cov1_12/(2*est1*est12) - 2*cov2_12/(2*est12*est2))
    return(cbind(rg,sqrt(var_rg)))
}
```

## 3.2 Application:

| *using estimates from a bivariate GREML analysis as a 2-stage
RR*

We need to read in the data from the bivariate GREML analysis in
GCTA. Note here that the order of the terms is changed from the GCTA
output for ease of handling.

```
t=0 ; variances=matrix(NA,nrow=6, ncol=4) ; sampling=matrix(NA,nrow=6*4,ncol=6)
traits = c("height","weight","BMI","sit")
for (trait in traits) {
  t=t+1
  skip=60 ; if(trait=="sit") skip=61
  tmp = read.table(paste0(dir,trait,"_ageCorrected_bivar.log"), skip=skip, fill=T, nrow=6)[,2]
  variances[,t] = tmp[c(1,3,2,4,6,5)]

  skip=73 ; if(trait=="sit") skip=74
  tmp = as.matrix(read.table(paste0(dir,trait,"_ageCorrected_bivar.log"), fill=T,skip=skip,nrow=6))
  tmp = as.vector(tmp)
  a = c(1,3,2,4,6,5) ; a=rep(a,6)
  b=NULL ; for(i in 1:6) b=c(b,rep(a[i],6))
  for (i in 1:length(tmp)) sampling[((t-1)*6)+a[i],b[i]]=tmp[i] #reorder rows/columns & save
}
colnames(variances) = traits
rownames(variances) = c("G_mean","G_cov","G_slope","E_mean","E_cov","E_slope")
round(variances,3)
```

```
##         height  weight    BMI    sit
## G_mean  19.718  46.075  4.494  4.661
## G_cov   -0.012   0.086  0.018 -0.006
## G_slope  0.000   0.020  0.003  0.001
## E_mean  17.877 116.186 13.204  5.643
## E_cov    0.011   0.467  0.081  0.001
## E_slope  0.027   0.611  0.076  0.067
```

The second step is to use the functions written above to calculate
the variance components.

```
relAge = -15:15   # relative ages, relative to a mean of approx. 60 years
table=NULL
for(i in 1:4) {
  K = matrix(variances[c(1,2,2,3),i],nrow=2)
  E = matrix(variances[c(4,5,5,6),i],nrow=2)
  t = (i-1)*6
  sampling1 = sampling[(t+1):(t+6),1:6]
  for (j in relAge) table=rbind(table,estimateh2(j,K,E,sampling1))
}
table = data.frame(trait=rep(traits,each=length(relAge)*4),
           relAge = rep(rep(relAge,each=4),4) ,
           type=rep(c("G","E","P","h2"),length(relAge)*4),
           table)
names(table)[4:5] = c("estimate","se")
table$upperCI = table$estimate + 1.96*table$se
table$lowerCI = table$estimate - 1.96*table$se
table$approxAge = table$relAge + 60
```

## 3.3 Application:

#### 3.3.1 Variance components across age

Note: with 95% confidence intervals.

```
  table1 = table[table$type!="h2",]
  ggplot(table1,aes(x=approxAge,y=estimate,col=type)) + 
     geom_point(pch=4) +
     scale_color_manual(values=c("#999999", "#E69F00", "#56B4E9")) +
     geom_errorbar(aes(ymin=lowerCI, ymax=upperCI,col=type), width=.2) +
     facet_wrap(trait~., scales="free_y") + 
     ylab("trait variance") + xlab("age") +
     theme_grey(base_size = 20)
```

#### 3.3.2 Heritability as a function of age

```
  table1 = table[table$type=="h2",]
  ggplot(table1,aes(x=approxAge,y=estimate)) + 
     geom_point() + ylim(c(0,0.6)) +
     geom_errorbar(aes(ymin=lowerCI, ymax=upperCI), width=.2) +
     facet_wrap(.~trait, scales="free") + 
     theme_grey(base_size = 20)
```

#### 3.3.3 Genetic correlations as a function of age

```
   table=NULL ; tmp=NULL 
   for (i in 1:4) {
    t = (i-1)*6
    K = matrix(variances[c(1,2,2,3),i],nrow=2)
    genetic = sampling[(t+1):(t+3),1:3]
    tmp=rbind(tmp,cbind(relAge,-10,rg(relAge,-10,K,genetic)))
    tmp=rbind(tmp,cbind(relAge,0,rg(relAge,0,K,genetic)))
    tmp=rbind(tmp,cbind(relAge,10,rg(relAge,10,K,genetic)))
   }
   table = data.frame(trait=rep(traits,each=length(relAge)*3),tmp)
   names(table)=c("trait","x1","x2","rg","se")
   table$upperCI = table$rg + 1.96*table$se
   table$lowerCI = table$rg - 1.96*table$se
   table$x1 = table$x1 + 60
   table$x2 = table$x2 + 60
  
   ggplot(table,aes(x=x1,y=rg)) + 
     geom_point() +
     ylim(c(min(table$lowerCI),max(table$upperCI))) +
     geom_errorbar(aes(ymin=lowerCI, ymax=upperCI), width=.2) +
     facet_grid(trait~x2) +
     theme_grey() +
     xlab(expression(age~(x[2]))) + ylab(expression(genetic~correlation~(r[g])))
```

#### 3.3.4 Test of rg < 1 between young and old age for weight

```
    i = 2
    traits[i]
```

```
## [1] "weight"
```

```
    t = (i-1)*6
    K = matrix(variances[c(1,2,2,3),i],nrow=2)
    K
```

```
##           [,1]     [,2]
## [1,] 46.075183 0.085515
## [2,]  0.085515 0.019839
```

```
    genetic = sampling[(t+1):(t+3),1:3]
    genetic
```

```
##              [,1]         [,2]         [,3]
## [1,] 2.112016e+00 6.186288e-03 1.825517e-05
## [2,] 6.186288e-03 3.179971e-03 1.852516e-05
## [3,] 1.825517e-05 1.852516e-05 1.811350e-05
```

```
    Rage1 = 50 - 60 # i.e. evaluate 50 minus avg age
    Rage2 = 70 - 60 # i.e. evaluate 70 minus avg age
    est1 = rg(Rage1,Rage2,K,genetic)
    est1
```

```
##             rg          
## [1,] 0.9180206 0.0171385
```

```
    chisq1 = ((1 - est1[1])/est1[2])^2
    pchisq(chisq1, 1, lower.tail = FALSE)
```

```
## [1] 1.724014e-06
```

## References

Gilmore, A.R., Gogel, B.J., Cullis, B.R., Thompson, R. (2009) ‘ASReml
User Guide Release 3.0’, VSN International Ltd, Hemel Hempstead, UK.

Lynch, M. and Walsh, B. (1998) ‘Genetics and Analysis of Quantitative
Traits’, Sinauer Associates, Inc., Sunderland.
