# Supplementary figures and images for: Genetic influence on within-person longitudinal change in anthropometric traits in the UK Biobank
Source: Nat Commun. 2024 May 6;15:3776. doi: 10.1038/s41467-024-47802-7 (PMC11074304; doi:10.1038/s41467-024-47802-7)

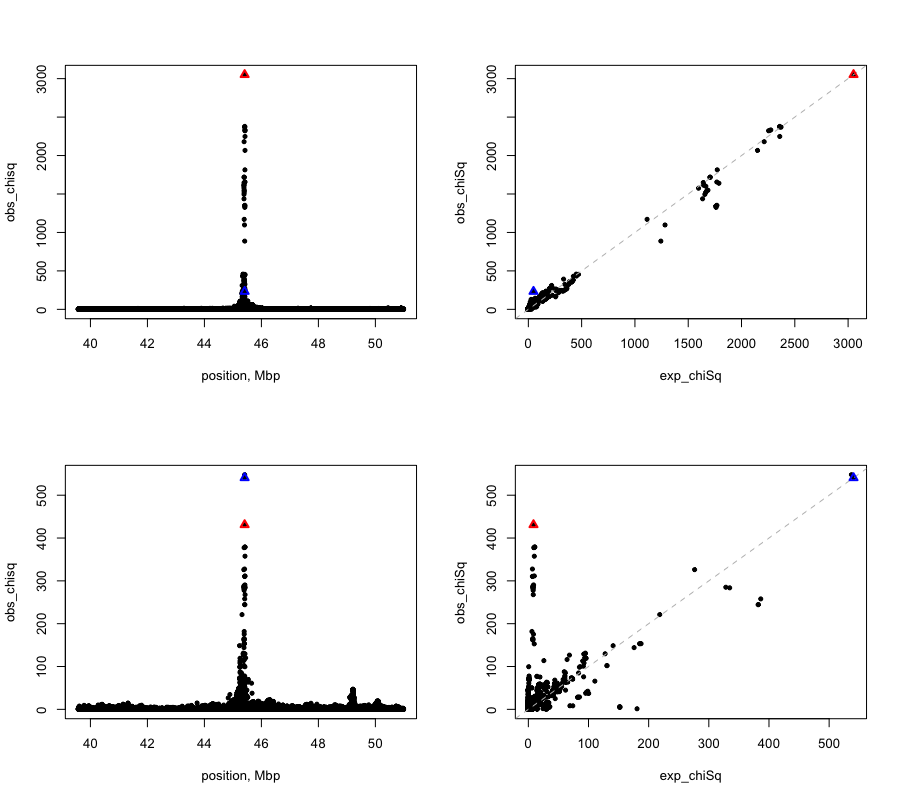

Supplement: Supplementary file 6 — Source Data [file 41467_2024_47802_MOESM6_ESM.zip › data/7_supplementary/SuppFigS3/APOE_AD_meds.png]
